# Identifying targets of multiple co-regulating transcription factors from expression time-series by Bayesian model comparison

**DOI:** 10.1186/1752-0509-6-53

**Published:** 2012-05-30

**Authors:** Michalis K Titsias, Antti Honkela, Neil D Lawrence, Magnus Rattray

**Affiliations:** 1 The Wellcome Trust Centre for Human Genetics, University of Oxford, Oxford, UK; 2 Helsinki Institute for Information Technology HIIT, Department of Computer Science, University of Helsinki, Helsinki, Finland; 3 Department of Information and Computer Science, Aalto University, Helsinki, Finland; 4 Department of Computer Science and Sheffield Institute for Translational Neuroscience, University of Sheffield, Sheffield, UK

**Keywords:** Bayesian inference, Gene regulation, Transcription factor, Gene regulatory network, Systems biology

## Abstract

**Background:**

Complete transcriptional regulatory network inference is a huge challenge because of the complexity of the network and sparsity of available data. One approach to make it more manageable is to focus on the inference of context-specific networks involving a few interacting transcription factors (TFs) and all of their target genes.

**Results:**

We present a computational framework for Bayesian statistical inference of target genes of multiple interacting TFs from high-throughput gene expression time-series data. We use ordinary differential equation models that describe transcription of target genes taking into account combinatorial regulation. The method consists of a *training* and a *prediction* phase. During the training phase we infer the unobserved TF protein concentrations on a subnetwork of approximately known regulatory structure. During the prediction phase we apply Bayesian model selection on a genome-wide scale and score all alternative regulatory structures for each target gene. We use our methodology to identify targets of five TFs regulating *Drosophila melanogaster* mesoderm development. We find that confident predicted links between TFs and targets are significantly enriched for supporting ChIP-chip binding events and annotated TF-gene interations. Our method statistically significantly outperforms existing alternatives.

**Conclusions:**

Our results show that it is possible to infer regulatory links between multiple interacting TFs and their target genes even from a single relatively short time series and in presence of unmodelled confounders and unreliable prior knowledge on training network connectivity. Introducing data from several different experimental perturbations significantly increases the accuracy.

## Background

A major challenge for computational systems biology is the inference of gene regulatory networks (GRNs) from high-throughput data such as gene expression time-series [1-5]. This is particularly challenging when the available time-series are short (i.e. contain few time points) and multiple regulators interact through cooperative or competitive mechanisms. An important first step towards uncovering regulatory networks is the identification of the targets of regulatory factors, particularly transcription factor (TF) proteins which control the transcription rate of their target genes through DNA-binding associations. In this paper we develop a computational method to infer the targets of a set of co-regulating TFs using expression time-series data from a small number of conditions. Our method is based on first learning the nature of the TF activities by focussing on a well-characterised subnetwork of targets and then performing genome-wide scans to locate other targets of the TFs. A flexible regulation model accounts for non-linear response, TF interactions and protein/mRNA degradation. A Bayesian model scoring procedure provides a principled framework for comparing alternative regulation scenarios for each putative target gene and determining the statistical support for direct regulator-target relationships.

An experimental approach to identifying TF targets might involve the design of mutant strains with the TF perturbed (knocked out, knocked down or over-expressed) and differences in the gene expression of all putative targets analyzed [6-8]. When considering multiple regulators such experiments are difficult to design since all combinations of regulators have to be probed. It can also be very difficult to differentiate between direct and indirect regulation from perturbation data. An alternative or complementary experimental approach is to discover the binding sites of regulating TFs of interest through chromatin immunoprecipitation (ChIP) experiments [9,10] (ChIP-chip or ChIP-Seq). This provides an excellent means to identify direct TF regulation. However, not all binding events show a clear relationship with gene regulation [11] and bound enhancers that are not close to a promoter region may be difficult to assign to a particular target gene. To capture transient regulatory events it is necessary to carry out a ChIP experiment in time-series [12] and this may be prohibitively costly and time consuming for multiple TFs. Gene expression time-series data therefore remain an immensely useful resource for uncovering the functional significance of regulatory interactions and to help confirm enhancer-target relationships.

Many computational methods have been introduced to infer or “reverse engineer” GRNs from time-series expression data [1,3-5]. Many of the proposed methods focus on uncovering the regulatory network for a subset of regulatory genes that are assumed to form the core of a regulatory network. This subset is typically identified as a pre-processing step, e.g. all differentially expressed or periodic TFs. Popular methods include state-space models [13], dynamic Bayesian networks [14] and ordinary differential equation (ODE) models [15-17]; see [5] for a recent review and comparative assessment on real and synthetic time-series datasets. A related but more constrained problem than GRN inference is the identification of the targets of one or a few TFs that are known to be of functional significance [18-20]. Such an approach can be applied to find targets genome-wide without very substantial filtering to reduce the set of putative targets. This target identification problem is often not aimed at identifying the full GRN model since only a limited number of TFs may be considered. However, genome-wide target identification is very useful for identifying regulated pathways or processes, or for prioritizing targets for further analysis (e.g. integrating with other evidence such as ChIP or in situ expression data) or further experiments (e.g. ChIP or perturbation experiments on high-ranking targets). An example is the work of Barenco et al. [18] who used Bayesian inference over a linear activation model to rank targets of a single TF. They considered the case of a TF activated by post-translational modification in which case a small set of known targets are required to learn the TF activity prior to ranking putative targets. In subsequent work by Gao et al. [21], Gaussian process inference techniques were developed for the same model and for non-linear generalisations (Hill kinetics activation and repression models) [21]. Honkela et al. [20,22] extended the Gaussian process method for target ranking in the case of a TF under transcriptional control by including a model of TF translation. In this case a set of known targets is not required to fit the model.

The target identification methods of Barenco et al. [18] and Honkela et al. [20] are restricted to the case of a single regulating TF. This is a useful simplification when data are limited but often TFs interact to regulate their targets through cooperative or competitive processes. Methods that ignore such interactions may have reduced accuracy in identifying targets and cannot be used to identify co-regulation of targets by multiple TFs. Other methods have been developed which allow for regulation by multiple regulators. A popular method is the Inferelator [15] which is based on fitting an ODE model with a sigmoidal non-linear regulation function to all putative regulator-target interactions. Sparse regression techniques are used to identify the regulatory network by setting the influence of unsupported links to zero. The Inferelator was one of the top performing methods for GRN inference in recent Dialogue for Reverse Engineering Assessments and Methods (DREAM) competitions for network inference [23] (DREAM 3 [24] and DREAM 4 [25]). Unlike other GRN inference methods for time-series data, such as state-space models [13], dynamic Bayesian networks [14] and other ODE-based methods [17], the Inferelator can be used for the more limited target identification task since it models the single layer target-regulator network in a decoupled manner. The highly efficient methods for inference developed for the Inferelator allows the model to be applied to large sets of regulating TFs, making this an attractive and highly practical tool for target inference from time-series data. The method is also quite general and can incorporate steady-state expression data from perturbation experiments.

**Figure 1:**
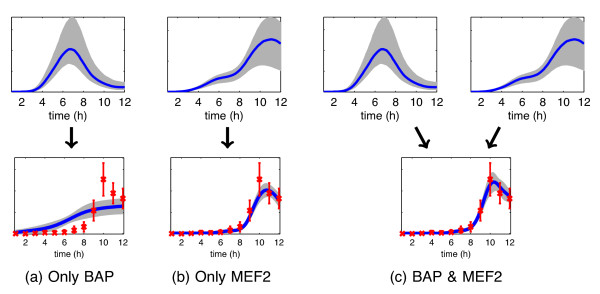
**Illustration of how two TFs can cooperatively regulate a gene.** Results are shown for a putative target gene FBgn0036752 that is highly ranked as a joint target of the TFs Bagpipe (BAP) and Myocyte enhancer factor 2 (MEF2) by the proposed method. Red crosses show target gene expression data (12 time points) from [33] and blue lines show model predictions and associated credible regions. In the top row we show the activity profiles for each TF which are inferred during the training phase by fitting a regulation model on a network of known structure. In the bottom row we show the model fit during genome-wide scanning for this target gene. We show the target mRNA concentration profile inferred by fitted models of (**a**) regulation by BAP only, (**b**) regulation by MEF2 only and (**c**) regulation by BAP and MEF2. The candidate gene is confirmed as a joint target by independent ChIP-chip studies [12].

In this contribution we show that combining the idea of a training set of known targets with a non-linear regulation model can provide a very effective method for target identification. A distinguishing feature of our work is the use of a well-characterised (but not error-free) subnetwork which is used to learn protein activities for the regulating TFs of interest (an example of the reconstructed TF activities is shown in Figure 1). This builds on the work of Barenco et al. who learned a model of TF activity from a set of known target genes [18]. We show that our method allows useful predictions to be made with only a single wild-type developmental time-series of 12 time points, thereby providing a practical tool for identifying context-specific regulatory targets. Our results show highly statistically significant enrichment for ChIP-confirmed bindings of the putative regulators in the same system and significantly better enrichment than competing methods.

A confounding aspect when applying target prediction models for a limited number of regulators is the presence of TFs that are unknown or other unmeasurable influences on the system. We show on simulated data that, despite the presence of such confounding influences, our model can reconstruct the influence of multiple regulators of interest. We also show how data from additional conditions can easily be incorporated to improve inference when available.

## Results and discussion

### Overview of the method

Our approach is based on three main components: i) the use of ODEs to model transcription, translation and mRNA/protein decay, ii) a known set of TFs that regulate transcription and iii) data-driven inference of the model parameters and network structures by using a fully Bayesian statistical method [26]. To infer TF activities over time, which can be considered functional parameters in our model, we extend previously developed Gaussian process inference techniques [20,21] to the case of multiple TFs interacting through a non-linear regulation function. Here we provide a brief description of the methodology and introduce notation that is useful for the presentation of the results. A detailed description is given in Methods and the supplementary information.

Consider the following dynamical models for the time-evolution of mRNA and TF protein abundances driven by gene transcription and TF protein translation,

(1) $$\begin{array}{ll} \text{transcription}\;\frac{\text{d}m_{j}(t)}{\text{d}t}=b_{j}+s_{j}G\left(p_{1}(t),\ldots ,p_{I}(t);\theta_{j}\right)\\ \;\;\;\;\;-d_{j}m_{j}(t)\;.\; & \; \end{array}$$

This ODE model ties together the target gene mRNA concentration *m*_*j*_(*t*), and the regulator TF protein activities *p*_*i*_(*t*). The translation model then relates the TF protein activities to the corresponding TF mRNA levels *f*_*i*_(*t*),

(2) $$\begin{array}{l} \text{translation}\;\frac{\text{d}p_{i}(t)}{\text{d}t}=f_{i}(t)-\delta_{i}p_{i}(t)\;. \end{array}$$

In the transcription equation, the TFs can jointly modulate the mRNA production rate of a target gene through the response function *G*(·) (see Methods). The equation also models mRNA degradation with rate *d*_*j*_ while *b*_*j*_ represents a basal production rate and *s*_*j*_ is a sensitivity parameter. The response function takes a sigmoidal form that non-linearly transforms the TF activities so that saturation effects are taken into account and the TFs can competitively or cooperatively activate or repress transcription [27]. The response function also depends on parameters ***θ***_*j *_which determine the network structure and regulation model coefficients. These parameters include weights that can effectively model *n*th order reactions, thus approximating the effect of, for example, TF dimerisation. Similarly, the translation equation explains the production rate of the active TF protein as a function of its mRNA while accounting for the protein degradation with rate *δ*_*i*_. We assume that the main rate-limiting step in production of active TF protein is transcription. Thus the TF activity can be considered equivalent to the TF protein concentration. This is thought to be a reasonable assumption for TFs in the *Drosophila* embryonic developmental system considered later [28] but in other systems TFs may be primarily regulated by post-translational modifications. In the Drosophila system there is significant evidence for dimerisation of the TFs, see e.g. [29-32], but no evidence of regulation by other post-translational modifications. In systems where TF activity is actively regulated by post-translational modification, e.g. through phosphorylation by a signalling pathway, then the above translation model would not correctly model changes in the concentration of active TF protein in the nucleus. However, the modelling framework that we propose can still be applied by removing the translation equations and modelling the TF protein activity as a driving latent function; see [21] for examples of this approach to TF activity inference.

**Figure 2:**
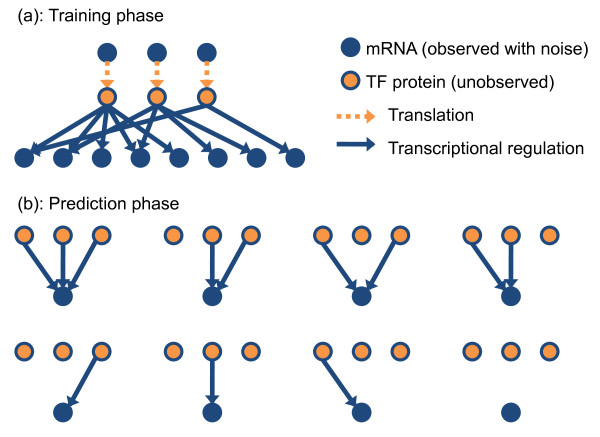
**The proposed procedure for regulatory network inference.** The procedure is divided into two phases: (**a**) The training phase involves learning the differential equation model parameters and inferring the unobserved TF protein activities on a sub-network of approximately known structure. By adopting a Bayesian inference procedure we can determine the posterior distribution over TF protein activities supported by the data. To close the system we place a Gaussian process prior distribution over the TF mRNA concentration functions [21]. (**b**) The prediction phase involves scoring all alternative regulation models for each putative target gene (2^*I *^models for *I* TFs). During this phase we assume that TF activities have a probability distribution given by the posterior distribution inferred during the training phase. The Bayesian evidence score is calculated for each regulation model and the posterior probability of any regulatory relationship of interest, such as TF–target gene associations, is determined by Bayesian model averaging.

In many experiments the protein activities, *p*_*i*_(*t*), will be difficult or impossible to measure. These continuous-time profiles must be inferred along with the parameters ***θ***_*j*_,*d*_*j*_,*b*_*j*_,*s*_*j*_ and *δ*_*i*_. Importantly, some individual parameters in ***θ***_*j *_quantify the interactions between TFs and genes and the estimation of their values allows us to infer the network structure, i.e. to identify the subset of TFs that regulate the transcription of each gene. The full continuous-time mRNA functions *m*_*j*_(bi) and *f*_*i*_(*t*) are also unobserved. A typical set-up is that we have noisy observations of these functions obtained at a set of discrete time points through gene expression analysis. Fitting the dynamical models to a biological system is carried out by the following two phases (see Figure 2):

1. Training phase: Here, we use the dynamical models to estimate the TF activities, *p*_*i*_(*t*), by using a small set of training genes. The approximate structure of this sub-network is assumed to be given so that for these genes the regulating TFs are known to some degree. All other model parameters are unknown and are inferred from the data. In this phase both the transcription model and the translation model are used to estimate the TFs. Observations associated with both the mRNA of the training genes and the TF mRNAs are required. The training phase could be carried out without the translation model in cases where TF protein activity is regulated by post-translational modification. Extensive experimentation with artificial data reveals that, when appropriate, combining a translation model with TF mRNA observations greatly aids in estimation of the TF activities.

2. Prediction phase: Once the TF activities have been estimated, each test gene (for which the regulating TFs are unknown) is processed independently and the parameters (***θ***_∗_,*d*_∗_,*b*_∗_,*s*_∗_) are inferred. Here, only the transcription model is needed while the translation model is irrelevant. This phase is applied on a genome-wide scale and aims to identify the regulating TFs for each test gene.

The above phases can be applied to a situation where prior biological knowledge provides information only about a small set of well-studied genes for which the regulating TFs are known to some degree. These genes are treated as the training data that are used to infer the activity profiles of the TFs. Typically, a full genome-wide list of targets of the TFs is unknown. This is the motivation behind the second phase which applies the trained models for genome-wide prediction of network links between genes and TFs. An important property of the second phase is that it is trivially parallelizable which allows for fast computations. The algorithms for fitting the models are based on Bayesian probabilistic inference and details are given in the supplementary information.

We will illustrate our methodology using mesoderm development in embryonic *Drosophila melanogaster*. First, though, we create an artificial example that highlights the difficulties inherent in inference of transcription networks directly from data.

### Synthetic data

**Figure 3:**
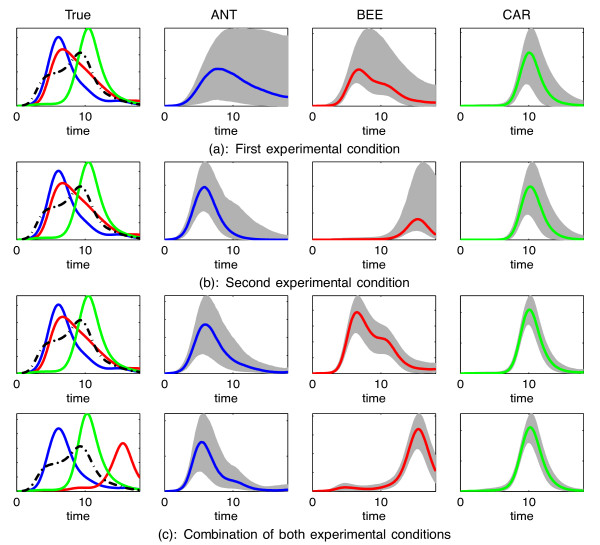
**TF concentrations inferred by the model in the synthetic data.** The plots in panel (**a**) (the four plots in the first row) illustrate the estimation of the TF activity using only the first experimental condition. The left plot shows the ground-truth TF activities that generated the observed data. In particular, the coloured solid lines show the three TFs, which were assumed to be known (blue: ANT, red: BEE, green: CAR) and the black dotted line displays the unknown factor UNK. The remaining three plots in panel (**a**) show the TFs estimated at the training modelling phase. Here, the coloured lines display the estimated means of ANT, BEE and CAR and the shaded areas show 95% credible regions. The plots in panel (**b**) display the exactly analogous plots with those of (a) with the difference that the second experimental condition was considered instead of the first. The plots in panel (**c**) illustrate the estimation of TFs by using simultaneously both experimental conditions. The plots in the first row of (c) display the estimates for the first experimental condition, while the plots of the second row display the estimates for the second experimental condition.

We consider an artificial gene network involving *four* transcription factors: ANT, BEE, CAR and UNK. We will simulate data directly from our network, but when modelling the data we will only consider *three* of these transcription factors: ANT, BEE and CAR. This reflects a realistic scenario where there is an unacknowledged confounding transcription factor (UNK) affecting our system. We simulated data associated with two experimental conditions. The data are short unevenly sampled time-series of 10 time points. In our first experimental condition there is considerable overlap between the TF concentrations of ANT and BEE as shown in Figure 3(a), while in the second experimental condition the overlap of BEE with ANT is far less (Figure 3(b)). In both experimental conditions there is considerable overlap between UNK and the three acknowledged TFs.

The purpose of our experiment with simulated data is to predict the set of regulating TFs for each gene using artificially generated mRNA measurements. Since the ground-truth network links are known, we can make a rigorous assessment of the ability of the model to identify the target genes of each of the TFs, as well as an assessment of the ability to predict non-regulation. The modelling is split into two distinct phases as described in the previous section. In the training phase, 30 genes with approximately known connectivity were used for learning the TF profiles. Specifically, to make the training phase more realistic we added 15% noise to the ground-truth network links in these 30 training genes. This resulted in 16 links between TFs and genes (in the initial ground-truth network structure) to change so that some of these links falsely became active and others were removed (i.e. from active they became inactive). Notice that this noise in the network links adds an extra model-mismatch in addition to the presence of the UNK TF which is not part of the model. In the prediction phase these profiles were used to rank other potential targets of the TFs from the remaining 1000 genes. Full details on how the data have been generated are given in Methods, while the dataset is provided together with software that is available online.

To assess the predictive ability of the model with respect to the amount of information present in the data, we consider three experiments. In the first experiment only data from the first experimental condition are used, in the second experiment only data from the second experimental condition are used, while in the third experiment all data from both conditions are considered.

#### Using data from one experimental condition

Here, we assume the synthetic mRNA data are produced by a single experimental condition, i.e. either the first or the second condition mentioned earlier. When considering the first condition the true TF profiles for ANT, BEE, CAR and UNK are shown in the left plot of Figure 3(a) and the corresponding TF mRNA functions are shown in Additional file 1: Figure S1(a). The remaining three plots in Figure 3(a) show the TF activities estimated in the training phase by using 30 genes with approximately known network connectivity and *unknown* model parameters. The coloured solid lines show the estimated means and the shaded areas represent 95% posterior credible regions around the estimated means. Plots showing how the model fits the mRNA data in the training phase are presented in Additional file 1: Figures S2 and S6 and all corresponding ODE parameters are shown in Additional file 1: Figures S9 and S10.

**Figure 4:**
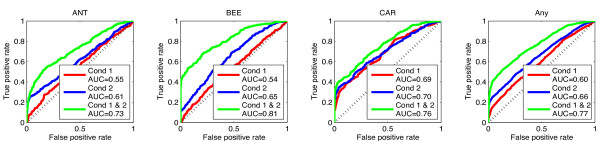
**ROC curves for predicting the network connections in the synthetic data.** Red curves show the results by using only the first experimental condition, blue curves show the results by using only the second experimental condition, while green curves correspond to the results when both experimental conditions are used. The diagonal black dotted line is the performance based on random prediction. The first three plots from the left show the ROC curves for predicting the individual TF links and the last plot shows the overall performance, i.e. for predicting any link.

Figure 3(a) shows that ANT and BEE have very similar profiles. This is a realistic scenario, but this type of ambiguity can have a negative effect on the estimated TF activities and the predictive accuracy of the model. In particular, the estimation of these two TFs, shown in the second and third plot from the left in Figure 3(a), is rather uncertain (as indicated by the very large shaded area that represents uncertainty). Moreover, the fact that the profiles of these TFs overlap significantly with each other yields poor performance when predicting the network links. The ROC curves in Figure 4 show accuracy when predicting the individual TF links (first three plots from the left) and overall performance when predicting single links (last plot). In all panels the solid red line is the ROC curve associated with the performance of the model when using the first experimental condition. Notice that for ANT and BEE the performance is only slightly better than random (diagonal dotted black line). For CAR the performance is better since the profile of this TF overlaps much less with those of ANT and BEE.

From the above experiment we can conclude that it is rather difficult to accurately predict network links between TFs and genes from experimental data obtained under conditions that do not disambiguate sufficiently the functionality of the TFs during the transcription process. Roughly speaking, the “similarity” of some TFs causes the observed mRNA data to be well explained by alternative hypotheses associated with the presence/absence of these similar TFs and makes it hard to statistically identify which of those TFs were actually driving the regulation process.

We now consider a second series of observed mRNA measurements associated with an alternative simulated experimental condition comprising a perturbation of the biological system that better disambiguates the two (previously overlapping) TFs in terms of their influence in gene transcription. We first use only these new data instead of the data associated with the first experimental condition. This alternative perturbation changes significantly the protein activity for BEE as shown on the left plot in Figure 3(b), while ANT, CAR and UNK are assumed to behave similarly to the first experimental condition. The estimated TFs are shown in the plots of the remaining three columns of Figure 3(b) and model fits in the training mRNA data for this second condition are plotted in Additional file 1: Figures S3 and S7 and all associated ODE parameters are shown in Additional file 1: Figures S11 and S12. The blue ROC curves in Figure 4 show predictive performance when using this second experimental condition. As the blue curves indicate, the performance now improves compared to the results obtained by using the first condition (red curves). This is expected since the second condition disambiguates more efficiently the TF activities than the first condition. In the next section we will see that the performance can be further improved when the models are fitted simultaneously to data from both experimental conditions.

#### Combining the data from both experimental conditions

In our third experiment we fit the models using all data from both experimental conditions. Figure 3(c) shows the TFs that generated the mRNA data for both experimental conditions (plots in the first column from the left) and the estimated TFs (plots in the remaining three columns). Each row of Figure 3(c) corresponds to each of the two conditions. Model fits in the training mRNA data are plotted in Additional file 1: Figures S4 and S8 and all associated ODE parameters are shown in Additional file 1: Figures S13 and S14.

Including data from both experimental conditions allows for a more confident estimation of the TF profiles. To see this, we can contrast the second up to fourth plots in the first row of Figure 3(c) with the corresponding plots of Figure 3(a)-(b). The credible regions when simultaneously using both experimental conditions are significantly smaller, which implies higher confidence.

Furthermore, we obtain a significant increase in the predictive performance when identifying network links. As the green coloured ROC curves in Figure 4 reveal, the performance when predicting single network links is significantly improved. Finally, we can exploit the ability of the model to predict a simultaneous regulation of the target gene by two or more TFs. Additional file 1: Figure S5 displays the predictive ROC curves for all three TF pairs in this example.

### *Drosophila* data

**Figure 5:**
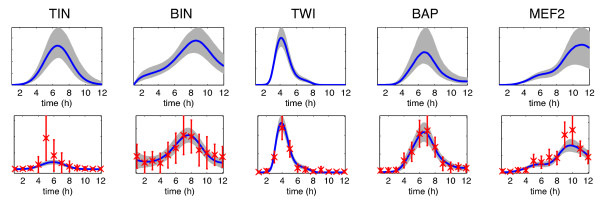
**The estimated TF activities and predicted TF mRNAs from the training modelling phase in *Drosophila*data.** The five plots in the first row display the estimated TFs of the third replica. Each blue solid line represents an estimated mean TF activity and the shaded area represents 95% credible regions around the mean. The five plots in the second row display the predicted TF mRNAs (blue solid lines and shaded areas) together with the observed data represented by red crosses (means) and vertical lines (two-standard deviations around the means provided by the microarray preprocessing stage).

In this section we apply our method to a dataset of three independently repeated time-series of 12 time points collected hourly throughout *Drosophila melanogaster* embryogenesis in wild-type embryos [33]. For preprocessing of the data we followed [20]. We study five TFs that are key regulators of mesoderm and muscle development in *Drosophila*: Tinman (TIN), Biniou (BIN), Twist (TWI), Bagpipe (BAP) and Myocyte enhancer factor 2 (MEF2) [12]. We identified an initial set of 92 genes from [12] associated with a curated subset of ChIP-bound enhancers that have well characterised effects on expression (see Methods). Many of these genes display expression profiles that cannot be fully explained using the five studied TFs. To remove these confounding targets, the training modelling phase (based on these 92 genes) was robustified as follows. We first performed a preliminary fit of a robustified model using a noise model including both a component extracted from microarray preprocessing as well as an additive learned component. We then selected genes that had sufficiently small additive learned variance (see Methods), resulting in 25 genes. These were then used in final training with only noise from preprocessing included in the model. Figure 5 shows the inferred profiles for all five TFs (first row) together with the corresponding predicted TF mRNAs (second row) for the third replica of the time-series. The TF profiles and predicted TF mRNAs for the remaining two replicas are shown in Additional file 1: Figure S15. Model fits in the training mRNA target gene data are shown in Additional file 1: Figure S18 (showing genes included in final training) and Additional file 1: Figure S19 (showing genes excluded from final training) in the supplementary information while ODE parameters are shown in Additional file 1: Figures S20 and S21.

#### Prediction of network connections

Once the TF activities have been estimated, we use the model to predict the regulator TFs for a set of 6003 test genes which exclude the 92 genes used in the training phase. A web-based browser that displays how the model fits the mRNA data of test genes is available online at [34]. Full posterior probabilities of all alternative models for all test genes are included in Additional file 2. This set includes all genes in the data that are not classified as weakly expressed according to the criterion explained previously [20]. We followed an approach to evaluation of predictive performance similar to one described in [20]. A number of predictions is evaluated by considering for each gene a predicted set of regulators correct if all TFs in the set had evidence of binding within 2000 base pairs of the corresponding gene in the ChIP-chip data in [12]. Different methods can be compared based on the corresponding percentage enrichments. It should be noted that this validation is still far from perfect since bound enhancers can regulate transcription from a distance greater than the conservative limit considered here. We also perform similar evaluation using TF-gene links in the Drosophila Interaction Database (DroID) [35]. This database in not specific to development and may thus include links that are not active in our data. We only include the 5521 test genes with some predicted TF regulators in the database. We compare two variants of our proposed method to a maximum-likelihood-based baseline method, the Inferelator 1.1 [15] and a simpler sparse regression approach (see Methods).

**Figure 6:**
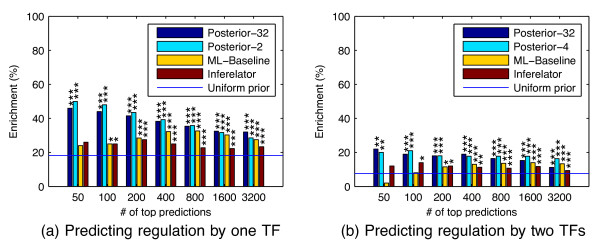
**Enrichment of confident regulator predictions for ChIP binding.** Plots show percentage of top ranked confident regulator predictions that had confirmed bindings by predicted regulators within 2000 base pairs of the putative target gene. Predictions were ranked by the posterior probability of **(a)** regulation by any single regulator; or **(b)** joint regulation by any two regulators. Both plots include rankings according to the marginal posterior probability of a set of regulators being active computed over all 32 models (dark blue bars), posterior probability over a restricted set of models ignoring all other TFs leaving 2 models for single regulator and 4 models for two regulators (light blue bars) as well as maximum likelihood-based baseline model (yellow bars) and the Inferelator (red bars), compared to predicting regulators uniformly at random (blue line; link probability 0.5). *p*-values of results statistically significantly different from random are denoted by ‘***’: *p*<0.001, ‘**’: *p*<0.01, ‘*’:*p*<0.05.

In the plots of Figure 6, we consider inferring single TF and TF-pair regulators with ChIP evaluation. The single-TF ranking is constructed by computing for each gene the marginal posterior probability of the event that a certain TF is a regulator. Since we have five TFs, there are five probabilities of this type for each gene. We compute the posterior probabilities in two ways: either averaging over all models weighted by their marginal likelihood (“Posterior-32”) or using just the selected and null models (“Posterior-2”). The resulting 5 × 6003 probabilities are sorted in decreasing order and Figure 6(a) displays the enrichment results at different cutoffs of this list. The predictions of both these methods are significantly better than random (*p *< 0.01 or less in all cases using tail probability in a hypergeometric distribution) and clearly outperform the maximum likelihood baseline and the Inferelator. We also carried out empirical bootstrap tests for each pair-wise comparison of methods which confirm that the proposed methods outperform the other methods statistically significantly in most cases (see Table 1).

**Table 1:** Link-specific ChIP evaluation bootstrap results

**Predicting regulation by single TFs**														
**Top 50**					**Top 100**					**Top 200**				
	P32	P2	ML	Inf		P32	P2	ML	Inf		P32	P2	ML	Inf
P32			***	*	P32			**	**	P32			**	**
P2			***	**	P2	+		***	***	P2			***	**
ML					ML					ML				
Inf					Inf					Inf				
**Top 400**					**Top 800**					**Top 1600**				
	P32	P2	ML	Inf		P32	P2	ML	Inf		P32	P2	ML	Inf
P32			*	***	P32			+	***	P32			*	***
P2			**	***	P2			*	***	P2			*	***
ML				*	ML				***	ML				***
Inf					Inf					Inf				
					**Top 3200**									
						P32	P2	ML	Inf					
					P32		***	***	***					
					P2			*	***					
					ML				***					
					Inf									
**Predicting regulation by TF pairs**														
**Top 50**					**Top 100**					**Top 200**				
	P32	P4	ML	Inf		P32	P4	ML	Inf		P32	P4	ML	Inf
P32			**	+	P32			*	.	P32			+	*
P4			**	+	P4			*	+	P4			+	*
ML					ML					ML				
Inf			.		Inf					Inf				
**Top 400**					**Top 800**					**Top 1600**				
	P32	P4	ML	Inf		P32	P4	ML	Inf		P32	P4	ML	Inf
P32			*	**	P32			+	***	P32				**
P4			+	**	P4			*	***	P4	**		*	***
ML					ML				.	ML				+
Inf					Inf					Inf				
					**Top 3200**									
						P32	P4	ML	Inf					
					P32				*					
					P4	***		**	***					
					ML	*			***					
					Inf									

The results of 100,000-fold bootstrap resampling of the data set of observed genes to assess significance of differences in ranking method performance. The methods studied are: P32 = Posterior-32 method, P2 = Posterior-2 method, P4 = Posterior-4 method, ML = ML Baseline method, Inf = Inferelator. For each pair of methods, the marks in the tables show how often the method on the corresponding row dominated the one on the corresponding column. The marks are interpreted as follows: ‘.’: >80*%*dominance, ‘+’: >90*%*dominance, ‘*’: > 95*%*dominance, ‘**’: >99*%*dominance, ‘***’: >99.9*%*dominance, ‘-’: comparison not applicable.

For the TF-pair regulator rankings, we compute the marginal posterior probabilities for all possible pairs of TFs for each gene. The counterpart of Posterior-2 now includes four models: the pair, both partners individually and the null, and is denoted by “Posterior-4”. Otherwise the ranking lists are computed exactly as in the case of single-TF regulators but now for the 10×6003 possible TF-pair models. Figure 6(b) displays the results. The figure again shows statistically highly significant enrichment of binding of predicted regulators near the corresponding target genes. The enrichment is lower than it was for single-TF predictions, which is expected since the task of identifying regulating pairs of TFs is harder but may also be partly due to an increased number of false negatives in the validation data. The Bayesian methods based on posterior probabilities are consistently more accurate than the maximum likelihood baseline. In most cases the more restricted set of models seems to yield better results. Nevertheless, there are some TFs for which the opposite is true, as illustrated by the corresponding results, broken down for each TF, that are shown in Additional file 1: Figures S16 and S17. This may be because the more restricted posterior probabilities are less sensitive to misspecification of prior probabilities of network links. Currently all TFs are assumed to regulate every gene with prior probability 0.5, which is unrealistic. Unfortunately it is nontrivial to construct better alternatives without significant extra information because the TFs are heavily correlated. We did not wish to use the ChIP data for constructing such a prior since this was required as independent data for validating the results.

**Figure 7:**
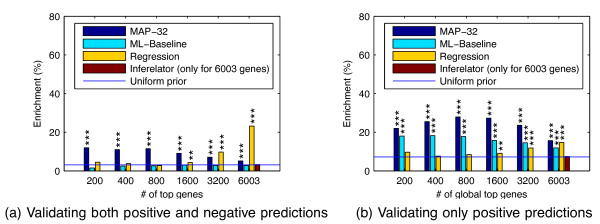
**Enrichment of binding of predicted regulator TFs near genes.** The plots show the percentage enrichment of top-ranking genes with ChIP-chip evidence of binding of all the predicted regulators. In (**a**) only genes with exactly correct binding profile (both positives and negatives) are considered correct predictions. In (**b**) only positive predictions are required to be correct and additional bound TFs are ignored, but genes predicted to be unregulated are ignored completely. Genes are ranked by the posterior probability of the most likely model. The compared methods are the posterior probability over all 32 models (dark blue bars), the maximum likelihood-based baseline method (light blue bars), the Regression method (yellow bars) and the Inferelator (red bars), which are compared to predicting regulators uniformly at random (blue line; link probability 0.5). As the Inferelator offers no clear method for ranking the genes, its results are only shown for all 6003 genes. *p*-values of results statistically significantly different from random are denoted by ‘***’: *p *< 0.001, ‘**’: *p *< 0.01, ‘*’: *p *< 0.05.

We also compute the *a posteriori* most probable regulator model for each gene, which we refer to as the maximum a posteriori (MAP) model. Figure 7 shows results of the ChIP evaluation based on the MAP regulator configuration for every gene, ranked by the posterior probability of this most probable model. Because there is no clear way to rank the genes with the Inferelator, the accuracy is only shown for the complete list of all genes. Additionally we compare the results against a more straightforward sparse regression method (“Regression”; see Methods for details).

Figure 7(a) displays results for full validation of both positive and negative predictions. The results of the proposed method are statistically very significantly better than random, while the maximum likelihood baseline and the Inferelator are no better than random guessing. The regression method does poorly at first but ends with a much higher accuracy than all others. The main reason for this is that it makes a higher fraction of negative predictions; all other methods make many fewer predictions for genes being unregulated by all TFs while such cases are fairly common based on our validation data. This behaviour is expected for the probabilistic method, which has a uniform prior over regulating TF combinations. Under this prior, the prior probability for a gene to be unregulated is only 1/32. If a more sensible prior is used, for example, by considering the empirical prior from the binding frequencies in the validation data, the proposed method can attain even higher accuracy than the regression method (results not shown). According to the bootstrap testing, the proposed method is statistically significantly better than the alternatives in all cases except regression with ≥3200 top predictions (*p *< 0.01; see Table 2 for full results).

**Table 2:** Full model ChIP evaluation bootstrap results

**Validating both positive and negative predictions**														
**Top 200**					**Top 400**					**Top 800**				
	MAP	ML	Reg	Inf		MAP	ML	Reg	Inf		MAP	ML	Reg	Inf
MAP		***	**	-	MAP		***	***	-	MAP		***	***	-
ML				-	ML				-	ML				-
Reg		+		-	Reg				-	Reg				-
Inf	-	-	-	-	Inf	-	-	-	-	Inf	-	-	-	-
**Top 1600**					**Top 3200**					**Top 6003**				
	MAP	ML	Reg	Inf		MAP	ML	Reg	Inf		MAP	ML	Reg	Inf
MAP		***	***	-	MAP		***		-	MAP		***		***
ML				-	ML				-	ML				
Reg		*		-	Reg	***	***		-	Reg	***	***		***
Inf	-	-	-	-	Inf	-	-	-	-	Inf		*		
**Validating only positive predictions**														
**Top 200**					**Top 400**					**Top 800**				
	MAP	ML	Reg	Inf		MAP	ML	Reg	Inf		MAP	ML	Reg	Inf
MAP		.	***	-	MAP		**	***	-	MAP		***	***	-
ML			**	-	ML			***	-	ML			***	-
Reg				-	Reg				-	Reg				-
Inf	-	-	-	-	Inf	-	-	-	-	Inf	-	-	-	-
**Top 1600**					**Top 3200**					**Top 6003**				
	MAP	ML	Reg	Inf		MAP	ML	Reg	Inf		MAP	ML	Reg	Inf
MAP		***	***	-	MAP		***	***	-	MAP		***	*	***
ML			***	-	ML			***	-	ML				***
Reg				-	Reg				-	Reg		***		***
Inf	-	-	-	-	Inf	-	-	-	-	Inf				

The results of 100,000-fold bootstrap resampling of the data set of observed genes to assess significance of differences in ranking method performance. The methods studied are: MAP = MAP GP method, ML = ML Baseline method, Reg = regression baseline method, Inf = Inferelator. For each pair of methods, the marks in the tables show how often the method on the corresponding row dominated the one on the corresponding column. The marks are interpreted as follows: ‘.’: >80*%*dominance, ‘+’: >90*%*dominance, ‘*’: >95*%*dominance, ‘**’: >99*%*dominance, ‘***’: >99.9*%*dominance, ‘-’: comparison not applicable.

Because of frequent non-functional binding [11], it makes sense to ignore additional bound TFs. In this case negative predictions cannot be validated, only positive ones. Figure 7(b) shows the validation results in this case. Genes with a MAP model with no regulation were ignored because they would all be judged as “correct” here, biasing the accuracy results. The figure again shows statistically significant enrichment of binding of predicted regulators near the target genes. The proposed Bayesian method based on posterior probabilities is clearly more accurate than the maximum likelihood baseline and also more accurate than the regression method in all cases. According to the bootstrap testing, the proposed method is statistically significantly better than the alternatives in all cases except maximum likelihood baseline 200 top predictions (*p *< 0.01, except *p *< 0.05 for regression with 6003 top predictions; see Table 2 for full results). The computation times of the different alternatives are listed in Table 3.

**Table 3:** Running times

**MAP-32**	**Baseline**	**Regression**	**Inferelator**
5911.50	2236.36	0.96	0.25

Running computer times (in seconds) of different methods for scoring all possible 32 models (combinations of five TFs) in a single target gene (out of the 6003 genes) in the *Drosophila* data.

**Figure 8:**
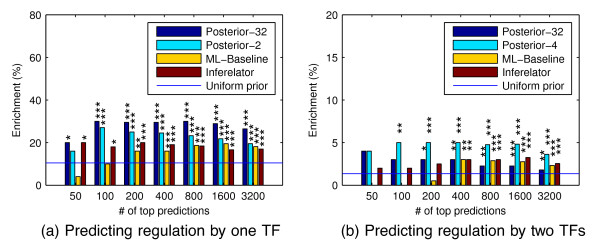
**Enrichment of confident regulator predictions for DroID interactions.** Similar to Figure 6 but using DroID database TF-gene interactions instead of ChIP binding for validation.

**Figure 9:**
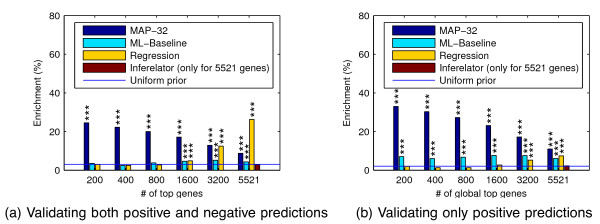
**Enrichment DroID interactions predicted regulator TFs.** Similar to Figure 7 but using DroID database TF-gene interactions instead of ChIP binding for validation.

Similar evaluation for DroID validation is shown in Figures 8 and 9. In Figure 8 the relative order of the methods is mostly the same as in Figure 6, but the percentage enrichments of all methods are significantly lower. This may be due to incompleteness of the DroID database. The number of annotated TF-gene interactions in DroID is roughly similar to the number of genes with ChIP binding for TWI, but much lower for all other TFs. The number of genes with more than one regulator is even more significantly lower in DroID. As the ChIP data was gathered using the same protocol for all TFs, it seems more likely to contain balanced information for all TFs. Nevertheless, the most probable regulator combination results in Figure 9 show very high accuracy for our MAP method, which is very clearly superior to all other methods, except regression when using the full list of genes. Bootstrap testing results are presented in Tables 4 and 5.

**Table 4:** Link-specific DroID evaluation bootstrap results

**Predicting regulation by single TFs**														
**Top 50**					**Top 100**					**Top 200**				
	P32	P2	ML	Inf		P32	P2	ML	Inf		P32	P2	ML	Inf
P32			***		P32		.	***	*	P32		*	***	**
P2			**		P2			***	+	P2			***	+
ML					ML					ML				
Inf			*		Inf			*		Inf			.	
**Top 400**					**Top 800**					**Top 1600**				
	P32	P2	ML	Inf		P32	P2	ML	Inf		P32	P2	ML	Inf
P32		*	***	***	P32		***	***	***	P32		***	***	***
P2			***	*	P2			***	**	P2			***	***
ML					ML					ML				*
Inf			.		Inf					Inf				
					**Top 3200**									
						P32	P2	ML	Inf					
					P32		***	***	***					
					P2			**	**					
					ML				+					
					Inf									
**Predicting regulation by TF pairs**														
**Top 50**					**Top 100**					**Top 200**				
	P32	P4	ML	Inf		P32	P4	ML	Inf		P32	P4	ML	Inf
P32			***	.	P32			***		P32			*	
P4			***	.	P4			***	.	P4	.		**	+
ML					ML					ML				
Inf					Inf			.		Inf			+	
**Top 400**					**Top 800**					**Top 1600**				
	P32	P4	ML	Inf		P32	P4	ML	Inf		P32	P4	ML	Inf
P32					P32					P32				
P4	*		*	*	P4	***		*	*	P4	***		**	*
ML					ML					ML	.			
Inf					Inf					Inf	*			
					**Top 3200**									
						P32	P4	ML	Inf					
					P32									
					P4	***		**	**					
					ML	+								
					Inf	*								

Same as Table 1 but for DroID evaluation.

**Table 5:** Full model DroID evaluation bootstrap results

**Validating both positive and negative predictions**														
**Top 200**					**Top 400**					**Top 800**				
	MAP	ML	Reg	Inf		MAP	ML	Reg	Inf		MAP	ML	Reg	Inf
MAP		***	***	***	MAP		***	***	***	MAP		***	***	***
ML					ML					ML			.	.
Reg					Reg					Reg				
Inf					Inf					Inf				
**Top 1600**					**Top 3200**					**Top 6003**				
	MAP	ML	Reg	Inf		MAP	ML	Reg	Inf		MAP	ML	Reg	Inf
MAP		***	***	***	MAP		***		***	MAP				
ML				**	ML				***	ML				
Reg				**	Reg		***		***	Reg				
Inf					Inf					Inf				
**Validating only positive predictions**														
**Top 200**					**Top 400**					**Top 800**				
	MAP	ML	Reg	Inf		MAP	ML	Reg	Inf		MAP	ML	Reg	Inf
MAP		***	***	***	MAP		***	***	***	MAP		***	***	***
ML			**	+	ML			***	*	ML			***	***
Reg					Reg					Reg				
Inf					Inf			+		Inf			*	
**Top 1600**					**Top 3200**					**Top 6003**				
	MAP	ML	Reg	Inf		MAP	ML	Reg	Inf		MAP	ML	Reg	Inf
MAP		***	***	***	MAP		***	***	***	MAP				
ML			***	***	ML			***	***	ML				
Reg					Reg				***	Reg				
Inf					Inf					Inf				

Same as Table 2 but for DroID evaluation.

#### Parameter estimates

The protein degradation rates and the corresponding protein half-life estimates from the model are presented in Table 6. The estimates are unusually short for proteins in general, but they are in line with recent research demonstrating that Twist homologue has a very short half-life in the mouse [36]. As other studied TFs are from the same protein family, it is plausible they could share similar half-lives. Cell division also contributes to the effective degradation rate and it is also possible that diversification during development can lead to a higher effective decay rate since the proportion of cells with tissue-specific TF activity reduces over time. These effects will also increase the effective target mRNA degradation rates.

**Table 6:** Inferred protein degradation rates

	**Degr. (1/h)**	**half-life (h)**
TIN	(0.81, 1.19, 1.66)	(0.42, 0.58, 0.86)
BIN	(0.62, 0.80, 1.08)	(0.64, 0.87, 1.12)
TWI	(3.79, 4.62, 5.79)	(0.12, 0.15, 0.18)
BAP	(3.08, 5.41, 8.27)	(0.08, 0.13, 0.23)
MEF2	(0.57, 0.80, 1.67)	(0.41, 0.86, 1.20)

Inferred protein degradation rates (first row) for the five TFs and the corresponding estimates for the half-life of each protein (second row). Recall that the formula for the half-life is log(2)/δ where *δ *is protein degradation rate. Each triple (*a,b,c*) of values corresponds to 5% percentile, median and 95% percentile.

## Discussion

It may be thought that a typical short time-series expression dataset contains only very limited information about the structure of a GRN. In a meta-analysis of methods proposed in the DREAM 2 competition [37], the authors in [4] found time-series data to be much less informative for network inference than data from a similar number of perturbation experiments. However, in the datasets considered there many of the time-series experiments are rather uninformative about expression changes given the level of noise in the data and uninformative selection of sampled time points. We would argue that the success of a method for analysis of time-series data will depend greatly on how informative the profiles of the regulatory species are. In our synthetic example we clearly demonstrated how inference is sensitive to confounding by highly similar temporal profiles of regulating TFs, so it is certainly desirable to have access to data from diverse experimental conditions where available. Yet with an animal system the available perturbations may be severely limited and the wild-type under normal conditions is of great interest for understanding healthy function. Methods for learning the structure of a regulatory network from one or a few short time course experiments are of great practical importance for uncovering a condition-specific GRN.

Many methods for the inference of GRNs from gene expression data require much more data, and data from a much greater diversity of experiments, than we consider here [1-3]. However, several approaches have been proposed for identifying the targets of a specific TF given data from time-series experiments collected under one or two conditions [18-20]. The methods in [18] and [20] rank targets by fitting simple linear activation differential equation models for a single regulating TF. These methods do not account for the more general and realistic scenario of non-linear regulation by multiple TFs. The method in [19] does allow for regulation by other unknown factors, modelled by fitting a sparse linear regression model, but assumes measurements of the TF protein are available. Here we introduced a much more general method, where a model of non-linear regulation by multiple TFs is used to predict which set of TFs regulate each putative target on a genome-wide scale. Bayesian inference methods provide a principled approach for (i) dealing with an underdetermined inference problem by Bayesian parameter averaging, (ii) scoring alternative networks by Bayesian model selection and (iii) predicting TF-target associations by Bayesian model averaging. Our results demonstrate that even with very limited time-series information the method is able to correctly identify which of the closely related TFs regulate the given target. This is clearly a more challenging task that is not addressed in [18] and [20]. Additional information, even just independent estimates of decay rates of different transcripts, would certainly make the task easier, as demonstrated in [38] and also our results on synthetic data.

The Inferelator is an effective method for target identification which also uses a non-linear regulation model that accounts for regulation by multiple TFs [15]. The Inferelator is applicable more generally since it uses less prior information about the system than we are assuming. Two important assumptions were made in the analysis of the *Drosophila* data; we assumed knowledge of a well-characterised sub-network of the GRN, which is used to learn the TF activity profiles during the training phase, and in the present application we restrict ourselves to models of activation. Our results demonstrate improved performance over the Inferelator but it should be acknowledged that we are solving a more restricted class of problem. Our method is also much more computationally demanding (see Table 3); it is applicable to genome-wide scanning for a small set of TFs but would not be applicable for a very large set of regulating TFs in the current implementation. Nevertheless, our results demonstrate that the inclusion of additional domain knowledge or prior assumptions, where available, can improve performance over more general methods. Probabilistic modelling provides a useful framework for the inclusion of such prior knowledge.

**Table 7:** mRNA degradation rates

**mRNA degrad. rates**	**Protein degrad. rates**
**(5%,median,95%)**	**(ANT, BEE, CAR, UNK)**
(0.123, 0.610, 4.807)	(0.994, 0.945, 0.640, 1.200)

Median value (with 5% and 95% percentiles) across all mRNA degradation rates of the 1030 artificially generated genes. The exact values for the protein degradation rates used to simulate the data are also given.

Inference of continuous-time TF activity profiles from short time-series is an ill-posed problem. We resolve this through introduction of a Gaussian process prior that effectively assumes smoothness of the underlying functions [21]. While this assumption appears reasonable for the TFs studied here, there are situations where the TF is activated very rapidly through signalling, e.g. in a sensory GRN [39]. In these situations an alternative model better suited for fast transitions such as that presented in [40] may be preferable. Alternatively, the Gaussian process could be transformed to provide a sharper switching behaviour by passing it through a sigmoidal non-linearity (cf. Gaussian process classification [41]) and the current inference methodology would remain applicable.

Carrying out Bayesian inference over non-linear systems with functional parameters is very challenging. For parameter inference we have made use of state-of-the-art methods for Markov chain Monte Carlo (MCMC) over functional degrees of freedom [42]. We have developed a novel fast method for calculating the Bayesian evidence score that allows us to carry out genome-wide model scoring (see supplementary information). Our method is very easily parallelizable within the prediction phase and can therefore be considered a practical contribution to the functional genomics toolkit.

The data used here are very limited and therefore one must accept that the method will make many false predictions. To improve accuracy, predictions based on the analysis of expression data can be combined with evidence from complementary sources (ChIP data, in situ expression data, sequence motifs) to identify a confident regulatory network structure. For example, [20] show how the accuracy of model-based prediction improves greatly when additional evidence from spatial expression data is considered. The Bayesian framework presented here provides a very natural means for integrating other sources of data or prior knowledge for network inference. For example, it would be straightforward to associate alternative regulatory structures (e.g. those in Figure 2(b)) with different prior probabilities derived from ChIP-chip binding patterns. These priors could be used to re-weight the Bayesian model averaging scheme used to calculate the probability of network structures. We do not pursue this approach here because we want independent ChIP-chip validation of our method’s performance. Alternatively, given time-series ChIP data, one could include binding observations directly in the model. This would have the advantage that one could model measurement errors for both the expression and ChIP experiments.

## Conclusion

We have introduced a computational approach for genome-wide inference of the targets of multiple regulating TFs given time-series gene expression data. Using a time course measuring changes in wild-type expression during the embryonic development of *Drosophila* we were able to show that the method makes predictions which are significantly enriched for TF and TF-pair binding identified using ChIP-chip experiments on the same system. Our method works by fitting and scoring differential equation models of transcriptional regulation. Initially we use the model to infer the temporal pattern of TF protein activity given a small subnetwork of mostly known structure. Subsequently we score alternative target gene regulation models to make genome-wide target predictions. By using a fully Bayesian procedure we are able to automatically balance model complexity with data fit when scoring alternative models. Our method is readily parallelizable in the prediction phase, making it a practical tool for genome-wide network inference. On artificial data we showed that our method is able to cope with the existence of unknown regulating TFs that are not modelled and we showed that data from more diverse experimental conditions can help disambiguate between TFs that have similar profiles in a single condition. However, as our *Drosophila* example shows, even a single wild-type time course can be highly informative about the underlying regulatory network if the TFs of interest are changing over time. By combining the model predictions with other independent sources of evidence, e.g. from ChIP and spatial expression patterns, it will be possible to identify a confident condition-specific regulatory network.

### Availability

Software and a web-based browser displaying results in the *Drosophila* experiment are both available online at [34].

## Methods

### Dynamical models

The transcription and translation equations are ordinary differential equations (ODEs) having the general form given in the beginning of the Results section. The response function *G*(·) non-linearly transforms the TF protein activities ${\left\{p_{i}(t)\right\}}_{i=1}^{I}$, and has the following sigmoidal form:

(3) $$G(p_{1}(t),\ldots ,p_{I}(t);w_{j},w_{j0})=\frac{1}{1+e^{-w_{j0}-\overset{I}{\underset{i=1}{\sum }}w_{\text{ji}}\text{log}p_{i}(t)}}.$$

Here, the *I*-dimensional real-valued vector $w_{j}={\left[w_{j1}\ldots w_{\text{ji}}\right]}^{⊤}$ stores the interaction weights between the *j*^th^ target gene and the *I* TFs. These interaction weights quantify the network links so that when *w*_*ji *_= 0 the link between the *j*^th^ gene and the *i*^th^ TF is absent. When *w*_*ji *_is negative or positive the TF acts as a repressor or activator respectively. *w*_*j*0_ is a real-valued bias parameter. The set of scalar parameters ***θ***_*j*_ in the response function *G*(·) is defined to be ***θ***_*j *_= {**w**_*j*_*w*,_*j*0_}. Since the transcription ODE model is linear with respect to *m*_*j*_(*t*), it can be solved explicitly as shown in the supplementary information. The above transcription ODE model generalizes previous single-TF models that were used to estimate the concentration function of a single latent TF [18,21,43]. While a sigmoidal form for the response function *G*(·) was considered in all our experiments, our algorithms could easily be adapted to handle different forms for *G*(·).

Furthermore, the simple linear translation equation can be solved explicitly as shown in the supplementary information. Finally, the parameters {***θ***_*j*_,*d*_*j*_,*b*_*j*_,*s*_*j*_,*δ*_*i*_} are model parameters in the ODEs which need to be estimated under the constraint that {*d*_*j*_,*b*_*j*_,*s*_*j*_,*δ*_*i*_} attain non-negative real values, while ***θ***_*j *_= {**w**_*j*_,*w*_*j*0_} can attain both positive and negative real values. When we search for TFs that act only as activators, **w**_*j*_ is constrained to be non-negative.

A more detailed description of the ODE models is given in section 2 of the supplementary information.

### Training modelling phase

The dynamical models contain a set of unknown quantities: the transcription model parameters ${\left\{\theta_{j},d_{j},b_{j},s_{j}\right\}}_{j=1}^{J}$, where *J* is the number of target genes, the unobserved TF protein activities ${\left\{p_{i}(t)\right\}}_{i=1}^{I}$ and the TF protein degradation rates ${\left\{\delta_{i}\right\}}_{i=1}^{I}$. To estimate these quantities in the training modelling phase we consider a Bayesian probabilistic approach. More precisely, the observed mRNA data are used to construct likelihood functions that explain how the data are generated from the dynamical models. Together with the mRNA data for each training gene *j* we also have a binary vector $x_{j}\in {\left\{0,1\right\}}^{I}$ that specifies the regulatory network structure for that gene so that *x*_*ji *_= 1 indicates the presence of the link between the gene and the *i* TF, while *x*_*ji *_= 0 indicates the absence of the link. Prior distributions are assigned to all unknown quantities. The prior over each protein activity *p*_*i*_(*t*) was defined through the translation ODE and the placement of a suitable prior on the TF mRNA function, *f*_*i*_(*t*), through the use of Gaussian processes; see e.g. [41]. Bayesian inference in the training modelling phase was performed by Markov chain Monte Carlo (MCMC) techniques [44] where all the above unknown quantities were inferred using suitable MCMC updates.

A more detailed description of the training modelling phase is given in section 3 of the supplementary information.

### Prediction modelling phase

The prediction phase involves independently processing each test gene and probabilistically predicting its regulating TFs. Let ∗ denote a test gene so that **y**_∗_ is the associated vector of observed mRNA measurements. This gene can be regulated by any combination of *I* TFs. Let $x_{*}\in {\left\{0,1\right\}}^{I}$ be the binary vector that indicates the subset of the TFs that regulate gene ∗ which takes 2^*I*^ possible values. To infer the network links, it suffices to compute the posterior probability for each value of the discrete random variable **x**_∗_. Using Bayes’ rule this probability is

(4) $$p(x_{*}|{\text{y}}_{*},\text{Y})=\frac{p({\text{y}}_{*}|x_{*},\text{Y})p(x_{*}|\text{Y})}{\underset{x}{\sum }p({\text{y}}_{*}|x,\text{Y})p(x|\text{Y})},$$

where **Y**indicates the data used in the training modelling phase. To obtain the above, we need to compute the predictive density *p*(**y**_∗_|**x**_∗_**Y**) for any possible combination of regulating TFs, i.e. any value of **x**_∗_, together with the associated probabilities *p*(**x**_∗_|**Y**). While *p*(**x**_∗_|**Y**) could be computed by the frequencies of the known connectivity vectors in the training genes, this is unreliable since the small set of training genes may not be representative about the prior distribution of links between TFs and genes. Therefore, we set these probabilities to uniform values so that the posterior probability in Equation (1) becomes proportional to its predictive density value *p*(**y**_∗_|**x**_∗_**Y**). This latter quantity is intractable since it requires an integration over the parameters (***θ***_∗_,*d*_∗_,*b*_∗_,*s*_∗_). We approximate it using a novel fast approximation to a marginal likelihood, described in detail in section 4.1 in the supplementary information, that follows ideas similar to Chib’s approximation [45].

Given the estimated probabilities *p*(**x**_∗_|**y**_∗_,**Y**), with $x_{*}\in {\left\{0,1\right\}}^{I}$, any query related to the regulating TFs of target gene ∗ can be answered. For instance, in the results we made use of the following quantities: 

· Maximum a posteriori (MAP) network configuration: This is the most probable setting $x_{*}^{\text{MAP}}$ for the network links obtained by

(5) $$x_{*}^{\text{MAP}}=\text{arg}\;\underset{x_{*}}{\text{max}}\;p(x_{*}|{\text{y}}_{*},\text{Y}).$$

· Marginal probability of a single link: The link between the test gene and the *i*^th^ TF is present with posterior probability

(6) $$p(x_{*i}=1|{\text{y}}_{*},\text{Y})=\underset{x_{*}:x_{*i}=1}{\sum }p(x_{*}|{\text{y}}_{*},\text{Y}).$$

Similarly we can compute the marginal probability *p*(*x*_∗*i*_ = 1,*x*_∗*j*_ = 1|**y**_∗_,**Y**) for a pair of links.

A more detailed description of the prediction modelling phase is given in section 4 of the Supplementary Information.

### The “Maximum Likelihood Baseline” method

This method, that was used in the experiments in *Drosophila*, follows exactly the same structure as the Bayesian approach with the following two differences. Firstly, the model parameters (such as kinetic parameters in the ODEs) were not treated using a Bayesian manner and instead they were obtained based on maximum likelihood which provides point estimates. Secondly, each protein function, *p*_*i*_(*t*), was deterministically estimated by the translation ODE model and by setting the driving TF mRNA function, *f*_*i*_(*t*), to a piece-wise linear interpolation function computed from the TF mRNA observations. Apart from the above differences, prediction using the baseline method is done exactly analogously to the Bayesian case.

### The “Regression” method

In the experiments in *Drosophila* (Figure 7), we made use of a simple method for predicting the regulators of a target gene based on linear regression that predicts the mRNA of target gene from the TF mRNA. In particular, for a target gene *j* this linear model is

(7) $$m_{\text{jn}}=\overset{I}{\underset{i=1}{\sum }}w_{\text{ji}}f_{\text{in}}+w_{j0}+\varepsilon_{n},\;\forall n,$$

where *m*_*jn*_ is the observed mRNA of the target gene at time *t*_*n*_${\left\{f_{\text{in}}\right\}}_{i=1}^{I}$ the corresponding observed TF mRNA values, $\left({\left\{w_{\text{ji}}\right\}}_{j=1}^{I},w_{j0}\right)$ are parameters to be inferred and *ε*_*n*_ is Gaussian noise. Notice that, ${\left\{w_{\text{ji}}\right\}}_{j=1}^{I}$ are interaction weights and *w*_*j*0_ is a bias parameter. Network inference in this linear model reduces to finding the non-zero interaction weights. This problem would typically require sparse optimization methods based on *ℓ*_1_ regularization as considered in [46]. However, in our case such algorithms are not needed since the number of TFs is small (*I *= 5) and hence we can enumerate all possible 32 regression models and select the best model using cross-validation. In the results reported in Figure 7, we firstly computed for each gene the MSE scores on held-out data (using 12-fold cross validation) for all 32 models. Subsequently, we selected the model with the smallest MSE score for each gene and finally we ranked all genes based on the latter MSE scores (in ascending order) to produce the rankings shown in Figure 7.

### Application of the Inferelator 1.1

We compared our method against Inferelator 1.1 [15] which is available for download at http://err.bio.nyu.edu/inferelator/. This is the most recent version for which source code is available and which can be easily used for new data. We set each gene in its own cluster but otherwise used the default settings. We interpreted the maximum of the absolute values |*β*_*i*_| of all weights corresponding to a specific regulator alone or in combination with another as the counterpart of the posterior probability for ranking the predictions. For pairs, the corresponding value was $\text{max}(|\beta_{3}|,\min(|\beta_{1}|,|\beta_{2}|))$, where *β*_1_ and *β*_2_ are the weights of the components (*x*_1_*x*_2_) of the pair and *β*_3_ is the weight of $\min(x_{1},x_{2})$ (see Eq. (6) in [15]). Combining information from independent and interaction terms like this significantly increased the performance of the method. Ranking by |*β*_*i*_| was also used in DREAM3 challenge submission of the Inferelator team [47].

### Preprocessing of the *Drosophila* data

As previously described [20].

### Training set for the *Drosophila* data

The training set was constructed from the training set of 310 ChIP *cis*-regulatory modules (CRMs) collected in [12] (Additional file 1: Table S8). The modules were mapped to genes using the CRM activity database in [12] (Additional file 1: Table S4). Multiple CRMs for a gene were combined by taking the union of detected binding. Weakly expressed genes as defined in [20] were excluded, leaving a training set of 92 genes with well-characterised TF binding profiles.

### Bootstrap significance testing of ranking method performance differences

100,000-fold bootstrap resampling was used to assess statistical significance of performance differences between different ranking methods. For each fold, the set of testing genes was resampled with replacement from the full set of 6003 genes. Top-ranked predictions within the resampled set were evaluated as usual and the fraction of folds where each method outperformed each other was tabulated.

### Reduced training set for the *Drosophila* data using a robustified model

Since in the *Drosophila* data the target genes can be influenced by unknown factors that are not part of the model, we considered a robustified training procedure that filtered out genes not explained by the model. This procedure allowed us to reduce the initial set of 92 genes to 25 genes and was carried out as follows. Firstly, we performed a training phase using all 92 genes so that the likelihood functions had both preprocessing noise variances and additive gene-specific adaptive variances. Then, genes having large inferred adaptive variances, which indicates that these genes cannot be explained well by the five-TF model, are excluded so that finally a subset of 25 genes was retained. Then, the whole training phase was repeated using only the selected genes and without the additive variances this time. The selection involved setting a threshold, which was set to 0.01, so that genes having estimated adaptive variance larger than this threshold were excluded. The threshold value was chosen to be smaller than the average value of the preprocessing variances, which represent estimates of the actual observation noise in the gene expression measurements.

Robust fitting was also used in the prediction phase so that each test gene was fitted using a likelihood function in which the variance parameter was the sum of a fixed preprocessing noise variance and an adaptive variance. Again this allowed us to compensate for the model mismatch and the presence of other confounding factors which, while they could regulate the gene expression, are not part of the model. More details on the robustified fitting are given in Section 3 and 5.2 of the Supplementary Material.

### DroID validation

We downloaded the TF-gene interaction database from DroID ( http://www.droidb.org, release 2011_11). Genes with no interactions in the database were excluded from the validation to avoid possible problems due to annotation incompatibilities.

### Generation of the synthetic data

We generated synthetic mRNA time-series data that correspond to 1030 target genes and four transcription factors: ANT, BEE, CAR and UNK. The TF activities are depicted in the first column of Figure 3(c). For both experimental conditions, the TF activities have been generated by simulating the translation ODE equation by assuming certain profiles for the TF mRNA functions, ${\left\{f_{i}(t)\right\}}_{i=1}^{4}$, which were chosen to have the profiles shown in Additional file 1: Figure S1, and with protein degradation rates 0.994, 0.945, 0.640, 1.2 for the four TFs respectively. ANT, BEE and CAR are assumed to be known factors for which observations of their TF mRNA activities are available. UNK is assumed to be a confounding factor whose presence and origin is not known. Given these TF mRNA functions, ${\left\{f_{i}(t)\right\}}_{i=1}^{3}$, noisy “observations” were obtained at ten non-uniformly spaced time points, *t*_*k *_∈ {0,1,2,3,5,7,9,11,14,18}, by adding zero-mean Gaussian noise with variance 0.025 *f *_*i*_(*t*_*k*_) to the value *f *_*i*_(*t*_*k*_). Negative values were truncated to zero.

To generate mRNA observations for the target genes, we simulated the transcription ODE, given the known TF activities and by using model parameters (***θ***_*j*_,*d*_*j*_,*b*_*j*_,*s*_*j*_) selected as follows. Each interaction weight *w*_*ji*_ for the TFs ANT, BEE and CAR was selected from the distribution $0.5N(0.5,1)+0.5\delta_{0}$ which with 0.5 probability sets the interaction weight to zero and with equal probability selects a value drawn from a Gaussian distribution with mean 0.5 and unit variance. The interaction weight for UNK was selected from $0.25N(0.5,1)+0.75\delta_{0}$. Notice that when *w*_*ji *_= 0, the *i*^th^ TF does not regulate the *j*^th^ gene. The above procedure generates random sets of regulating TFs so that on average each target gene has approximately two regulating TFs. Each bias parameter *w*_*j*0_ was drawn from the Gaussian N(0,1). The kinetic parameters (*d*_*j*_,*b*_*j*_,*s*_*j*_) plus an initial condition parameter *a*_*j*_ (see supplementary information) were selected randomly from an empirical distribution obtained by applying the dynamical models to the 6095 genes (the 92 training genes plus the 6003 test genes) in the *Drosophila* data. This was done to obtain kinetic parameters that produce realistic mRNA profiles that closely resemble real gene expression data. Summaries of the values of these parameters are given in Table 7. Given the above simulated mRNA functions the observations are obtained at the ten non-uniformly spaced time points, mentioned earlier, by adding zero-mean Gaussian noise with variance 0.025 *m*_*j*_(*t*_*k*_) to the value *m*_*j*_(*t*_*k*_). Negative values were truncated to zero.

## Abbreviations

ChIP: Chromatin immunoprecipitation; CRM: cis-regulatory module; DREAM: Dialogue for Reverse Engineering Assessments and Methods; GRN: Gene Regulatory Network; MAP: Maximum a posteriori; MCMC: Markov chain Monte Carlo; MSE: Mean squared error; ODE: Ordinary differential equation; ROC: Receiver operating characteristic; TF: Transcription factor.

## Competing interests

The authors declare that they have no competing interests.

## Author’s contributions

MT developed the MCMC methodology and performed the simulations together with AH. AH and MT developed the validation method. MT, AH, NL and MR designed the MCMC method. MT, AH, ML and MR were involved in drafting the manuscript. All authors read and approved its final version.

## Supplementary Material

Additional file 1 **Supplementary Information. More detailed technical description of the methods and supplementary figures **[4,20,21,26,33,39,42,44,45,48-65]**.** Click here for file

Additional file 2 Posterior probabilities of alternative regulation models for Drosophila. Click here for file

## References

[B1] Bansal M Belcastro V Ambesi-Impiombato A di Bernardo D How to infer gene networks from expression profiles Mol Syst Biol 2007 3 78 [ http://dx.doi.org/10.1038/msb4100120] 17299415 10.1038/msb4100120 PMC1828749

[B2] Wang RS Zhang XS Chen L Inferring transcriptional interactions and regulator activities from experimental data Mol Cells 2007 24 3 307 315 18182844

[B3] De Smet R Marchal K Advantages and limitations of current network inference methods Nat Rev Microbiol 2010 8 10 717 729 [ http://dx. doi.org/10.1038/nrmicro2419] 20805835 10.1038/nrmicro2419

[B4] Marbach D Prill RJ Schaffter T Mattiussi C Floreano D Stolovitzky G Revealing strengths and weaknesses of methods for gene network inference Proc Natl Acad Sci USA 2010 107 14 6286 6291 [ http://dx.doi. org/10.1073/pnas.0913357107] 10.1073/pnas.0913357107 20308593 PMC2851985

[B5] Penfold C Wild D How to infer gene networks from expression profiles, revisited Interface Focus 2011 1 6 857 870 10.1098/rsfs.2011.0053 23226586 PMC3262295

[B6] DeRisi JL Iyer VR Brown PO Exploring the metabolic and genetic control of gene expression on a genomic scale Science 1997 278 5338 680 686 10.1126/science.278.5338.680 9381177

[B7] Chua G Robinson MD Morris Q Hughes TR Transcriptional networks: reverse-engineering gene regulation on a global scale Curr Opin Microbiol 2004 7 6 638 646 [ http://dx.doi.org/10.1016/j.mib.2004.10. 009] 10.1016/j.mib.2004.10.009 15556037

[B8] Chua G Morris QD Sopko R Robinson MD Ryan O Chan ET Frey BJ Andrews BJ Boone C Hughes TR Identifying transcription factor functions and targets by phenotypic activation Proc Natl Acad Sci USA 2006 103 32 12045 12050 [ http://dx.doi.org/10.1073/pnas. 0605140103] 10.1073/pnas.0605140103 16880382 PMC1567694

[B9] Ren B Robert F Wyrick JJ Aparicio O Jennings EG Simon I Zeitlinger J Schreiber J Hannett N Kanin E Volkert TL Wilson CJ Bell SP Young RA Genome-wide location and function of DNA binding proteins Science 2000 290 5500 2306 2309 [ http://dx.doi.org/10.1126/science. 290.5500.2306] 10.1126/science.290.5500.2306 11125145

[B10] Harbison CT Gordon DB Lee TI Rinaldi NJ Macisaac KD Danford TW Hannett NM Tagne JB Reynolds DB Yoo J Jennings EG Zeitlinger J Pokholok DK Kellis M Rolfe PA Takusagawa KT Lander ES Gifford DK Fraenkel E Young RA Transcriptional regulatory code of a eukaryotic genome Nature 2004 431 7004 99 104 [ http://dx.doi.org/10.1038/ nature02800] 10.1038/nature02800 15343339 PMC3006441

[B11] MacQuarrie KL Fong AP Morse RH Tapscott SJ Genome-wide transcription factor binding: beyond direct target regulation Trends Genet 2011 27 4 141 148 [ http://dx.doi.org/10.1016/j.tig.2011.01.001] 10.1016/j.tig.2011.01.001 21295369 PMC3068217

[B12] Zinzen RP Girardot C Gagneur J Braun M Furlong EEM Combinatorial binding predicts spatio-temporal cis-regulatory activity Nature 2009 462 7269 65 70 [ http://dx.doi.org/10.1038/nature08531] 10.1038/nature08531 19890324

[B13] Beal MJ Falciani F Ghahramani Z Rangel C Wild DL A Bayesian approach to reconstructing genetic regulatory networks with hidden factors Bioinformatics 2005 21 3 349 356 [ http://dx.doi.org/10. 1093/bioinformatics/bti014] 10.1093/bioinformatics/bti014 15353451

[B14] Werhli AV Grzegorczyk M Husmeier D Comparative evaluation of reverse engineering gene regulatory networks with relevance networks, graphical Gaussian models and Bayesian networks Bioinformatics 2006 22 20 2523 2531 [ http://dx.doi.org/10.1093/ bioinformatics/btl391] 10.1093/bioinformatics/btl391 16844710

[B15] Bonneau R Reiss DJ Shannon P Facciotti M Hood L Baliga NS Thorsson V The Inferelator: an algorithm for learning parsimonious regulatory networks from systems-biology data sets de novo Genome Biol 2006 7 5 R36 [ http://dx.doi.org/10.1186/gb-2006-7-5-r36] 10.1186/gb-2006-7-5-r36 16686963 PMC1779511

[B16] Bansal M di Bernardo D Inference of gene networks from temporal gene expression profiles IET Syst Biol 2007 1 5 306 312 10.1049/iet-syb:20060079 17907680

[B17] Aijö T Lähdesmäki H Learning gene regulatory networks from gene expression measurements using non-parametric molecular kinetics Bioinformatics 2009 25 22 2937 2944 [ http://dx.doi.org/10.1093/ bioinformatics/btp511] 10.1093/bioinformatics/btp511 19706742

[B18] Barenco M Tomescu D Brewer D Callard R Stark J Hubank M Ranked prediction of p53 targets using hidden variable dynamic modeling Genome Biol 2006 7 3 R25 10.1186/gb-2006-7-3-r25 16584535 PMC1557743

[B19] Della Gatta G Bansal M Ambesi-Impiombato A Antonini D Missero C di Bernardo D Direct targets of the TRP63 transcription factor revealed by a combination of gene expression profiling and reverse engineering Genome Res 2008 18 6 939 948 [ http://dx.doi.org/10. 1101/gr.073601.107] 10.1101/gr.073601.107 18441228 PMC2413161

[B20] Honkela A Girardot C Gustafson EH Liu YH Furlong EEM Lawrence ND Rattray M Model-based method for transcription factor target identification with limited data Proc Natl Acad Sci USA 2010 107 17 7793 7798 10.1073/pnas.0914285107 20385836 PMC2867914

[B21] Gao P Honkela A Rattray M Lawrence ND Gaussian process modelling of latent chemical species: applications to inferring transcription factor Activities Bioinformatics 2008 24 16 i70 i75 10.1093/bioinformatics/btn278 18689843

[B22] Honkela A Gao P Ropponen J Rattray M Lawrence ND tigre: Transcription factor inference through Gaussian process reconstruction of expression for Bioconductor Bioinformatics 2011 27 7 1026 1027 [ http://dx.doi.org/10.1093/bioinformatics/btr057] 10.1093/bioinformatics/btr057 21300702

[B23] Madar A Greenfield A Ostrer H Vanden-Eijnden E Bonneau R The Inferelator 2.0: a scalable framework for reconstruction of dynamic regulatory network models Proceedings of the Annual International Conference of the IEEE Engineering in Medicine and Biology Society 2009 5448 5451 10.1109/IEMBS.2009.5334018 19964678

[B24] Prill RJ Marbach D Saez-Rodriguez J Sorger PK Alexopoulos LG Xue X Clarke ND Altan-Bonnet G Stolovitzky G Towards a rigorous assessment of systems biology models: the DREAM3 challenges PLoS One 2010 5 2 e9202 [ http://dx.doi.org/10.1371/journal.pone. 0009202] 10.1371/journal.pone.0009202 20186320 PMC2826397

[B25] Greenfield A Madar A Ostrer H Bonneau R DREAM4: Combining genetic and dynamic information to identify biological networks and dynamical models PLoS One 2010 5 10 e13397 [ http://dx.doi.org/ 10.1371/journal.pone.0013397] 10.1371/journal.pone.0013397 21049040 PMC2963605

[B26] Gelman A Carlin J Stern H Rubin D Bayesian Data Analysis 2003 Boca Raton: Chapman and Hall/CRC

[B27] Veitia RA A sigmoidal transcriptional response: cooperativity, synergy and dosage effects Biol Rev Camb Philos Soc 2003 78 149 170 10.1017/S1464793102006036 12620064

[B28] Black BL Olson EN Transcriptional control of muscle development by myocyte enhancer factor-2 (MEF2) proteins Annu Rev Cell Dev Biol 1998 14 167 196 [ http://dx.doi.org/10.1146/annurev.cellbio.14.1.167] 10.1146/annurev.cellbio.14.1.167 9891782

[B29] Molkentin JD Black BL Martin JF Olson EN Mutational analysis of the DNA binding, dimerization, and transcriptional activation domains of MEF2C Mol Cell Biol 1996 16 6 2627 2636 8649370 10.1128/mcb.16.6.2627 PMC231253

[B30] Castanon I Stetina SV Kass J Baylies MK Dimerization partners determine the activity of the Twist bHLH protein during Drosophila mesoderm development Development 2001 128 16 3145 3159 11688563 10.1242/dev.128.16.3145

[B31] Zaffran S Frasch M The beta 3 tubulin gene is a direct target of bagpipe and biniou in the visceral mesoderm of Drosophila Mech Dev 2002 114 1-2 85 93 10.1016/S0925-4773(02)00063-1 12175492

[B32] Zaffran S Frasch M The homeodomain of Tinman mediates homo- and heterodimerization of NK proteins Biochem Biophys Res Commun 2005 334 2 361 369 [ http://dx.doi.org/10.1016/j.bbrc.2005.06.090] 10.1016/j.bbrc.2005.06.090 16004970

[B33] Tomancak P Beaton A Weiszmann R Kwan E Shu S Lewis SE Richards S Ashburner M Hartenstein V Celniker SE Rubin GM Systematic determination of patterns of gene expression during Drosophila embryogenesis Genome Biol 2002 3 12 RESEARCH0088 12537577 10.1186/gb-2002-3-12-research0088 PMC151190

[B34] Modelling software and a browser of Drosophila results [ http://www.bioinf.manchester.ac.uk/resources/tiger/multitf/]

[B35] Murali T Pacifico S Yu J Guest S Roberts GG Finley RL DroID 2011: a comprehensive, integrated resource for protein, transcription factor, RNA and gene interactions for Drosophila Nucleic Acids Res 2011 39 Database issue D736 D743 [ http://dx.doi.org/10.1093/nar/ gkq1092] 21036869 10.1093/nar/gkq1092 PMC3013689

[B36] Su YW Xie TX Sano D Myers JN IL-6 stabilizes Twist and enhances tumor cell motility in head and neck cancer cells through activation of casein kinase 2 PLoS One 2011 6 4 e19412 [ http://dx.doi.org/10. 1371/journal.pone.0019412] 10.1371/journal.pone.0019412 21559372 PMC3084854

[B37] Stolovitzky G Monroe D Califano A Dialogue on reverse-engineering assessment and methods: the DREAM of high-throughput pathway inference Ann N Y Acad Sci 2007 1115 1 22 [ http://dx.doi.org/10.1196/ annals.1407.021] 10.1196/annals.1407.021 17925349

[B38] Barenco M Brewer D Papouli E Tomescu D Callard R Stark J Hubank M Dissection of a complex transcriptional response using genome-wide transcriptional modelling Mol Syst Biol 2009 5 327 [ http://dx.doi.org/10.1038/msb.2009.84] 19920812 10.1038/msb.2009.84 PMC2795478

[B39] Alon U An Introduction to Systems Biology: Design Principles of Biological Circuits 2006 London: Chapman and Hall/CRC

[B40] Sanguinetti G Ruttor A Opper M Archambeau C Switching regulatory models of cellular stress response Bioinformatics 2009 25 10 1280 1286 [ http://dx.doi.org/10.1093/bioinformatics/btp138] 10.1093/bioinformatics/btp138 19279066

[B41] Rasmussen CE Williams CKI Gaussian Processes for Machine Learning 2006 MA: MIT Press, Cambridge

[B42] Titsias MK Lawrence ND Rattray M Koller D, Schuurmans D, Bengio Y, Bottou L Efficient Sampling for Gaussian Process Inference using Control Variables Advances in Neural Information Processing Systems 21 2009 1681 1688

[B43] Rogers S Khanin R Girolami M Bayesian model-based inference of transcription factor activity BMC Bioinformatics 2007 8 Suppl 2 S2 10.1186/1471-2105-8-S2-S2 17493251 PMC1892071

[B44] Robert CP Casella G Monte Carlo Statistical Methods 2004 New York: Springer-Verlag

[B45] Chib S Marginal Likelihood from the Gibbs Output J Roy Stat Soc B 1995 90 432 1313 1321

[B46] van Someren EP Vaes BLT Steegenga WT Sijbers AM Dechering KJ Reinders MJT Least absolute regression network analysis of the murine osteoblast differentiation network Bioinformatics 2006 22 4 477 484 10.1093/bioinformatics/bti816 16332709

[B47] Madar A Greenfield A Vanden-Eijnden E Bonneau R DREAM3: network inference using dynamic context likelihood of relatedness and the inferelator PLoS One 2010 5 3 e9803 [ http://dx.doi.org/10.1371/ journal.pone.0009803] 10.1371/journal.pone.0009803 20339551 PMC2842436

[B48] Andrieu C Thoms J A tutorial on adaptive MCMC Stat Comput 2008 18 343 373 10.1007/s11222-008-9110-y

[B49] Calderhead B Girolami MA Estimating Bayes factors via thermodynamic integration and population MCMC Comput Stat Data An 2009 53 12 4028 4045 10.1016/j.csda.2009.07.025

[B50] Christensen OF Roberts GO Sköld Sköld Robust Markov chain Monte carlo methods for spatial generalized linear mixed models J Comput Graph Stat 2006 15 1 17 10.1198/106186006X100470

[B51] Earl DJ Deem MW Parallel tempering: Theory, applications, and new perspectives Phys Chem Chem Phys 2005 7 23 3910 3916 19810318 10.1039/b509983h

[B52] Friel N Pettitt AN Marginal likelihood estimation via power posteriors J Roy Stat Soc B 2008 70 3 589 607 10.1111/j.1467-9868.2007.00650.x

[B53] Gelman A Meng XL Simulating normalizing constants: from importance sampling to bridge sampling to path sampling Stat Sci 1998 13 2 163 185

[B54] Gelman A Roberts GO Gilks WR Bernardo JM, Berger JO, David AF, Smith AFM Efficient Metropolis jumping rules Bayesian Statistics. Volume 5 1996 Oxford: Oxford University Press 599 607

[B55] Gelman A Rubin DB Inference from iterative simulation using multiple sequences Stat Sci 1992 7 4 457 472 10.1214/ss/1177011136

[B56] Geyer CJ Practical Markov Chain Monte Carlo Stat Sci 1992 7 4 473 483 10.1214/ss/1177011137

[B57] Girolami M Calderhead B Riemann manifold Langevin and Hamiltonian Monte Carlo methods J Roy Stat Soc B 2011 73 2 123 214 10.1111/j.1467-9868.2010.00765.x

[B58] Kuss M Rasmussen CE Assessing approximate inference for binary Gaussian process classification J Mach Learn Res 2005 6 1679 1704

[B59] Murray I Adams RP Lafferty J, Williams CKI, Shawe-Taylor J, Zemel RS, Culotta A Slice sampling covariance hyperparameters of latent Gaussian models Advances in, Neural Information Processing Systems 23 2010 1732 1740

[B60] Murray I Adams RP MacKay DJ Elliptical slice sampling JMLR: W&CP 2010 9 541 548

[B61] Neal RM Annealed importance sampling Stat Comput 1998 11 125 139

[B62] Newton MA Raftery AE Weighted likelihood bootstrap J Roy Stat Soc B 1994 56 3 48

[B63] Pearson RD Liu X Sanguinetti G Milo M Lawrence ND Rattray M Puma: a Bioconductor package for propagating uncertainty in microarray analysis BMC Bioinf 2009 10 211 10.1186/1471-2105-10-211 PMC2714555 19589155

[B64] Roberts GO Gelman A Gilks WR Weak convergence and optimal scaling of random walk Metropolis algorithms Ann Appl Probab 1996 7 110 120

[B65] Schmidt MN Function factorization using warped Gaussian processes International Conference on Machine Learning 26 2009 921 928

