# Supplementary material for: Identifying targets of multiple co-regulating transcription factors from expression time-series by Bayesian model comparison
Source: BMC Syst Biol. 2012 May 30;6:53. doi: 10.1186/1752-0509-6-53 (PMC3527261; doi:10.1186/1752-0509-6-53)
Supplement: Additional file 1 — Supplementary Information. More detailed technical description of the methods and supplementary figures [4,20,21,26,33,39,42,44,45,48-65]. [file 1752-0509-6-53-S1.pdf]

Identifying targets of multiple co-regulating transcription factors from expression time-series by Bayesian model comparison (Supplementary information)

Abstract

This technical report serves as supplementary material for the submission "Identifying targets of multiple co-regulating transcription factors from expression time-series by Bayesian model comparison". It describes all the technical details of all used methods and algorithms.

1 Structure

The technical report is divided into three main sections. Section 2 describes the dynamical models for gene transcription that assume joint regulation of genes by multiple transcription factors (TFs). Section 3 presents the Bayesian methodology for learning the profiles of the TFs by using a small set of target genes with known connectivity as the training data. Section 4 applies the model to genome-wide prediction of the network connections from the TFs to test genes, i.e. genes for which the combination of regulating TFs is unknown. The two latter sections describe the training and prediction phases respectively defined in the main article. Finally, Section 5 provides supplemental plots for the experimental results presented in the main paper. For completeness, we start our discussion by repeating the definitions of the training and prediction modelling phases:

1. Training phase (estimation of the TF activity profiles): Here, we use the dynamical models to estimate the TF activities, $p_i(t)$, by using a small set of *training* genes. The structure of the sub-network is assumed to be given so that for these genes the identities of regulating TFs are known, although the model is able to exclude inactive relationships. In this phase both the transcription model and optionally a TF protein translation model are used to estimate the TF activities. This requires observations associated with the mRNA of the training genes as well as optionally the TF mRNAs if the translation model is used.

2. Prediction phase: Once the TF activities have been estimated, each *test* gene (for which the combination of regulating TFs are unknown) is processed independently and the model parameters are inferred. This step is based on the estimated TF activities and hence only the transcription model is used. This phase is applied on a genome-wide scale and aims at identifying the regulating TFs for each test gene.

In the remaining part of the report we explain the dynamical models and present all the details about how the above modelling phases are implemented.

2 Multiple transcription factors and ODE models for transcription and translation

We model the transcriptional regulation by using ordinary differential equations (ODEs) that incorporate the contribution of multiple TFs in regulating each gene. Let $m_j(t)$ be the time-continuous function that represents the mRNA concentration of target gene j at time t and where $j = 1, \ldots, J$ is the set of genes. Let $p_i(t)$, where $i = 1, \ldots, I$, be a time-continuous function that represents the TF protein activity. We model the rate of change in the production of mRNA of each gene by the following ODE

$$\frac{\mathrm{d}m_j(t)}{\mathrm{d}t} = b_j + s_j G(p_1(t), \ldots, p_I(t); \mathbf{w}_j, w_{j0}) - d_j m_j(t). \tag{1}$$

Here, b_j represents a constant background production rate, called the basal rate, s_j is a sensitivity parameter and d_j is the mRNA degradation rate. The function $G(\cdot)$ represents a non-linear response that allows the TFs to competitively or co-operatively activate or repress the transcription. A reasonable assumption for this non-linear response is to follow a sigmoidal form (Veitia, 2003), similarly to the sigmoidal and Hill functions used in single input motif models (Alon, 2006). In all our experiments we use

$$G(p_1(t), \ldots, p_I(t); \mathbf{w}_j, w_{j0}) = \frac{1}{1 + e^{-w_{j0} - \sum_{i=1}^{I} w_{ji} \log p_i(t)}}, \tag{2}$$

which is the standard sigmoid function that takes values in $[0, 1]$ and receives as inputs the logarithm of the TF activities. Here, w_{j0} is a real-valued bias parameter and the I-dimensional real-valued vector $\mathbf{w}_j = [w_{j1} \ldots w_{jI}]^{\mathrm{T}}$ represents the interaction weights between the jth target gene and the I TFs. These parameters quantify the strength of the network links between TFs and genes in the underlying regulatory network. Specifically, when $w_{ji} = 0$ the link between the jth gene and the ith TF is absent while when w_{ji} is negative or positive the TF acts as a repressor or activator respectively. Notice that by estimating the values of the interaction weights, we can infer the network links between TFs and target genes. An alternative way of writing Eq. (2) is as

$$G(p_1(t), \ldots, p_I(t); \mathbf{w}_j, w_{j0}) = \frac{\prod_{i=1}^{I} p_i(t)^{w_{ji}}}{\gamma_j + \prod_{i=1}^{I} p_i(t)^{w_{ji}}}, \tag{3}$$

which can be thought as a "multiple-TF Hill function" where $\gamma_j = e^{-w_{j0}}$ is a regulation coefficient. The above way of writing the sigmoid function reveals the multiplicative combination of the TFs. It is possible to combine the TFs differently by selecting different forms for $G(\cdot)$. We could, for instance, combine the TFs by addition or both multiplication and addition (see, e.g. Marbach et al., 2010). While all our experiments are based on $G(\cdot)$ having the above sigmoid form, it is straightforward to apply our methodology and use our software implementation to deal with other functional forms of $G(\cdot)$ too.

The ODE (1) is linear with respect to the mRNA function $m_j(t)$. Therefore it can be solved explicitly for $t \geq 0$ to give

$$m_j(t) = \frac{b_j}{d_j} + \left(a_j - \frac{b_j}{d_j}\right) e^{-d_j t} + s_j \int_0^t G(p_i(u), \ldots, p_I(u); \mathbf{w}_j, w_{j0}) e^{-d_j(t-u)} \, \mathrm{d}u, \tag{4}$$

where a_j arises from the initial condition. The parameters $\mathbf{k}_j = (b_j, d_j, s_j, a_j)$ are the kinetic parameters in the ODE which all take non-negative values. The kinetic parameters $\mathbf{k}_j$ together with the interaction weights $\mathbf{w}_j$ and bias w_{j0} are the unknown model parameters that need to be estimated in order to fit the ODE to the

dynamics of a certain biological system. The TF protein activities, $\{p_i(t)\}_{i=1}^I$, are typically unobserved and therefore we need to estimate these functions as well. Our objective is to use observed noisy measurements of the mRNA function $\{m_j(t)\}_{j=1}^J$ to infer the transcription ODE model. Sections 3 and 4 describe in full detail the Bayesian estimation methods used.

When observations of the TF mRNAs are available, the above transcription model can be combined with a protein translation model. This can increase the power for the estimation of the TF profiles by taking advantage of all available mRNA data. Next we describe the TF translation model.

2.1 Dynamic model for the TF translation

We use a dynamic ODE model that explains the change of the protein activity $p_i(t)$ in terms of its mRNA. This follows the work of Honkela et al. (2010). Let $f_i(t)$ be the mRNA concentration function associated with the protein activity $p_i(t)$. We assume these functions are coupled by the following ODE:

$$\frac{\mathrm{d}p_i(t)}{\mathrm{d}t} = f_i(t) - \delta_i p_i(t), \tag{5}$$

where δ_i represents the TF protein degradation rate. This is a simple linear equation with solution

$$p_i(t) = \int_0^t f_i(t) e^{-\delta_i(t-u)}\,\mathrm{d}u, \tag{6}$$

where transient terms associated with the initial conditions are ignored. This dynamical model allows the incorporation of available mRNA measurements for the TF mRNAs $\{f_i(t)\}_{i=1}^I$. The solution in Eq. (6) can be thought of as a parametrised model for the protein activity $p_i(t)$ that depends on the mRNA function and degradation parameter. The translation model allows the use of TF mRNA observed measurements as discussed next.

3 *Training phase:* Learning from mRNA data associated with a small set of genes of known connectivity

In the training modelling phase we assume a small set of J_{tr} genes as the training data. For these genes it is assumed that there exists prior knowledge regarding the network links. Specifically, for each gene j we know the non-regulating TFs, i.e. the TFs for which w_{ji} is fixed to zero. For the remaining TFs the corresponding interaction weights are unknown parameters that need to be inferred. In short, the above prior knowledge simply excludes certain TFs from regulating a gene. We can represent this by a binary vector $\mathbf{x}_j \in \{0,1\}^I$ that indicates the set of candidate regulators for gene j. Given $\mathbf{x}_j$, the mRNA function from Eq. (4) is written as

$$m_j(t) = \frac{b_j}{d_j} + \left(a_j - \frac{b_j}{d_j}\right) e^{-d_j t} + s_j \int_0^t \frac{1}{1 + e^{-w_{j0} - \sum_{i: x_{ji}=1} w_{ji} \log p_i(t)}} e^{-d_j(t-u)}\,\mathrm{d}u, \tag{7}$$

which holds for $j = 1, \ldots, J_{tr}$. In the training modelling phase we also make use of the translation ODE model which is given by Eq. (6).

The main objective of the training phase is to estimate the TF activities $\{p_i(t)\}_{i=1}^I$. To achieve this, we need to apply an inference methodology. Our inference methodology is based on probabilistic Bayesian learning and next we describe in full detail all the steps involved. Section 3.1 defines the probabilistic model

and the prior distributions of all parameters. Section 3.2 discusses observed data and handling of replicate measurements. Section 3.3 discusses the numerical methods for likelihood evaluation. Section 3.4 gives the form of prior distributions of all unknown quantities and Section 3.5 explains the Markov chain Monte Carlo (MCMC) algorithm used to obtain samples from the posterior distribution.

3.1 Probabilistic model

The model parameters together with related mathematical notation are summarised in Table 1. Most of the parameters involve the ODE model and have been already introduced previously. The remaining parameters are defined in the subsequent sections and involve the noise variances in the likelihood function as well as learnable hyperparameters of the prior distributions. The table also includes notation regarding the observed data and other less significant mathematical quantities.

3.2 Data processing and observation model

We assume that there exist noisy observations of the mRNA functions for all J_{tr} training genes from R different experiments (commonly referred to as replicates). We assume conditionally independent time evolution for all experiments given the model parameters. All model parameters are shared except initial condition in case of experiments performed under different conditions. For instance, in the *Drosophila* data set (see main paper) we have three replicas of the same experimental condition. In the artificial data set, we have two replicas associated with two different experimental conditions. For convenience of notation we assume all replicates are measured at the same time points although this is easy to generalise.

Specifically, these observations are evaluated at N_m (possibly non-uniformly spaced) time points $\mathcal{T}_m = \{t_1, \ldots, t_{N_m}\}$ where $t_1 = 0 < t_2 \ldots < t_{N_m}$. The mRNA measurement $\widetilde{m}_{jn}^r$ for the gene j, replica r and at time t_n is generated according to

$$\widetilde{m}_{jn}^r = m_{jn}^r + \epsilon, \quad \epsilon \sim N(0, \sigma_{m,jnr}^2 + \sigma_{m,j}^2), \tag{8}$$

where $m_{jn}^r = m_j^r(t_n)$ is given by Eq. (7) for the rth replicate. $\sigma_{m,jnr}^2$ is a known gene-specific and time-specific noise variance. Such variance parameters can be derived using uncertainty information extracted by microarray preprocessing software such as the puma package (Pearson et al., 2009) and transformed to variances by minimising the sum of squared deviations of quantiles of absolute expression from puma from corresponding quantiles of a Gaussian as described in Honkela et al. (2010). We call these variances *preprocessing variances*. The gene-specific global variance parameter $\sigma_{m,j}^2$ is called the model-mismatch or adaptive variance as opposed to the fixed data preprocessing variance. This type of variance needs to be estimated and is meant to account for the model mismatch between the specific transcription ODE model and the observed data. When the preprocessing variances are not available from the microarray preprocessing stage, then only the adaptive variance is used. It should be noted that all the expression values in the model are on a non-logarithmic scale.

Let $\widetilde{M}^r \in \mathbb{R}^{J_{tr} \times N_m}$ denote the mRNA measurements of all genes and time points for replicate r, and $M^r \in \mathbb{R}^{J_{tr} \times N_m}$ their noise-free counterparts. The full observation model likelihood is written as

$$p(\{\widetilde{M}^r\}_{r=1}^R | \{M^r\}_{r=1}^R) = \prod_{j=1}^{J_{tr}} \prod_{r=1}^{R} \prod_{n=1}^{N_m} \mathcal{N}(\widetilde{m}_{jn}^r | m_{jn}^r, \sigma_{m,jnr}^2 + \sigma_{m,j}^2), \tag{9}$$

where for the sake of clarity the model parameters $\{\mathbf{k}_j, \mathbf{w}_j, w_{j0}\}_{j=1}^{J_{tr}}$ together with the noise variances that parametrise the likelihood have been omitted from our notation. A special case of this general likelihood is

Symbol(s)	Description	Details
	**ODE functions/parameters**	
$m_j(t)$	mRNA of target gene	Defined in transcription model (1)
$f_i(t), \bar{f}(t)$	TF mRNA (optional)	$f_i(t) = \log(1 + e^{\bar{f}_i(t)})$, where $\bar{f}_i(t) \sim \mathcal{GP}(0, c_i(t, t'))$
$p_i(t), \bar{p}(t)$	TF protein	With translation model defined by (5)<br>If (5) not used, $p_i(t) = \log(1 + e^{\bar{p}_i(t)})$ and $\bar{p}_i(t) \sim \mathcal{GP}(0, c_i(t, t'))$
a_j	Initial conditions	
b_j	Basal transcription rate	
d_j	mRNA degradation rate	
s_j	Transcription sensitivity	
$\mathbf{k}_j, \bar{k}_j$	Kinetic parameters	$\mathbf{k}_j = (a_j, b_j, d_j, s_j)$, $\bar{\mathbf{k}}_j = \log \mathbf{k}_j$
$\delta_i, \bar{\delta}_i$	Translation degradation rate	$\bar{\delta}_i = \log \delta_i$
$\mathbf{w}_j$	Interaction weights	
w_{j0}	Interaction bias	
	**Likelihood noise parameters**	
$\sigma^2_{m,j}$	Adaptive noise variance for target gene mRNA	
$\sigma^2_{m,jnr}$	Preprocessing variance for target gene mRNA	
$\sigma^2_{f,i}$	Adaptive noise variance for TF mRNA	
$\sigma^2_{f,inr}$	Preprocessing variance for TF mRNA	
	**Observed data**	
$\widetilde{m}^r_{jn}$	Observed mRNA at time t_n and replica r	$\widetilde{m}^r_{jn} = m^r_{jn} + \epsilon$, $\epsilon \sim \mathcal{N}(0, \sigma^2_{m,jnr} + \sigma^2_{m,j})$<br>$m^r_{jn} = m^r_j(t_n)$
$\widetilde{f}^r_{in}$	Observed TF mRNA at time t_n and replica r	$\widetilde{f}^r_{in} = f^r_{in} + \epsilon$, $\epsilon \sim \mathcal{N}(0, \sigma^2_{f,inr})$<br>$f^r_{in} = f^r_i(t_n)$
	**Gaussian process (GP) covariance**	
$c_i(t, t')$	GP covariance	$c_i(t, t') = v_i \exp(-(t - t')^2/(2\ell_i^2))$
ℓ_i^2	GP length scale	
v_i	GP variance	
	**Other parameters and notation**	
J_{tr}	Number of genes in the training phase	
R	Number of replicas	
I	Number of TFs	
$\mathcal{T}_m, N_m$	Observation time points	$\mathcal{T}_m = \{t_1, \ldots, t_{N_m}\}$, $N_m = \lvert\mathcal{T}_m\rvert$
$\mathcal{T}_p, N_p$	Points of time discretisation	$\mathcal{T}_p = \{\tau_1, \ldots, \tau_{N_p}\}$, $N_p = \lvert\mathcal{T}_p\rvert$
$\mathbf{x}_j$	Binary vector indicating regulating TFs	$\mathbf{x}_j \in \{0, 1\}^I$
$\mathbf{p}_i, \bar{\mathbf{p}}_i, \mathbf{f}_i, \bar{\mathbf{f}}_i$	Vectors that discretize $p_i(t)$,$\bar{p}_i(t)$,$f_i(t)$,$\bar{f}_i(t)$	

Table 1: Summary of the model parameters and main mathematical notation. The first column shows the mathematical symbols, second column provides a description and the third column gives more technical information.

obtained when the preprocessing variance $\sigma^2_{m,jnr}$ is zero, i.e. not provided.

The above likelihood function uses the transcription ODE and observed data associated with mRNA functions of the training genes. However, typically we also have data for the TF mRNAs which can be used in conjunction with data associated with the translation ODE model. In such case we have an additional likelihood function term. In particular, assuming for simplicity that the observed TF mRNAs are obtained at the same set $\mathcal{T}_m$ of time points as the mRNAs of the target genes, we have

$$\widetilde{f}^r_{in} = \begin{cases} f^r_{in} + \epsilon, \ \epsilon \sim N(0, \sigma^2_{f,inr}) & \text{Preprocessing variances are provided} \\ f^r_{in} + \epsilon, \ \epsilon \sim N(0, \sigma^2_{f,i}) & \text{Otherwise} \end{cases} \tag{10}$$

where $f^r_{in} = f^r_i(t_n)$, with $t_n \in \mathcal{T}_m$. In contrast to mRNA data of the target genes, the TF mRNA are generated by a simpler noise model, which assumes Gaussian noise with variance $\sigma^2_{f,inr}$ or $\sigma^2_{f,i}$ but not $\sigma^2_{f,inr} + \sigma^2_{f,i}$. This is because there is not a potential model-mismatch between $\widetilde{f}^r_{in}$ and f^r_{in}. In other words f^r_{in} is just a value of an unknown time-continuous function $f^r_i(t)$, for which we make no strong model assumptions, e.g. as it would be if this function was given by a specific ODE that would considerably restrict its shape. However, we know that $f^r_i(t)$ takes non-negative values and we expect it to be smooth (e.g. differentiable). As described later, these somehow *weak* assumptions can be modelled by placing a flexible non-parametric prior distribution on all possible shapes for $f^r_i(t)$ through the use of Gaussian processes.

If the matrix $\widetilde{F}^r \in \mathbb{R}^{I \times N_m}$ stores all these observations for replica r and $F^r \in \mathbb{R}^{I \times N_m}$ are their corresponding noise-free values, the above defines a likelihood term given by

$$p(\{\widetilde{F}\}^R_{r=1} | \{F\}^R_{r=1}) = \begin{cases} \prod_{r=1}^R \prod_{i=1}^I \prod_{n=1}^{N_m} \mathcal{N}(\widetilde{f}^r_{in} | f^r_{in}, \sigma^2_{f,inr}) & \text{Preprocessing variances are provided} \\ \prod_{r=1}^R \prod_{i=1}^I \prod_{n=1}^{N_m} \mathcal{N}(\widetilde{f}^r_{in} | f^r_{in}, \sigma^2_{f,i}) & \text{Otherwise.} \end{cases} \tag{11}$$

Notice that the adaptive variance $\sigma^2_{f,i}$ (which needs to be estimated) is only used when the preprocessing variances are not provided.

For the purpose of fitting the model, all data are normalised in the following way. The normalization is done separately for each gene. Specifically, if $\mathbf{m}_j = \{m^r_{jn}\}$ is a vector that stores all observations (across replicas and time points) associated with a gene j, the initial mRNA values are scaled by $\hat{m}_j = \frac{1}{\dim(\mathbf{m}_j)} ||\mathbf{m}_j||^2$ where $|| \cdot ||$ denotes the ℓ_2 (Euclidean) norm and $\dim(\mathbf{m}_j)$ the dimensionality (i.e. number of elements) of the vector $\mathbf{m}_j$. In other words, each initial value m^r_{jn} and the fixed puma variance $\sigma^2_{m,jnr}$ is transformed according to $m^r_{jn} = \frac{m^r_{jn}}{\sqrt{\hat{m}_j}}$ and $\sigma^2_{m,jnr} = \frac{\sigma^2_{m,jnr}}{\hat{m}_j}$ respectively. The normalization of TF mRNAs is done in an exactly analogous way.

3.3 Evaluation of likelihood functions

To evaluate the means of the Gaussian likelihood terms in Eq. (9), we need to compute m^r_{jn} from Eq. (7) which involves an analytically intractable one-dimensional integral. This integral, and therefore $m^r_j(t)$, can be very accurately approximated by applying numerical integration. More precisely, we discretise the time range $[0, t_{N_m}]$ using a very dense grid $\mathcal{T}_p = \{\tau_1, \ldots, \tau_p\}$ where the number of discrete points is typically much larger than the set of time points $\mathcal{T}_m$ associated with observations, i.e. $N_p = |\mathcal{T}_p| \gg N_m$. To simplify the notation, we shall drop the superfix ^{r} from all ms and fs. Moreover, it is convenient to assume that $\mathcal{T}_m \subset \mathcal{T}_p$. By applying numerical integration, m_{jn} is given by

$$m_{jn} = \frac{b_j}{d_j} + \left(a_j - \frac{b_j}{d_j} \right) e^{-d_j t_n} + s_j \sum_{k=1}^{N_{pn}} \frac{h_k}{1 + e^{-w_{j0} - \sum_{i: x_{ji}=1} w_{ji} \log p_{ik}}} e^{-d_j (t_n - \tau_k)}, \tag{12}$$

where $u_1 = 0$, $\tau_{N_{pn}} = t_n$ and $p_{ik} = p_i(\tau_k)$ with $k = 1, \ldots, N_{pn}$. h_k are the weights used in the numerical integration which in our software implementation are defined based on the trapezoid rule. Notice that each TF activity $p_i(t)$ is discretised so that $\mathbf{p}_i = [p_{i1} \ldots p_{iN_p}]^{\mathrm{T}}$ is the N_p-dimensional vector obtained by evaluating $p_i(t)$ at the time points in the set $\mathcal{T}_p$. After the above discretisation, inference over each TF activity $p_i(t)$ has been simplified to inferring the vector $\mathbf{p}_i$. It is worth mentioning that if the ODEs were non-linear we would have to replace the above numerical integration steps with numerical ODE solvers such as Runge-Kutta methods.

When we use the translation ODE model, each TF protein $p_i(t)$ is parametrised by Eq. (6). This implies that the form of $p_i(t)$ deterministically depends on the TF mRNA function $f_i(t)$ and degradation rate δ_i. Thus, to estimate the TF protein $p_i(t)$, we need to estimate the function $f_i(t)$ (for which we have noisy measurements) and the parameter δ_i. Since the integral in Eq. (6) is generally intractable we accurately approximate it by applying numerical integration similarly to the transcription equation so that

$$p_{in} = \sum_{k=1}^{N_{pn}} h_k f_{ik} e^{-\delta_i (t_n - \tau_k)}, \tag{13}$$

where h_k is defined by the trapezoid rule. Thus, the function $f_i(t)$, with $t \in [0, t_{N_m}]$ is represented by a N_p-dimensional vector $\mathbf{f}_i$, while the vector $\mathbf{p}_i$ is obtained deterministically from $\mathbf{f}_i$ according to the above discretised expression.

It will be helpful for the presentation of the next two sections, to clearly state here all the unknown quantities we wish to estimate. We distinguish two cases. The first case is when we do not have observations for the TF mRNA functions and thus only the transcription equation is used. In such case the unknown quantities are the TF protein vectors $\{\mathbf{p}_i\}_{i=1}^{I}$, the ODE parameters $\{\mathbf{k}_j, \mathbf{w}_j, w_{j0}\}_{j=1}^{J_{tr}}$ and the adaptive noise variances $\{\sigma_{m,j}^2\}_{j=1}^{J_{tr}}$. In the second case when the translation ODE model is used the TF protein vectors are determined by the TF mRNA vectors $\{\mathbf{f}_i\}_{i=1}^{I}$ which are now the unknown parameters to be estimated. The remaining unknown ODE parameters are as before with the addition of the protein degradation rates $\{\delta_i\}_{i=1}^{I}$ and the adaptive noise variances $\{\sigma_{f,i}^2\}_{i=1}^{I}$ (if the preprocessing variances are not provided).

3.4 Prior distributions

To infer the ODE models in the training modelling phase, we follow the Bayesian paradigm. This is based on the application of Bayes' rule which combines the likelihood function with a prior distribution over parameters and results in a posterior distribution over the parameters. The posterior distribution is intractable and in Section 3.5 we present a Markov chain Monte Carlo (MCMC) algorithm for simulating from this distribution. Here, we describe in detail the prior distributions over all unknown quantities in the models.

Our prior distribution of the latent continuous-time functions that drive the system is constructed by transforming Gaussian processes. Specifically, the latent function can be either the TF mRNA $f_i(t)$ or the TF protein $p_i(t)$. If the TF protein translation model is used, then $f_i(t)$ is the latent function, while $p_i(t)$ is obtained deterministically from $f_i(t)$ and the solution to the translation ODE (Eq. (6)). In such a case we need to place a prior distribution on the function $f_i(t)$. If the TF protein translation model is not used, then $p_i(t)$ is directly the latent function, and we need to place a prior distribution on $p_i(t)$.

As $f_i(t)$ is known to be positive, we define its prior using a transformation that imposes non-negativity constraints on a GP. Specifically,

$$f_i(t) = \phi(\bar{f}_i(t)), \tag{14}$$

where $\phi : \mathbb{R} \to \mathbb{R}^+$ is $\phi(u) = \log(1 + e^u)$. The GP prior is then placed on $\bar{f}_i(t)$ which through the above

transformation induces an non-GP prior on $f_i(t)$ which ensures non-negativity. The GP prior assigned to $\bar{f}_i(t)$ has a zero mean function and the squared-exponential covariance function given by

$$c_i(t, t') = v_i \exp\left\{ -\frac{1}{2\ell_i^2}(t - t')^2 \right\}, \tag{15}$$

where v_i is the variance and ℓ_i^2 the length scale of the process.

If the TF protein translation model is not used and there are no observations for TF protein activity, then the GP prior is placed on $\bar{p}_i(t)$ which through the transformation $\phi(\cdot)$ induces a non-GP prior on $p_i(t)$. In such case the model can be further simplified by fixing $v_i = 1$. This fixes a scaling indeterminacy with the bias parameter w_{j0} by fixing the scale of the protein activities.

Since, the likelihood models in Eqs. (12) and (11) require reference to the finite vector of values $\bar{\mathbf{f}}_i$ (or $\bar{\mathbf{p}}_i$), the GP prior reduces to the following multivariate Gaussian distribution:

$$p(\bar{\mathbf{f}}_i|\ell_i^2, v_i) = \mathcal{N}(\bar{\mathbf{f}}_i|\mathbf{0}, C_i), \tag{16}$$

where C_i is the $N_p \times N_p$ covariance obtained by evaluating the covariance function in Eq. (15) at the set of time points $\mathcal{T}_p$. The prior $p(\bar{\mathbf{f}}_i|\ell_i^2, v_i)$ (or $p(\bar{\mathbf{p}}_i|\ell_i^2, v_i = 1)$) together with the transformation $\phi(\cdot)$ induce a prior over the vector $\mathbf{f}_i$ (or $\mathbf{p}_i$) which is the actual quantity that appears in the likelihood functions.

3.4.1 Prior for the remaining parameters

Here, we discuss the prior distributions assigned to the parameters of the ODE models, i.e. the kinetic parameters $\mathbf{k}_j = (a_j, b_j, d_j, s_j)$, the interaction weights $\mathbf{w}_j$ and the bias parameter w_{j0}. When the translation ODE model is used we also need to place a prior distribution on each degradation rate δ_i.

The kinetic parameters $\mathbf{k}_j$ are non-negative and we represent them in the log space so as $\bar{\mathbf{k}}_j = \log \mathbf{k}_j$ is the actual parameter. Then, Gaussian priors are assigned to $\bar{\mathbf{k}}_j$:

$$p(\bar{\mathbf{k}}_j) = \prod_{\bar{k}_{ji} \in \bar{\mathbf{k}}_j} \mathcal{N}(\bar{k}_{ji}|a, b^2), \tag{17}$$

where the mean a and the variance b^2 of the Gaussian prior are set to give a vague prior and attain common values for all genes. In all our experiments we used $a = -0.5$ and $b^2 = 2$. Assuming time is measured in hours, the prior for d_j translates into mRNA half-life distribution with 5% and 95% quantiles of 4 minutes and 19 hours, respectively.

Each bias parameter w_{j0}, which takes real values, is assigned a zero-mean Gaussian prior so as $p(w_{j0}) = \mathcal{N}(w_{j0}|0, s^2)$ and where $s^2 = 2$ was used in all the experiments. For the prior of interaction weights $\mathbf{w}_j$ we have several options that can be useful in practice. The simplest choice is to assign a zero-mean Gaussian prior independently for each interaction weight w_{ji} so that $p(\mathbf{w}_j) = \prod_{i=1}^{I} \mathcal{N}(w_{ji}|0, s^2)$. This option allows the interaction weights to attain both negative and positive values which implies that the TFs can act either as repressors or activators. This choice for the prior was used in the artificial data set; see main paper and Section 5.1 in this report. In many cases, it can be useful to constrain the interaction weights so that to express prior knowledge that certain TFs can act only as activators or repressors. For instance, if it is known that a TF i never represses the gene j, then $w_{ji} \geq 0$. Such a prior can be defined by the following truncated Gaussian

$$p(w_{ji}) = 2\mathcal{N}(w_{ji}|0, s^2) I(w_{ji} \geq 0),$$

where $I(w_{ji} \geq 0)$ is the indicator function. A prior that constrains a TF to be only a repressor can be defined similarly. The training modelling phase in *Drosophila* experiments allows the TF to act both as repressors and activators. However, in the prediction phase only activation is allowed and the above truncated priors are used. The variance hyperparameter s^2 in the prior over the interaction weights was set to the value two in all our experiments.

Each adaptive variance parameter $\sigma^2_{m,j}$, in the likelihood in (9), is assigned an independent conjugate inverse gamma prior each having the form

$$p(\sigma^2_{m,j}) = \frac{\beta^\alpha}{\Gamma(\alpha)} \frac{e^{-\beta/\sigma^2_{m,j}}}{(\sigma^2_{m,j})^{\alpha+1}}, \tag{18}$$

where the hyperparameters α and β where both set to the value 0.01 that gives a vague prior.

When the TF mRNA observations $\widetilde{F}_i$ are provided and the translation ODE is used we need to define prior distributions for the degradation rates $\{\delta_i\}_{i=1}^I$ and the noise variances $\{\sigma^2_{f,i}\}_{i=1}^I$ (if the preprocessing variances are not provided). Each degradation rate obtains the same prior with the remaining kinetic parameters $\mathbf{k}_j$, while each $\sigma^2_{f,i}$ is assigned the same vague inverse gamma prior as each $\sigma^2_{m,j}$. Finally each length-scale parameter ℓ_i^2 is assigned a uniform prior in the range $[a_{\ell^2}, b_{\ell^2}]$ where a_{ℓ^2} and b_{ℓ^2} are chosen as follows. To define a_{ℓ^2} we first compute the minimum time interval between consecutive observed measurements, expressed $\min\{t_{i+1} - t_i; i =, \ldots, N_m - 1\}$, and then set a_{ℓ^2} to be the square of that value. b_{ℓ^2} is defined to be the square of whole observation time interval, i.e. $b_{\ell^2} = (t_{N_m} - t_1)^2$. This uniform distribution restricts the value of length scale parameter to be inside a sensible range that takes into account the sampling rate of the mRNA observations, which is assumed to match the time scale of phenomena of interest.

3.5 Markov Chain Monte Carlo

In this section we discuss the Markov Chain Monte Carlo (MCMC) algorithm that is used to estimate the unknown parameters. Before describing the steps of the MCMC algorithm, let us summarise the model by clearly writing down the joint probability density of all variables and give a brief explanation of its components. For simplicity, we assume a single replica for the mRNA time-series, while dealing with multiple replicas is straightforward and will be discussed at the end of the section. For the most general case when the observed data consists of both mRNAs of target genes and TF mRNAs, the joint probability model takes the form

$$p(\widetilde{M}|\bar{F}, \bar{K}, W, \mathbf{w}_0, \bar{\boldsymbol{\delta}}, \Sigma_m) p(\widetilde{F}|\bar{F}, \Sigma_f) p(\bar{F}|V, L) p(\bar{K}) p(W) p(\mathbf{w}_0) p(\bar{\boldsymbol{\delta}}) p(\Sigma_m, \Sigma_f, V) p(L). \tag{19}$$

Here, $p(\widetilde{M}|\bar{F}, \bar{K}, W, \mathbf{w}_0, \bar{\boldsymbol{\delta}}, \Sigma_m)$ is the likelihood from Eq. (9) (recall that here we assume a single replica) where we explicitly have written the dependence on the random variables that deterministically define M via equations (13) and (12). These random variables are the vectors $\bar{F} = \{\bar{\mathbf{f}}_i\}_{i=1}^I$ (which define the TF mRNAs through the transformation $\log(1 + e^u)$), the logarithms of the transcription ODE kinetic parameters $\bar{K} = \{\bar{\mathbf{k}}_j\}_{j=1}^{J_{tr}}$, the interaction weights $W = \{\mathbf{w}_j\}_{j=1}^{J_{tr}}$, the biases $\mathbf{w}_0 = \{w_{j0}\}_{j=1}^{J_{tr}}$, the logarithms of the protein degradation rates $\bar{\boldsymbol{\delta}} = \{\bar{\delta}_i\}_{i=1}^I$ and noise variances $\Sigma_m = \{\sigma^2_{m,j}\}_{j=1}^{J_{tr}}$. $p(\widetilde{F}|\bar{F}, \Sigma_f)$ is the likelihood function defined in Eq. (11) where $\Sigma_f = \{\sigma^2_{f,i}\}_{i=1}^I$. $p(\bar{F}|\mathbf{v}, L) = \prod_{i=1}^I p(\bar{\mathbf{f}}_i|v_i, \ell_i^2)$ is the product of conditionally independent GP priors each given by Eq. (16). The prior distributions $p(\bar{K})$, $p(W)$, $p(\mathbf{w}_0)$ and $p(\bar{\boldsymbol{\delta}})$ are defined similarly so that they are fully independent (across individual random variables) with each factor defined in Section 3.4. $p(\Sigma_m, \Sigma_f, V)$ is fully independent prior distribution over all noise variances in the two likelihoods (Σ_m and Σ_f) and the kernel variances $V = \{v_i\}_{i=1}^I$. Each factor in this prior follows the inverse gamma given by Eq. (18). $p(L)$, with $L = \{\ell_i^2\}_{i=1}^I$, denotes the length scales of the GP covariance functions which follow the uniform distribution

defined in Section 3.4.1.

When the TF mRNA observations and the translation model is not used, the joint probability density in Eq. (19) simplifies as follows

$$p(\widetilde{M}|\bar{P}, \bar{K}, W, \mathbf{w}_0, \Sigma_m)p(\bar{P}|L)p(\bar{K})p(W)p(\mathbf{w}_0)p(\Sigma_m)p(L). \tag{20}$$

Here, $p(\widetilde{M}|\bar{P}, \bar{K}, W, \mathbf{w}_0, \Sigma_m)$ denotes the simplified likelihood for the mRNA measurements of the target genes that directly depend on the protein vector $\bar{P} = \{\bar{\mathbf{p}}_i\}_{i=1}^{I}$. These vectors are now the latent variables assigned a GP prior $p(\bar{P}|L) = \prod_{i=1}^{I} p(\bar{\mathbf{p}}_i|\ell_i^2)$ where $p(\bar{\mathbf{p}}_i|\ell_i^2)$ is given by Eq. (16). Notice also that the kernel variance parameter V are fixed to unity in this case as indicated by the covariance function in Eq. (15).

Next we discuss the MCMC algorithm which simulates from the posterior distribution of all unknown variables. We will present the algorithm for the most general case where we wish to sample from the posterior distribution associated with the density in Eq. (19). The other cases are simpler and involve fewer steps. Our MCMC algorithm is based on iteratively updating different subsets of the unknown random variables using the Metropolis-Hastings (MH) algorithm. Most of the updates involve a proposal distribution and acceptance or rejection based on the MH probability. Few simple updates, that arise in spacial cases, use as the proposal distribution an exact conditional posterior and thus they correspond to Gibbs sampling updates.

Next we present the steps involved in a single iteration of the algorithm that cycles through all parameters. For each step we give the part of the joint density from Eq. (19) that is needed in the MH probability (the remaining part cancels out) and the form of the proposal distribution.

1. **Untransformed TF mRNA vectors** $\bar{F}$: For $i = 1, \ldots, I$ sample $\bar{\mathbf{f}}_i$. The relevant part of the joint density is

$$p(\widetilde{M}|\bar{\mathbf{f}}_i, \{\bar{\mathbf{f}}_j\}_{j\neq i}, \bar{K}, W, \mathbf{w}_0, \bar{\boldsymbol{\delta}}, \Sigma_m)p(\widetilde{\mathbf{f}}_i|\bar{\mathbf{f}}_i, \sigma_{f,i}^2)p(\bar{\mathbf{f}}_i|v_i, \ell_i^2). \tag{21}$$

   The distribution that proposes a new $\bar{\mathbf{f}}_i'$ given the current value $\bar{\mathbf{f}}_i$ is chosen to have the form

$$Q(\bar{\mathbf{f}}_i'|\bar{\mathbf{f}}_i) \propto H(\bar{\mathbf{f}}', \bar{\mathbf{f}})p(\bar{\mathbf{f}}_i'|v_i, \ell_i^2), \tag{22}$$

   where $p(\bar{\mathbf{f}}_i'|v_i, \ell_i^2)$ is the GP prior that appears also in the part of the joint density and $H(\bar{\mathbf{f}}', \bar{\mathbf{f}})$ is a symmetric and log-quadratic function with respect to its arguments. Symmetry here means that $H(\bar{\mathbf{f}}', \bar{\mathbf{f}}) = H(\bar{\mathbf{f}}, \bar{\mathbf{f}}')$. The way that $H(\bar{\mathbf{f}}', \bar{\mathbf{f}})$ is constructed is discussed in the Appendix A. The above proposal distribution has the elegant property that it is invariant under the GP prior $p(\bar{\mathbf{f}}_i|v_i, \ell_i^2)$. This means that the proposed values for $\bar{\mathbf{f}}_i$ are probable samples from $p(\bar{\mathbf{f}}_i|v_i, \ell_i^2)$ and the GP prior terms cancel out from the MH probability. More precisely, a proposed $\bar{\mathbf{f}}'$ is accepted with probability

$$\begin{aligned} &\min\left\{1, \frac{p(\widetilde{M}|\bar{\mathbf{f}}_i', \{\bar{\mathbf{f}}_j\}_{j\neq i}, \bar{K}, W, \mathbf{w}_0, \bar{\boldsymbol{\delta}}, \Sigma_m)p(\widetilde{\mathbf{f}}_i|\bar{\mathbf{f}}_i', \sigma_{f,i}^2)p(\bar{\mathbf{f}}_i'|v_i, \ell_i^2)H(\bar{\mathbf{f}}, \bar{\mathbf{f}}')p(\bar{\mathbf{f}}_i|v_i, \ell_i^2)}{p(\widetilde{M}|\bar{\mathbf{f}}_i, \{\bar{\mathbf{f}}_j\}_{j\neq i}, \bar{K}, W, \mathbf{w}_0, \bar{\boldsymbol{\delta}}, \Sigma_m)p(\widetilde{\mathbf{f}}_i|\bar{\mathbf{f}}_i, \sigma_{f,i}^2)p(\bar{\mathbf{f}}_i|v_i, \ell_i^2)H(\bar{\mathbf{f}}', \bar{\mathbf{f}})p(\bar{\mathbf{f}}_i'|v_i, \ell_i^2)}\right\} \\ = \; &\min\left\{1, \frac{p(\widetilde{M}|\bar{\mathbf{f}}_i', \{\bar{\mathbf{f}}_j\}_{j\neq i}, \bar{K}, W, \mathbf{w}_0, \bar{\boldsymbol{\delta}}, \Sigma_m)p(\widetilde{\mathbf{f}}_i|\bar{\mathbf{f}}_i', \sigma_{f,i}^2)}{p(\widetilde{M}|\bar{\mathbf{f}}_i, \{\bar{\mathbf{f}}_j\}_{j\neq i}, \bar{K}, W, \mathbf{w}_0, \bar{\boldsymbol{\delta}}, \Sigma_m)p(\widetilde{\mathbf{f}}_i|\bar{\mathbf{f}}_i, \sigma_{f,i}^2)}\right\}, \end{aligned} \tag{23}$$

   where the quantities involved are the full likelihood of the mRNA measurements of the target genes and the likelihood term from Eq. (11) that contains the TF mRNAs for the ith TF.

2. **Logarithm of translation ODE degradation rates** $\bar{\boldsymbol{\delta}}$**:** For $i = 1, \ldots, N$ sample $\bar{\delta}_i$, which is the

logarithm of actual degradation rate δ_i. The relevant part of the joint density is

$$p(\widetilde{M}|\bar{\mathbf{f}}_i, \{\bar{\mathbf{f}}_j\}_{j\neq i}, \bar{K}, W, \mathbf{w}_0, \bar{\boldsymbol{\delta}}, \Sigma_m)p(\bar{\delta}_i). \tag{24}$$

The proposal distribution $Q(\bar{\delta}_i|\bar{\delta}_i)$ is a Gaussian:

$$Q(\bar{\delta}_i'|\bar{\delta}_i) = \mathcal{N}(\bar{\delta}_i'|\bar{\delta}_i, h_{\delta_i}), \tag{25}$$

where the scale h_{δ_i} is adapted using preliminary runs in order to achieve an acceptance rate around 0.25. Notice that since the proposal distribution is a symmetric Gaussian, the terms of the proposal distribution cancel out from the MH probability.

3. **Logarithm of transcription ODE kinetic parameters $\bar{K}$:** For $j = 1, \ldots, J_{tr}$ sample the vector $\bar{\mathbf{k}}_j$ which is the logarithm of the actual kinetic parameters $\mathbf{k}_j$. The relevant part of the joint density is

$$p(\widetilde{\mathbf{m}}_j|\bar{F}, \bar{\mathbf{k}}_j, \mathbf{w}_j, w_{j0}, \bar{\boldsymbol{\delta}}, \sigma^2_{m,j})p(\bar{\mathbf{k}}_j), \tag{26}$$

where $p(\widetilde{\mathbf{m}}_j|\bar{F}, \bar{\mathbf{k}}_j, \mathbf{w}_j, w_{j0}, \bar{\boldsymbol{\delta}}, \sigma^2_{m,j})$ is the likelihood term that depends only on the mRNA data of gene j. The proposal distribution is chosen to be an isotropic Gaussian of the form

$$Q(\bar{\mathbf{k}}_k'|\bar{\mathbf{k}}_j) = \mathcal{N}(\bar{\mathbf{k}}_j'|\bar{\mathbf{k}}_j, h_{k_j}I), \tag{27}$$

where the scale h_{k_j} is adapted using preliminary runs to achieve an acceptance rate around 0.25. Notice that since the proposal distribution is a symmetric Gaussian, the proposal distribution terms cancel out from the MH probability.

4. **Interaction weights W and biases $\mathbf{w}_0$:** For $j = 1, \ldots, J_{tr}$ sample the I-dimensional vector $\mathbf{w}_j$ and the bias parameter w_{j0}. The relevant part of the joint density is

$$p(\widetilde{\mathbf{m}}_j|\bar{F}, \bar{\mathbf{k}}_j, \mathbf{w}_j, w_{j0}, \bar{\boldsymbol{\delta}}, \sigma^2_{m,j})p(\mathbf{w}_j)p(w_{j0}). \tag{28}$$

The proposal distribution takes the form $Q(\mathbf{w}_j', w_{j0}'|\mathbf{w}_j, w_{j0}) = Q(\mathbf{w}_j'|\mathbf{w}_j)Q(w_{j0}'|w_{j0})$. Since the bias w_{j0} is always a real-valued parameter, $Q(w_{j0}'|w_{j0})$ is chosen to be a univariate Gaussian:

$$Q(w_{j0}'|w_{j0}) = \mathcal{N}(w_{j0}'|w_{j0}, h_{w_j}). \tag{29}$$

The proposal distribution $Q(\mathbf{w}_j'|\mathbf{w}_j)$ for the interaction weights is either a Gaussian when $\mathbf{w}_j$ is unrestricted or a truncated Gaussian when $\mathbf{w}_j$ takes only non-negative values. Thus,

$$Q(\mathbf{w}_j'|\mathbf{w}_j) = \begin{cases} \mathcal{N}(\mathbf{w}_j'|\mathbf{w}_j, h_{w_j}I) & \mathbf{w}_j \in \mathbb{R}^I \\ \frac{\mathcal{N}(\mathbf{w}_j'|\mathbf{w}_j, h_{w_j}I)I(\mathbf{w}_j \geq 0)}{\int \mathcal{N}(\mathbf{z}|\mathbf{w}_j, h_{w_j}I)I(\mathbf{z}\geq 0)\,d\mathbf{z}} & \mathbf{w}_{ji} \geq \mathbf{0}. \end{cases} \tag{30}$$

The scale parameter h_{w_j} is adapted using preliminary runs to achieve an acceptance rate around 0.25. The proposal distribution for the bias w_{j0} is always a symmetric Gaussian and therefore it cancels out from the MH probability. For the interaction weights, the proposal distribution is symmetric only $\mathbf{w}_j$ is unrestricted. When $\mathbf{w}_j$ is restricted to non-negative values, we need to include the terms from the proposal distribution in the MH probability since the truncated Gaussian is not symmetric. Also, sampling from

the above truncated Gaussian can be easily and efficiently done by using rejection sampling as discussed in the Appendix B.

5. **Adaptive variances for the TF mRNAs Σ_f:** (this step is performed only if the preprocessing variances $\{\sigma^2_{f,inr}\}$ are not available). Each parameter $\sigma^2_{f,i} \in \Sigma_f$ is sampled using Gibbs sampling. Specifically, $\sigma^2_{f,i}$ has an inverse gamma conditional posterior density with hyperparameters

$$\alpha_{fi} = \alpha + \frac{1}{2} N_m$$

$$\beta_{fi} = \beta + \frac{1}{2} \sum_{n=1}^{N_m} (\widetilde{f}_{in} - f_{in})^2,$$

where the noise-free TF mRNA f_{in} depends on $\bar{f}_{in}$ through the transformation $f_{in} = \log(1 + e^{\bar{f}_{in}})$.

6. **Noise variances Σ_m.** Here, we distinguish two cases:

   (a) **The preprocessing variances are not provided, i.e. each $\sigma^2_{m,jnr}$ is zero:** Σ_m is sampled using Gibbs sampling updates since the conditional posterior is an inverse gamma distribution. Specifically, each parameter $\sigma^2_{m,j} \in \Sigma_m$ has conditional posterior density of the form of Eq. (18) with updated hyperparameters:

$$\alpha_{mj} = \alpha + \frac{1}{2} N_m$$

$$\beta_{mj} = \beta + \frac{1}{2} \sum_{n=1}^{N_m} (\widetilde{m}_{jn} - m_{jn})^2,$$

   where the noise-free mRNA m_{jn} is obtained from the ODE and is a function of the current values of $(\bar{F}, \bar{k}_j, \mathbf{w}_j, w_{j0}, \bar{\boldsymbol{\delta}})$.

   (b) **Preprocessing variances are provided:** Here, the conditional posterior distribution for Σ_m does not have a conjugate form and Gibbs sampling is intractable. Thus, we use MH updates. For each parameter $\sigma^2_{m,j}$ the relevant part of the joint density is

$$p(\widetilde{\mathbf{m}}_j | \bar{F}, \bar{\mathbf{k}}_j, \mathbf{w}_j, w_{j0}, \bar{\boldsymbol{\delta}}, \sigma^2_{m,j}) p(\sigma^2_{m,j}). \tag{31}$$

   The proposal distribution $Q(\sigma^{2'}_{m,j} | \sigma^2_{m,j})$ is a truncated Gaussian distribution of the form

$$Q(\sigma^{2'}_{m,j} | \sigma^2_{m,j}) \propto \mathcal{N}(\sigma^{2'}_{m,j} | \sigma^{2'}_{m,j}, h_{\sigma_{m,j}}) I(\sigma^{2'}_{m,j} > 0),$$

   where the scale $h_{\sigma_{m,j}}$ is adapted using preliminary runs in order to achieve an acceptance rate around 0.25.

7. **Length scales L and variances V in the GP covariance functions:** For $i = 1, \ldots, I$ sample jointly the parameter $\ell^2_i \in L$ and $v_i \in V$. The relevant part of the joint density is

$$p(\bar{\mathbf{f}}_i | v_i, \ell^2_i) p(v_i) p(\ell^2_i), \tag{32}$$

where $p(\bar{\mathbf{f}}_i | v_i, \ell^2_i)$ is the GP prior and $p(v_i)$ and $p(\ell^2_i)$ are inverse gamma prior distributions. The proposal distribution takes the form $Q(v'_i, \ell^{2'}_i | v_i, \ell^2_i) = Q(v'_i | v_i) Q(\ell^{2'}_i | \ell^2_i)$ where $Q(v'_i | v_i)$ and $Q(\ell^{2'}_i | \ell^2_i)$ are both

truncated Gaussian distributions of the form

$$Q(v_i'|v_i) \propto \mathcal{N}(v_i'|v_i, h_{c_i})I(v_i > 0)$$

$$Q(\ell_i^{2'}|\ell_i^2) \propto \mathcal{N}(\ell_i^{2'}|\ell_i^2, h_{c_i})I(\ell_i^{2'} > 0).$$

The common scale h_{c_i} is adapted using preliminary runs in order to achieve an acceptance rate around 0.25. Notice the above truncated Gaussians are not symmetric and thus they must be included in the MH probability.

Some additional technical details about the steps of the above algorithm are given in the Appendix A and B as was indicated above.

To conclude this section, we need to discuss how the above algorithm is modified under situations that arise with the absence of TF mRNA measurements and multiple replicas. When the TF mRNA measurements are not available then steps 2 and 5 are not needed while steps 1 and 2 need slight modifications. More precisely, the variables $\bar{F}$ do not exist and the GP prior are placed on $\bar{P}$ that directly model the discretised TF protein vector P via the transformation $\log(1 + e^u)$. Also the GP prior depend only on the length scale parameter ℓ_i^2 while each variance parameter v_i is fixed to unity. Step 1 is modified so that the relevant part of the joint density is

$$p(\widetilde{M}|\bar{\mathbf{p}}_i, \{\bar{\mathbf{p}}_j\}_{j\neq i}, K, W, \mathbf{w}_0, \Sigma_m)p(\bar{\mathbf{p}}_i|\ell_i^2). \tag{33}$$

The proposal distribution remains of an analogous form, i.e. $Q(\bar{\mathbf{p}}_i'|\bar{\mathbf{p}}_i) \propto H(\bar{\mathbf{p}}', \bar{\mathbf{p}})p(\bar{\mathbf{p}}_i'|\ell_i^2)$ and the MH probability depends only on the likelihood ratio. Finally, step 7 is modified so that we sample only the length scales L of the GP covariance function since the variances V are fixed to unity.

Finally we consider the case when we have multiple replicas of the mRNA time-series data. Slight modifications of the algorithms are needed in such case. When we have R replicas, then we need to sample $R \times I$ GP function vectors instead of just I vector. Thus, step 1 is modified so that to iterate over all $R \times I$ vectors. Otherwise, this step is exactly as described above with the only difference that $\bar{\mathbf{f}}$ and $\widetilde{M}$ are indexed by the replica r where $r = 1, \ldots, R$. The remaining steps 2-7 deal with sampling parameters that are shared across replicas and the modifications are needed are straightforward. For instance, in the steps 2-6 the presence of the likelihood term $p(\widetilde{M}|\bar{F}, \bar{K}, W, \mathbf{w}_0, \boldsymbol{\delta}, \Sigma_m)$ will be replaced by $\prod_{r=1}^R p(\widetilde{M}^r|\bar{F}^r, \bar{K}, W, \mathbf{w}_0, \bar{\boldsymbol{\delta}}, \Sigma_m)$ and for the step 7, the presence of $p(\bar{\mathbf{f}}_i|v_i, \ell_i^2)$ is replaced by $\prod_{r=1}^R p(\bar{\mathbf{f}}_i^r|v_i, \ell_i^2)$.

4 *Prediction phase:* Genome-wide inference of network connectivity

Here, we discuss how the models, trained according to the first phase, can be used to predict the network connectivity in a set of *test* genes. This involves a genome-wide screening or scanning where typically the number of test genes that need to be processed is of the order of thousands. Next, we discuss all the steps involved in the prediction phase.

Suppose we have trained the model in a small set of genes as described in Section 3. This training phase provides an estimate of the TF profiles, i.e. the vectors $\{\mathbf{p}_i\}_{i=1}^I$. Specifically, the output of the MCMC algorithm in Section 3.5 is a set of T samples for all parameters. Let $\{\mathbf{f}_i^{(1)}, \ldots, \mathbf{f}_i^{(N)}\}$ be the posterior samples for the ith TF mRNA vectors (each obtained from $\bar{\mathbf{f}}_i^{(t)}$ through the transformation $\log(1 + e^u)$) and $\{\delta_i^{(1)}, \ldots, \delta_i^{(N)}\}$ the samples of the corresponding degradation rates. These two sets deterministically define the set of posterior

samples $\{\mathbf{p}_i^{(1)}, \ldots, \mathbf{p}_i^{(N)}\}$ for the TF proteins through Eq. (13). The latter set is the only thing needed from the training phase when we do predictions in the test genes.

The prediction phase is performed independently for each test gene, thus it is sufficient to describe how we deal with a single test gene. Let $*$ denote a test gene so that $\widetilde{\mathbf{m}}_* \in \mathbb{R}^{N_m}$ is the vector of observed mRNA measurements. This gene can be regulated by any combination of the I TFs. Let $\mathbf{x}_* \in \{0,1\}^I$ be a binary vector that indicates the subset of the TFs that regulate gene $*$. The vector $\mathbf{x}_*$ can take 2^I possible values. To infer the network links, it suffices to compute the posterior distribution over the random variable $\mathbf{x}_*$. This distribution can be written in the form

$$p(\mathbf{x}_*|\widetilde{\mathbf{m}}_*, \text{tr-data}) = \frac{p(\mathbf{x}_*, \widetilde{\mathbf{m}}_*|\text{tr-data})}{\sum_{\mathbf{x}} p(\mathbf{x}, \widetilde{\mathbf{m}}_*|\text{tr-data})} = \frac{p(\widetilde{\mathbf{m}}_*|\mathbf{x}_*, \text{tr-data}) p(\mathbf{x}_*|\text{tr-data})}{\sum_{\mathbf{x}} p(\widetilde{\mathbf{m}}_*|\mathbf{x}, \text{tr-data}) p(\mathbf{x}|\text{tr-data})}, \tag{34}$$

where "tr-data" indicates the data used in the training modelling phase i.e. the observed mRNAs $\widetilde{M}$, the observed TF mRNAs $\widetilde{F}$ (if available) and the known network connections for the training genes represented by the vectors $\{\mathbf{x}_j\}_{j=1}^{J_{tr}}$. To obtain the above posterior probabilities we need to compute the predictive density $p(\widetilde{\mathbf{m}}_*|\mathbf{x}_*, \text{tr-data})$ for any possible combination of regulating TFs, represented by $\mathbf{x}_*$, together with the associated probability $p(\mathbf{x}_*|\text{tr-data})$. The probability $p(\mathbf{x}_*|\text{tr-data})$ can be computed by the frequencies of the known connectivity vectors $\{\mathbf{x}_j\}_{j=1}^{J_{tr}}$ in the training genes. However, such a specification of the above probabilities is unreliable since the small set of training genes may not be representative[1] about the prior distribution of links between TF and genes. Therefore, for simplicity we set these probabilities to uniform values so that Eq. (34) simplifies to

$$p(\mathbf{x}_*|\widetilde{\mathbf{m}}_*, \text{tr-data}) = \frac{p(\widetilde{\mathbf{m}}_*|\mathbf{x}_*, \text{tr-data})}{\sum_{\mathbf{s}} p(\widetilde{\mathbf{m}}_*|\mathbf{x}, \text{tr-data})}. \tag{35}$$

This reveals that the posterior probability of a certain network configuration is proportional to its predictive density value $p(\widetilde{\mathbf{m}}_*|\mathbf{x}_*, \text{tr-data})$. The latter quantity is a type of marginal likelihood which is very hard to compute. Therefore, we will need to apply some approximate method for computing marginal likelihoods. In Section 4.1, we present a novel approximation we have developed, which is based on the outcome of MCMC.

Given the probabilities $p(\mathbf{x}_*|\widetilde{\mathbf{m}}_*, \text{tr-data})$, with $\mathbf{x}_* \in \{0,1\}^I$ any query related to the regulating TFs can be answered by this posterior distribution. For instance in the main article we made use of the following quantities:

- Maximum a posteriori (MAP) network configuration: This is the most probable setting $\mathbf{x}_*^{\text{MAP}}$ for the network links obtained by
$$\mathbf{x}_*^{\text{MAP}} = \arg\max_{\mathbf{x}_*} p(\mathbf{x}_*|\widetilde{\mathbf{m}}_*, \text{tr-data}).$$

- Marginal probability of a single link: The link between the test gene and the ith TF is present with posterior probability
$$p(x_{*i} = 1|\widetilde{\mathbf{m}}_*, \text{tr-data}) = \sum_{\mathbf{x}_*: x_{*i}=1} p(\mathbf{x}_*|\widetilde{\mathbf{m}}_*, \text{tr-data}). \tag{36}$$
This probability was used extensively in the main paper to carry out single-TF links prediction. In particular, the ROC curves in Figure 4 were produced by classifying the network links as present or absent based on the above probabilities. Figure 7(a) (main paper) and Figure 16 in this report use these probabilities for gene ranking and enrichment predictions. This was done by first computing the most probable link probability (according to $\max_i p(x_{*i} = 1|\widetilde{\mathbf{m}}_*, \text{tr-data})$) for each test gene and then ranking the genes based on the latter probabilities.

[1]Recall that the assumption we made about the training genes was that they consist of a set of well-studied genes rather than a random set of genes, which would have been more representative about the distribution of network links.

- Marginal probability of a pair of links: Similarly to the single-TF links we can compute marginal probabilities for a joint link involving two TFs. For instance, the links from the ith and k^{th} TF are both present with posterior probability

$$p(x_{*i} = 1, x_{*k} = 1 | \widetilde{\mathbf{m}}_*, \text{tr-data}) = \sum_{\mathbf{x}_* : x_{*i}=1, x_{*k}=1} p(\mathbf{x}_* | \widetilde{\mathbf{m}}_*, \text{tr-data}). \tag{37}$$

  The probabilities were used to produce the Figure 7(b) (main paper) and Figure 17 in this report exactly analogously to the case of single-TF links.

In the results described in the main article we made use of the following predictive methods: "MAP-32", "Posterior-32", "Posterior-2" and "Posterior-4". In the light of the above definitions, we now give a more precise definition of what these names represent. All these methods were used in the *Drosophila* data set where each test gene can be regulated by five TFs, and therefore $\mathbf{x}_*$ takes $2^5 = 32$ possible values. This set of 32 configurations comprise the hypothesis or model space corresponding to all possible networks. The label "32" indicates that the full space of 32 models was used in the computation of the posterior probabilities, i.e. precisely as described above. The label "2" and "4" indicates that smaller model spaces were used for the computation of the posterior probabilities by the respective methods.

More precisely, "MAP-32" method (see Drosophila data results section of the main article) corresponds to globally ranking genes and network configurations according to their posterior probability $p(\mathbf{x}_* | \widetilde{\mathbf{m}}_*, \text{tr-data})$. This means that a certain gene and a configuration setting for its network connectivity is ranked first if the associated posterior probability is larger than any posterior probability across all test gene and network configurations. In the *Drosophila* data where we have 6003 test genes and 32 network configurations, there exist 6003×32 probability values. The maximum probability values for each gene are sorted in descending order giving rise to the ranking in Figure 6 (main paper). Similarly, the "Posterior-32" method computes a posterior probability of an event, such as a single-TF link being present (see Eq. (36)) or a pair of TFs being present (see Eq. (37)), and such a computation involves a space consisting of all 32 models. The "Posterior-2" and "Posterior-4" methods compute posterior probabilities similarly but within a more restricted space of values of $\mathbf{x}_*$. Specifically, the "Posterior-2" method was used for single-TF link prediction where the posterior probability $p(x_{*i} = 1 | \widetilde{\mathbf{m}}_*, \text{tr-data})$ was computed by having a space of only two models: a model that has the ith TF as the only regulating TF (i.e. $x_{*i} = 1$ and $x_{*k} = 0$, $k \neq i$) and a baseline or null model with no regulating TFs (see Section 4.1). The "Posterior-4" method was used for pair-TF link prediction where the posterior probability $p(x_{*i} = 1, x_{*k} = 1 | \widetilde{\mathbf{m}}_*, \text{tr-data})$ was computed by having model space of four models: a model that has the ith and k^{th} TFs as the only regulating TFs, the two single-TF models having only either i^{th} or k^{th} TF and a baseline model with no regulating TFs.

4.1 Computation of the predictive density

To compute Bayesian posterior probabilities according to Eq. (35), we need to be able to compute the predictive density $p(\widetilde{\mathbf{m}}_* | \mathbf{x}_*, \text{tr-data})$. As explained shortly, this quantity is a type of a marginal likelihood that is intractable to compute. Approximate methods are necessary and for our purposes they must not only be reasonably accurate but also sufficiently fast. The latter is essential to make genome-wide scanning possible. As an example of the number of marginal likelihoods we need to compute, recall that in the *Drosophila* data set we need to compute $6003 \times 32 = 192096$ predictive densities.

To start with, suppose we have a test gene and we wish to estimate $p(\widetilde{\mathbf{m}}_* | \mathbf{x}_*, \text{tr-data})$ for a certain network

configuration $\mathbf{x}_*$. The transcription ODE model under these assumptions is

$$m_*(t) = \frac{b_*}{d_*} + \left(a_* - \frac{b_*}{d_*}\right) e^{-d_* t} + s_* \int_0^t \frac{1}{1 + e^{-w_{*0} - \sum_{i:x_{*i}=1} w_{*i} \log p_i(t)}} e^{-d_*(t-u)} \, \mathrm{d}u, \quad (38)$$

where $\mathbf{k}_* = \{b_*, d_*, s_*, a_*\}$ are kinetic parameters for the test gene, $\mathbf{w}_* \in \mathbb{R}^I$ the interaction weights and w_{*0} the bias parameter. When $\mathbf{x}_* = \mathbf{0}$, i.e. there are no regulating TFs, the above becomes a baseline decay model:

$$m_*(t) = \frac{b_*}{d_*} + \left(a_* - \frac{b_*}{d_*}\right) e^{-d_* t}. \quad (39)$$

Similarly to the training phase the ODE in Eq. (38) is discretised as in Eq. (12) and the likelihood function for the observations $\mathbf{m}_*$ is written as

$$p(\widetilde{\mathbf{m}}_* | \bar{\mathbf{k}}_*, \mathbf{w}_*, w_{*0}, \mathbf{p}_{\mathbf{x}_*}, \sigma_*^2) = \prod_{n=1}^{N_m} \mathcal{N}(\widetilde{m}_{*n} | m_{*n}, \sigma^2_{m,*nr} + \sigma^2_{m,*}),$$

where $\bar{\mathbf{k}}_* = \log(\mathbf{k}_*)$, $\mathbf{p}_{\mathbf{x}_*} = \{\mathbf{p}_i\}_{i:x_{*i}=1}$ and where $\sigma^2_{m,*nr}$ is the preprocessing variance and $\sigma^2_{m,*}$ is the adaptive variance. All the quantities $(\bar{\mathbf{k}}_*, \mathbf{w}_*, w_{*0}, \sigma^2_{m,*})$ are unknown parameter associated with the test gene. We place prior distributions on all these parameters which take exactly the form described in Section 3.4.1. The prior for the TF protein vectors $\mathbf{p}_{\mathbf{x}_*}$ is the posterior $p(\mathbf{p}_{\mathbf{x}_*}|\text{tr-data})$ obtained at training time. Thus, $p(\widetilde{\mathbf{m}}_*|\mathbf{x}_*, \text{tr-data})$ is written in the form

$$p(\widetilde{\mathbf{m}}_*|\mathbf{x}_*, \text{tr-data}) = \int p(\widetilde{\mathbf{m}}_* | \bar{\mathbf{k}}_*, \mathbf{w}_*, w_{*0}, \mathbf{p}_{\mathbf{x}_*}) p(\bar{\mathbf{k}}_*) p(\mathbf{w}_*) p(w_{*0}) p(\sigma^2_{m,*}) p(\mathbf{p}_{\mathbf{x}_*}|\text{tr-data}) \, \mathrm{d}\bar{\mathbf{k}}_* \, \mathrm{d}\mathbf{w}_* \, \mathrm{d}w_{*0} \, \mathrm{d}\sigma^2_{m,*} \, \mathrm{d}\mathbf{p}_{\mathbf{x}_*}.$$

By using the set of samples $\{\mathbf{p}^{(1)}_{\mathbf{x}_*}, \ldots, \mathbf{p}^{(T)}_{\mathbf{x}_*}\}$ which is a set of (dependent) draws from the posterior distribution $p(\mathbf{p}_{\mathbf{x}_*}|\text{tr-data})$, we can approximate the above by

$$p(\widetilde{\mathbf{m}}_*|\mathbf{x}_*, \text{tr-data}) \approx \int \left(\frac{1}{T} \sum_{t=1}^{T} p(\widetilde{\mathbf{m}}_* | \bar{\mathbf{k}}_*, \mathbf{w}_*, w_{*0}, \mathbf{p}^{(t)}_{\mathbf{x}_*}) \right) p(\bar{\mathbf{k}}_*) p(\mathbf{w}_*) p(w_{*0}) p(\sigma^2_{m,*}) \, \mathrm{d}\bar{\mathbf{k}}_* \, \mathrm{d}\mathbf{w}_* \, \mathrm{d}w_{*0} \, \mathrm{d}\sigma^2_{m,*}. \quad (40)$$

This quantity is hard to compute since it involves a summation of analytically intractable integrals. Each integral has the form of a marginal likelihood computation. In the next two sections, we present a fast method to approximate the above quantity that is based on sampling from the posterior distribution using an MCMC algorithm (Section 4.1.1) and then using the samples to obtain the marginal likelihood estimate (Section 4.1.2).

4.1.1 MCMC using "sample caching" and resampling

In the representation of the predictive density given by Eq. (40), one can view the term $\frac{1}{T}\sum_{t=1}^{T} p(\widetilde{\mathbf{m}}_* | \bar{\mathbf{k}}_*, \mathbf{w}_*, w_{*0}, \mathbf{p}^{(t)}_{\mathbf{x}_*})$ as a likelihood function and the remaining terms as the prior distribution over the model parameters. Under this perspective, we can run an MCMC algorithm to draw samples from the posterior $p(\bar{\mathbf{k}}_*, \mathbf{w}_*, w_{*0}, \sigma^2_{m,*} | \widetilde{m}_*, \mathbf{x}_*, \text{tr-data})$. Such samples could then be used to construct a marginal likelihood estimate. A more careful look, however, reveals that this approach is computationally very intensive and impractical. This is because the likelihood evaluation is extremely costly as we need to numerically solve T times the transcription ODE model, that is as many as the number of samples $\{P^{(1)}, \ldots, P^{(T)}\}$ obtained from the training modelling phase. This will cause the MCMC algorithm to run extremely slowly due to high computational cost when performing a single sampling step. Recall that in the *Drosophila* data set we will need to run $32 \times 6003 = 192096$ independent MCMC

algorithms which renders the genome-wide scanning procedure extremely time consuming. We should point out that a deterministic inference method, such as the Laplace approximation (Gao et al., 2008), that finds the MAP estimate for the models parameters, based on the representation in Eq. (40) will also be impractical for the same reason.

Despite these concerns, there exists an alternative view of the problem that yields a simple and computationally efficient solution. Specifically, by introducing an auxiliary discrete random variable z_*, that takes T values and has a uniform prior distribution, i.e. $\pi(z_*) = \frac{1}{T}$, $t = 1, \ldots, T$, the marginal likelihood in Eq. (40) can be obtained by marginalising out the following "data augmented" joint distribution

$$p(\widetilde{\mathbf{m}}_*, \bar{\mathbf{k}}_*, \mathbf{w}_*, w_{*0}, \sigma^2_{m,*}, z_* | \mathbf{x}_*, \text{tr-data}) = p(\widetilde{\mathbf{m}}_* | \bar{\mathbf{k}}_*, \mathbf{w}_*, w_{*0}, \mathbf{p}_{\mathbf{x}_*}^{(z_*)}) p(\bar{\mathbf{k}}_*) p(\mathbf{w}_*) p(w_{*0}) p(\sigma^2_{m,*}) \pi(z_*). \tag{41}$$

Notice that the random variable z_* simply points into the set of "cache samples" of the TF protein vectors. If we marginalise out from the above joint density all random variables, i.e. $(\bar{\mathbf{k}}_*, \mathbf{w}_*, w_{*0}, \sigma^2_{m,*}, z_*)$, then we obtain the predictive density in Eq. (40). The idea now is to construct an MCMC algorithm that moves in the augmented space by updating model parameters and the variable z_*. It turns out that such an MCMC procedure is computationally efficient.

The MCMC method takes the form of a component-wise Metropolis-Hastings algorithm (also called Metropolis-within-Gibbs) that iteratively updates the parameters according to the following steps

1. **Sample the z_*:** The relevant part of the joint density in Eq. (41) is $p(\widetilde{\mathbf{m}}_* | \bar{\mathbf{k}}_*, \mathbf{w}_*, w_{*0}, \mathbf{p}_{\mathbf{x}_*}^{(z_*)}) \pi(z_*)$. The proposal distribution $Q(z_*^{(t+1)} | z_*^{(t)})$ is chosen to have the simplest form so that it is set to be equal to the uniform prior $\pi(z_*^{t+1})$. Other more complex choices for this proposal distribution are possible, e.g. by ordering the set of samples $\{\mathbf{p}_{\mathbf{x}_*}^{(1)}, \ldots, \mathbf{p}_{\mathbf{x}_*}^{(T)}\}$ using a distance measure and then define a random walk proposal distribution that moves along the ordered set. In our experiments such more advanced proposal distributions were not needed since the simple independent uniform proposal worked and achieved reasonably high acceptance rate. The MH probability reduces to

$$\min \left\{ 1, \frac{p(\widetilde{\mathbf{m}}_* | \bar{\mathbf{k}}_*, \mathbf{w}_*, w_{*0}, \mathbf{p}_{\mathbf{x}_*}^{z_*^{(t+1)}})}{p(\widetilde{\mathbf{m}}_* | \bar{\mathbf{k}}_*, \mathbf{w}_*, w_{*0}, \mathbf{p}_{\mathbf{x}_*}^{z_*^{(t)}})} \right\}. \tag{42}$$

2. **Logarithm of the kinetic parameters $\bar{\mathbf{k}}_*$:** This is the logarithm of the actual kinetic parameters $\mathbf{k}_*$. The update is performed using a Gaussian proposal distribution as described in Section 3.5 (point 3).

3. **Interaction weights $\mathbf{w}_*$ and biases w_{*0}:** Analogously to the step 4 in Section 3.5.

4. **Noise variance $\sigma^2_{m,*}$:** This parameter is updated similarly to the step 6 in Section 3.5.

The above MCMC scheme has the advantage that each step requires only one evaluation of the transcription ODE. The output of the algorithm is a set of samples $\{z^{(t)}, \bar{\mathbf{k}}_*^{(t)}, \mathbf{w}_*^{(t)}, w_{*0}^{(t)}, (\sigma^2_{m,*})^{(t)}\}_{t=1}^T$ which involve both the variable z_* that points into "cache samples" and the model parameters. The step 1 above can be thought as a *resampling* MCMC procedure since it re-uses the samples obtained previously in the training modelling phase. This shares some similarities with particle filtering or sequential Monte Carlo techniques used in state space models. Using the set of samples, we express an approximation to the predictive density as explained in the next section. Some more implementation details of the above algorithm, such as number of burn-in and sampling iterations as well as assessing convergence are given in Appendix C.

4.1.2 The marginal likelihood estimate

There exist several methods in the literature that approximate marginal likelihoods (Newton and Raftery, 1994; Chib, 1995; Neal, 1998; Friel and Pettitt, 2008; Calderhead and Girolami, 2009). The simplest method is the harmonic mean (Newton and Raftery, 1994) that is based on the outcome of a single run of an MCMC algorithm that provides samples from the underlying posterior distribution. However, the harmonic mean gives very poor estimates in practice. More advanced methods such as the Chib's approximation (Chib, 1995) and thermodynamic integration approaches (Gelman and Meng, 1998; Neal, 1998; Friel and Pettitt, 2008; Calderhead and Girolami, 2009) are usually much more accurate but at the same time computationally more expensive. For instance, the more advanced methods are based on thermodynamic integration and require multiple parallel tempered Markov chains to run either independently or in a coupled population MCMC manner (Calderhead and Girolami, 2009; Friel and Pettitt, 2008; Neal, 1998).

For our purposes the thermodynamic integration approaches are really infeasible because the number of marginal likelihoods we need to estimate scale up to hundreds of thousands. Therefore, we will consider a simpler method and evaluate its accuracy against thermodynamic integration. Our method largely follows the formulation introduced by Chib (1995). In particular, for any probabilistic model with data $\mathbf{y}$ and parameters $\boldsymbol{\theta}$, Bayes' theorem implies the following exact expression for the marginal likelihood

$$p(\mathbf{y}) = \frac{p(\mathbf{y}|\boldsymbol{\theta})p(\boldsymbol{\theta})}{p(\boldsymbol{\theta}|\mathbf{y})}, \tag{43}$$

which holds for any value $\boldsymbol{\theta}$ of the parameters. An approximation is obtained by replacing the exact posterior value $p(\boldsymbol{\theta}|\mathbf{y})$ with an estimate $q(\boldsymbol{\theta})$. Chib suggested the use of a carefully selected parameter value $\boldsymbol{\theta}^*$ for which $q(\boldsymbol{\theta}^*)$ can closely approximate the exact posterior density value $p(\boldsymbol{\theta}^*|\mathbf{y})$. However, selecting a certain point $\boldsymbol{\theta}^*$ and constructing the approximation $q(\boldsymbol{\theta}^*)$ requires running multiple Gibbs (or block Metropolis-Hastings) samplers. This remains very expensive for our purposes. To overcome these problems we consider a simpler approach based on the outcome of a single MCMC run. An approximation of the posterior distribution can be obtained by applying a density estimation method using as data the set of samples $\{\boldsymbol{\theta}^{(1)}, \ldots, \boldsymbol{\theta}^{(T)}\}$ obtained from a single MCMC run. To remove the dependence on a certain value $\boldsymbol{\theta}^*$, we average across all samples. Specifically, by taking logarithms in Eq. (43) and replacing the exact value $p(\boldsymbol{\theta}|\mathbf{y})$ with the approximation $q(\boldsymbol{\theta})$ we obtain

$$\log p(\mathbf{y}) \approx \log p(\mathbf{y}|\boldsymbol{\theta})p(\boldsymbol{\theta}) - \log q(\boldsymbol{\theta}). \tag{44}$$

By averaging both sides with respect to the exact posterior $p(\boldsymbol{\theta}|\mathbf{y})$ we get

$$\log p(\mathbf{y}) \approx \int p(\boldsymbol{\theta}|\mathbf{y}) \log p(\mathbf{y}|\boldsymbol{\theta})p(\boldsymbol{\theta})\, \mathrm{d}\boldsymbol{\theta} - \int p(\boldsymbol{\theta}|\mathbf{y}) \log q(\boldsymbol{\theta})\, \mathrm{d}\boldsymbol{\theta}$$

and by using the samples $\{\boldsymbol{\theta}^{(1)}, \ldots, \boldsymbol{\theta}^{(T)}\}$ we approximate it using Monte Carlo as follows

$$\log p(\mathbf{y}) \approx \frac{1}{T}\sum_{t=1}^{T} \log p(\mathbf{y}|\boldsymbol{\theta}^{(t)})p(\boldsymbol{\theta}^{(t)}) - \frac{1}{T}\sum_{t=1}^{T} \log q(\boldsymbol{\theta}^{(t)}). \tag{45}$$

In practice this estimate has been shown to be much more robust (in particular averaging reduces the variance) compared to the estimator in Eq. (44) that uses a certain parameter value $\boldsymbol{\theta}^*$.

In our experiments the density function $q(\boldsymbol{\theta})$ is obtained by assuming a certain parametric form. Specifically, since $\boldsymbol{\theta} = (\bar{\mathbf{k}}_*, \mathbf{w}_*, w_{*0}, \sigma^2_{m,*}, z_*)$ and z_* is the only discrete variable while the rest are continuous variables, we

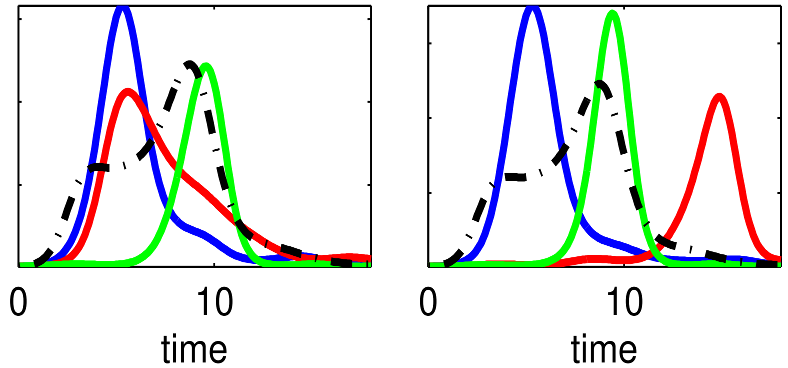

(a) (b)

Figure 1: Ground truth TF mRNA functions (ANT in blue line, BEE in red line, CAR in green line and the unknown TF in dashed black line) that generated the artificial data. Panel (a) shows the functions for the first experimental condition and panel (b) for the second experimental condition. The TF mRNAs plotted in the Figure 3 of the main paper are smoothed versions of the above functions obtained by a convolution, i.e. the solution of the linear translation ODE model.

assume the following density model

$$q(\boldsymbol{\theta}) = q(\bar{\mathbf{k}}_*, \mathbf{w}_*, w_{*0}, \sigma^2_{m,*})q(z_*).$$

Here, $q(\bar{\mathbf{k}}_*, \mathbf{w}_{\mathbf{s}_*}, w_{*0}, \sigma^2_{m,*})$ is taken to be a Gaussian distribution with a full covariance matrix and $q(z_*)$ a multinomial distribution. The parameters of these two distributions are obtained by applying maximum likelihood, i.e. maximising $\sum_{t=1}^{T} \log q(\boldsymbol{\theta}^{(t)})$, which leads to standard analytical solutions. Given the estimated density model, the marginal likelihood (recall this is the predictive density $p(\mathbf{m}_*|\mathbf{s}_*, \text{tr-data})$) is computed according to Eq. (45). Notice that this requires minimal computational effort since the first term in the sum in Eq. (45) can be precomputed during the MCMC run.

A very similar approximation to the marginal likelihood is obtained by replacing the term $-\frac{1}{T}\sum_{t=1}^{T} \log q(\boldsymbol{\theta}^{(t)})$ in Eq. (45) with the entropy of $q(\boldsymbol{\theta})$ which is computed analytically as the sum of entropy of a Gaussian distribution and the entropy of a multinomial distribution. In our experiments this yields almost indistinguishable results with the estimate in Eq. (45) and was what we used for all reported results in the main article.

To validate the accuracy of the approximation method we compared it against thermodynamic integration estimates. This was carried out in *Drosophila* data for a small set of 20 genes and all possible 32 models for each gene. Thermodynamic integration was implemented as suggested by Calderhead and Girolami (2009) using a non-uniform grid of 30 temperature values. Population MCMC was not used, that is the chains were running independently. The estimates obtained by our method were very close to the estimates obtained by the thermodynamic integration method. On the other hand, estimates obtained from Eq. (44) for carefully chosen parameter values were much less consistent with the thermodynamic integration estimates and were also varying significantly with the selected parameter value.

5 Supplemental material for the experiments

In the main paper we illustrate our methodology using mesoderm and muscle development in embryonic *Drosophila melanogaster*. We also consider an artificial example that highlights the difficulties inherent in

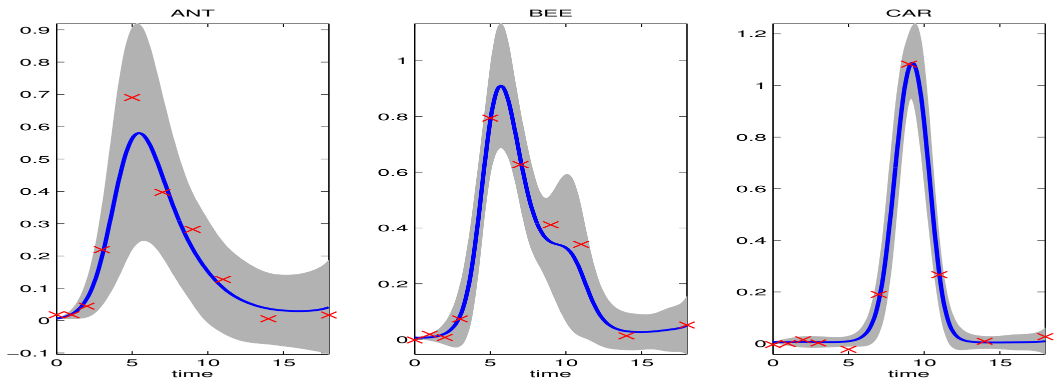

Figure 2: The predicted TF mRNAs in the artificial data using the first experimental condition. Red crosses represent the actual observations. Blue solid lines correspond to predicted mean TF mRNAs and the shaded areas show 95% credible regions around the predicted means.

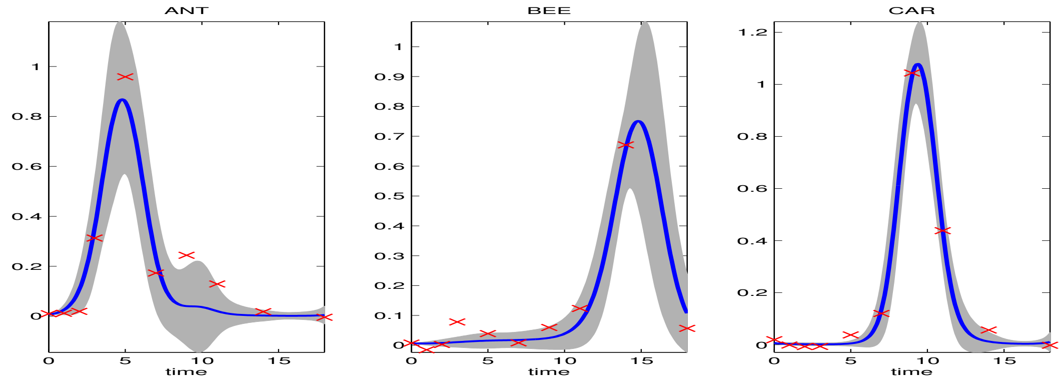

Figure 3: The predicted TF mRNAs in the artificial data using the second experimental condition. Red crosses represent the actual observations. Blue solid lines correspond to predicted mean TF mRNAs and the shaded areas show 95% credible regions around the predicted means.

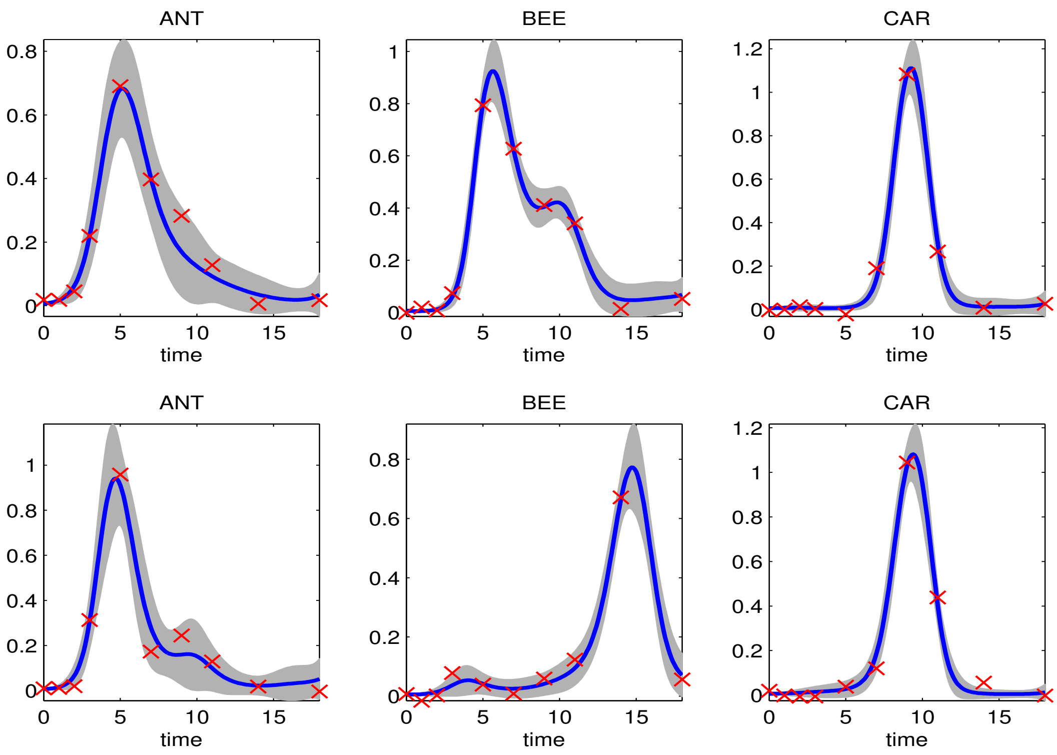

Figure 4: The predicted TF mRNAs in the artificial data using both experimental conditions. The panels in the first row correspond to the first experimental condition and panels in the second row correspond to the second experimental condition. Red crosses represent the actual observations. Blue solid lines correspond to predicted mean TF mRNAs and the shaded areas show 95% credible regions around the predicted means.

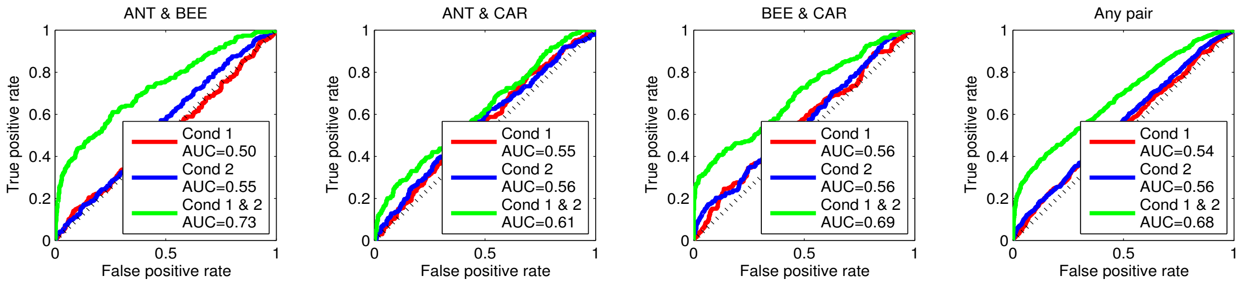

Figure 5: ROC curves for predicting the simultaneous joint regulation of pairs of two TFs in the synthetic data. Red curves show the results by using only the first experimental condition, blue curves correspond to the results when the second experimental conditions is used, green curves correspond to the results when both experimental conditions are used and the diagonal dotted corresponds to random prediction. The first three plots from the left show the ROC curves for predicting the pair-links associated with the three possible pairs of TFs. The last plot shows the overall performance, i.e. for predicting any pair-link.

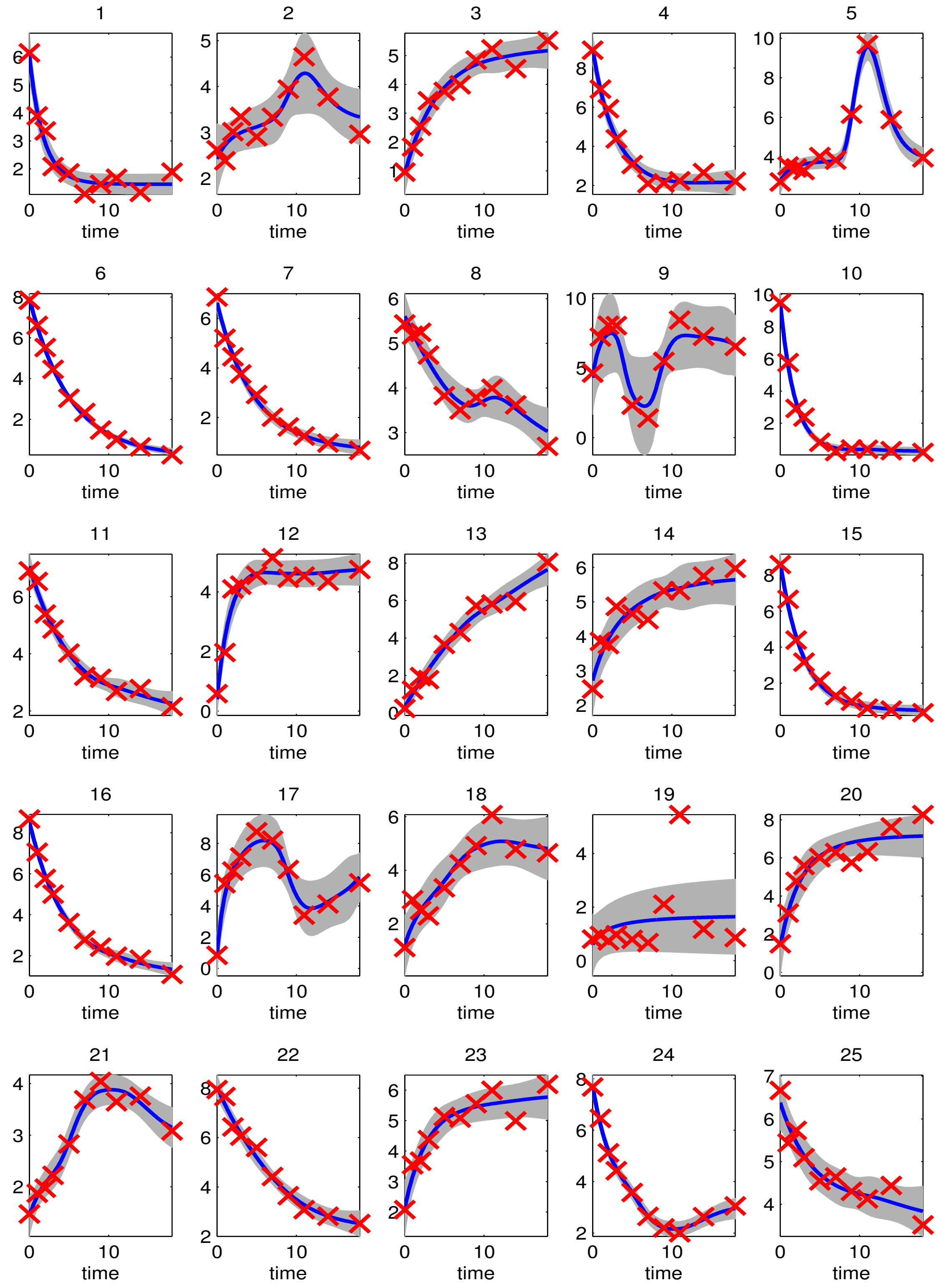

Figure 6: Examples of fitting the training genes in artificial data by using only the first experimental condition. The first 25 genes (out of 30) are shown and each subplot corresponds to a certain gene. Red crosses represent the actual observations. Blue solid lines correspond to mean predictions of the mRNA functions and the shaded areas show 95% credible regions around the predicted means.

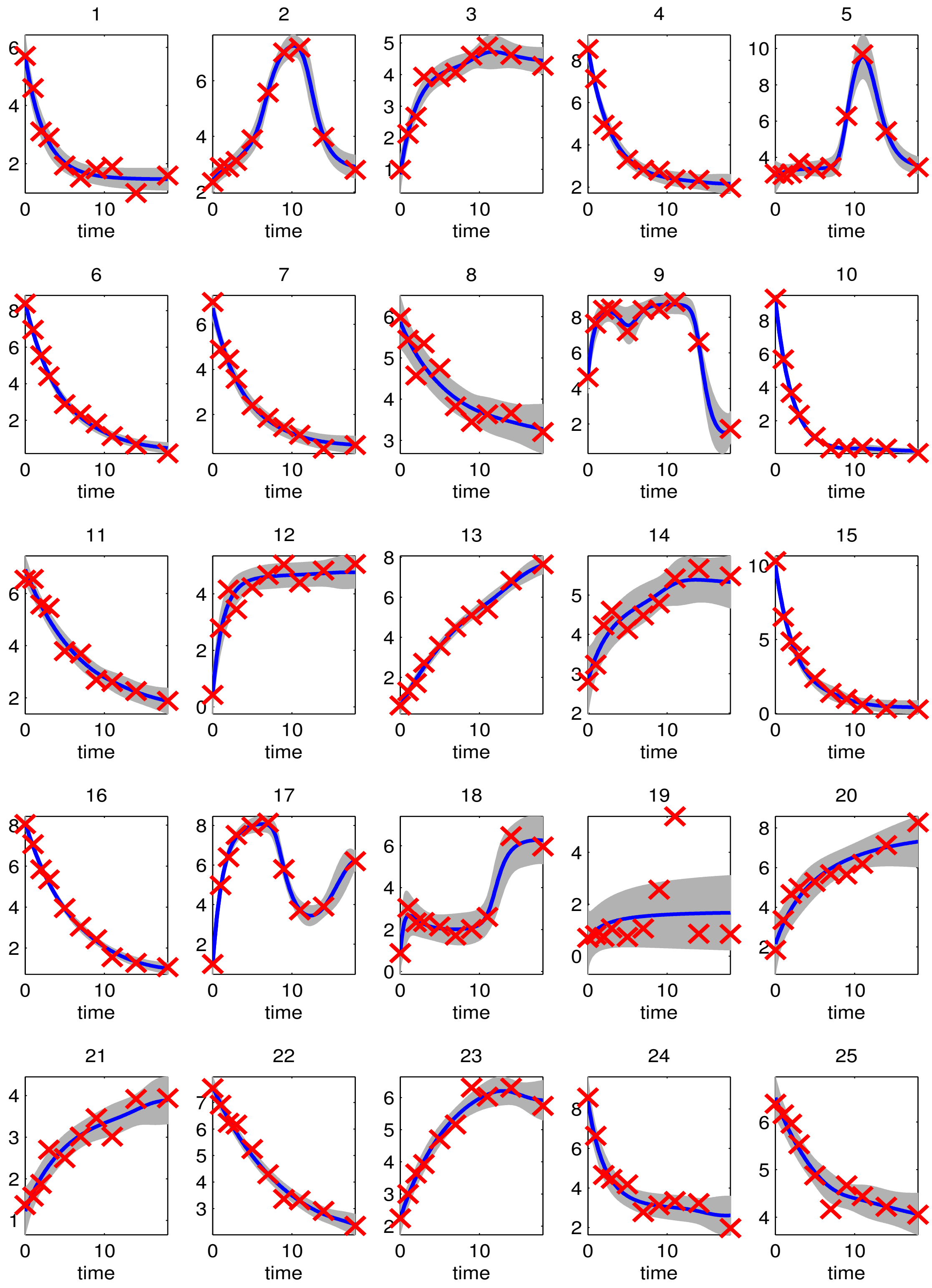

Figure 7: Examples of fitting the training genes in artificial data by using only the second experimental condition. The first 25 genes (out of 30) are shown and each subplot corresponds to a certain gene. Red crosses represent the actual observations. Blue solid lines correspond to mean predictions of the mRNA functions and the shaded areas show 95% credible regions around the predicted means.

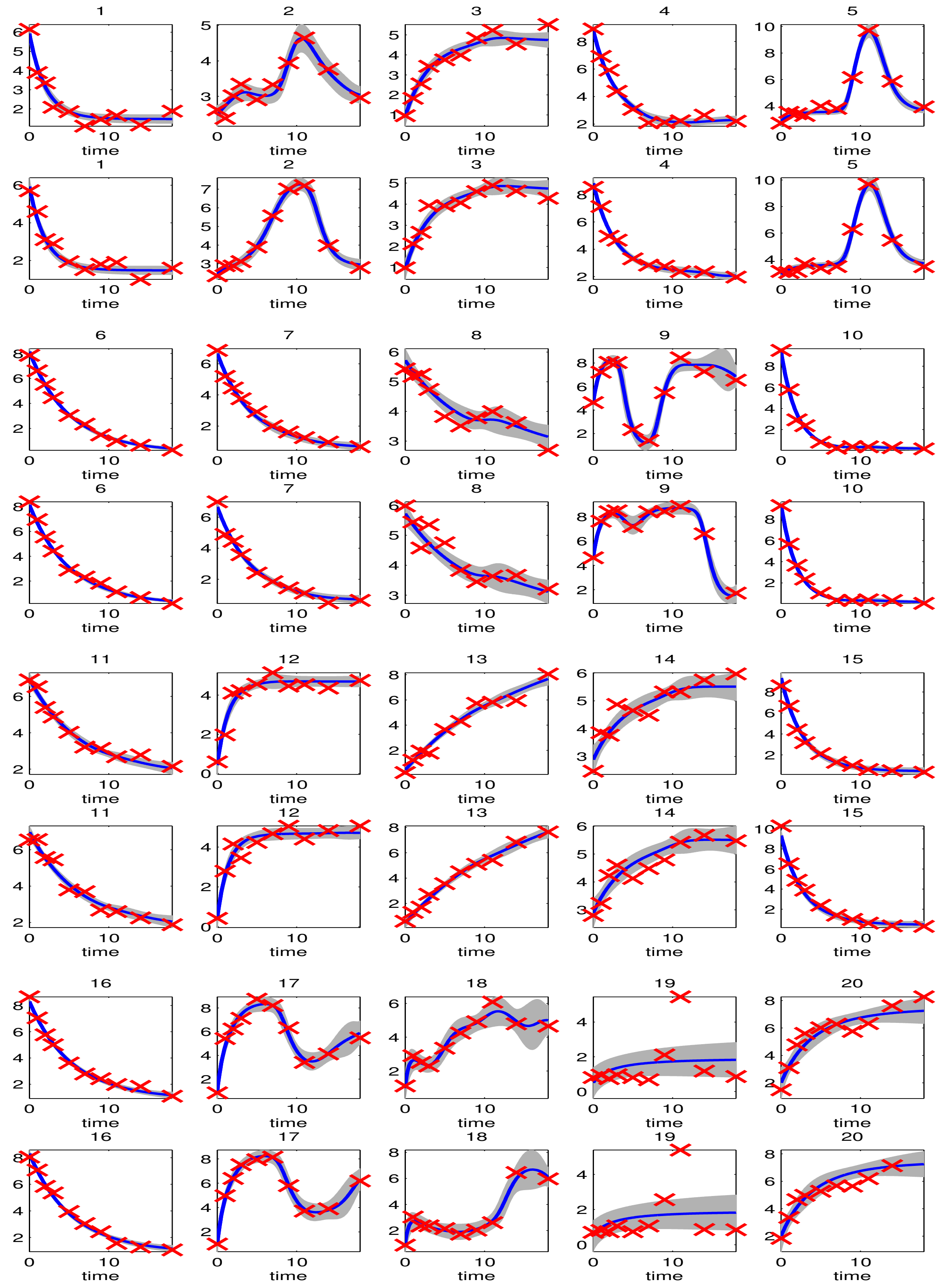

Figure 8: Examples of fitting the training genes in the artificial data with both experimental conditions. Fits for 20 genes and the associated two experimental conditions are shown. First and second row show the fits for the genes 1,2,3,4 and 5 so that the panels in the first row correspond to the first experimental condition while those of the second row correspond to the second experimental condition. The fits for the remaing genes are presented in an exactly analogous format in the remaining rows of the figure. For all plots, blue solid lines correspond to mean predictions of the mRNA functions and the shaded area shows 95% credible regions.

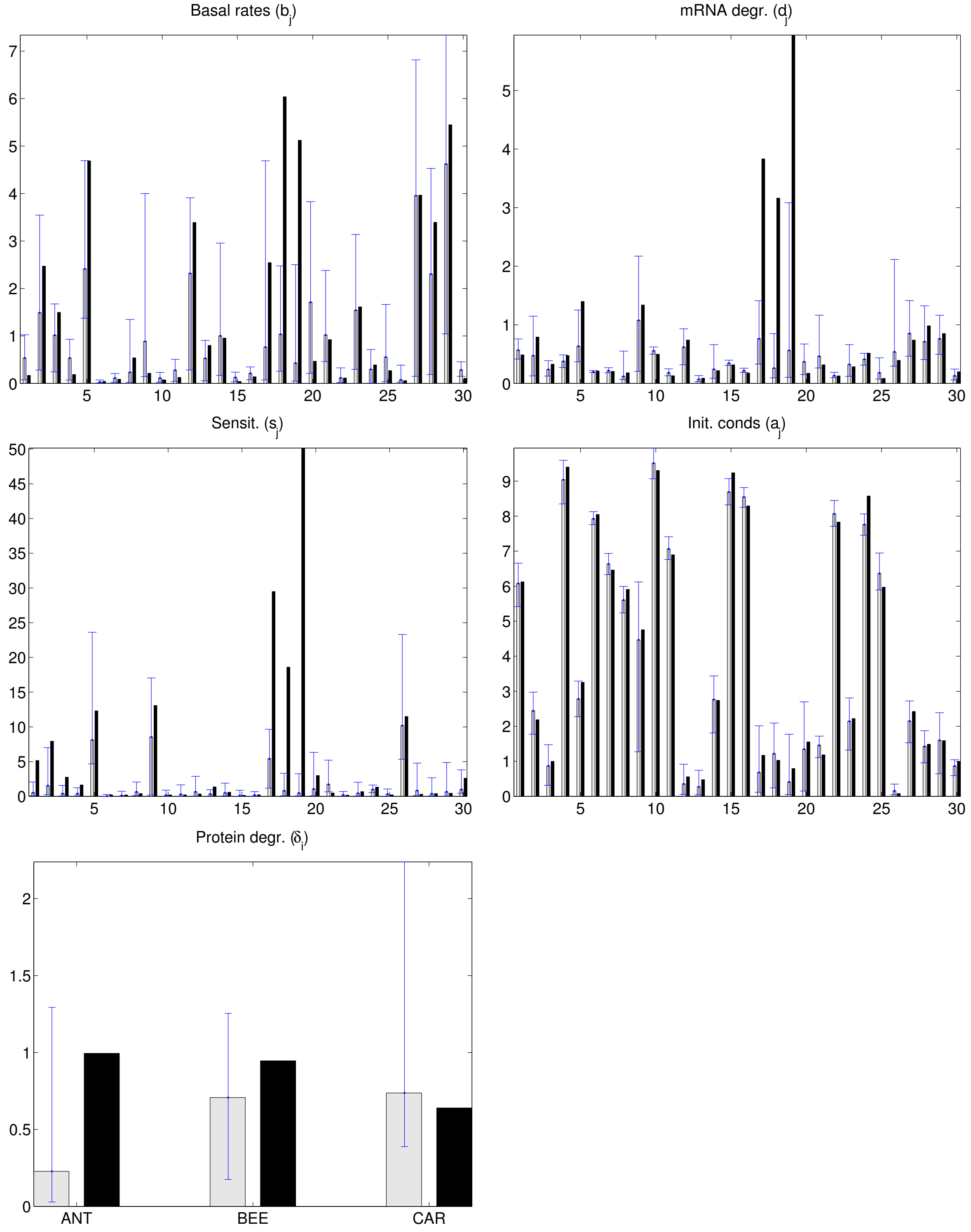

Figure 9: Transcription ODE kinetic parameters (first two rows) and translation ODE degradation rates (last row) in the artificial data using only the first experimental condition. The bright colour bars show the estimated values with 95% credible regions (estimated using percentiles), while black bars show the ground-truth values. In the first four plots (from top) the horizontal axis has 30 different pairs of bars where each pair corresponds to each training gene. The last plot has three pairs of bars that show the estimated degradation rates for the TFs (ANT, BEE and CAR).

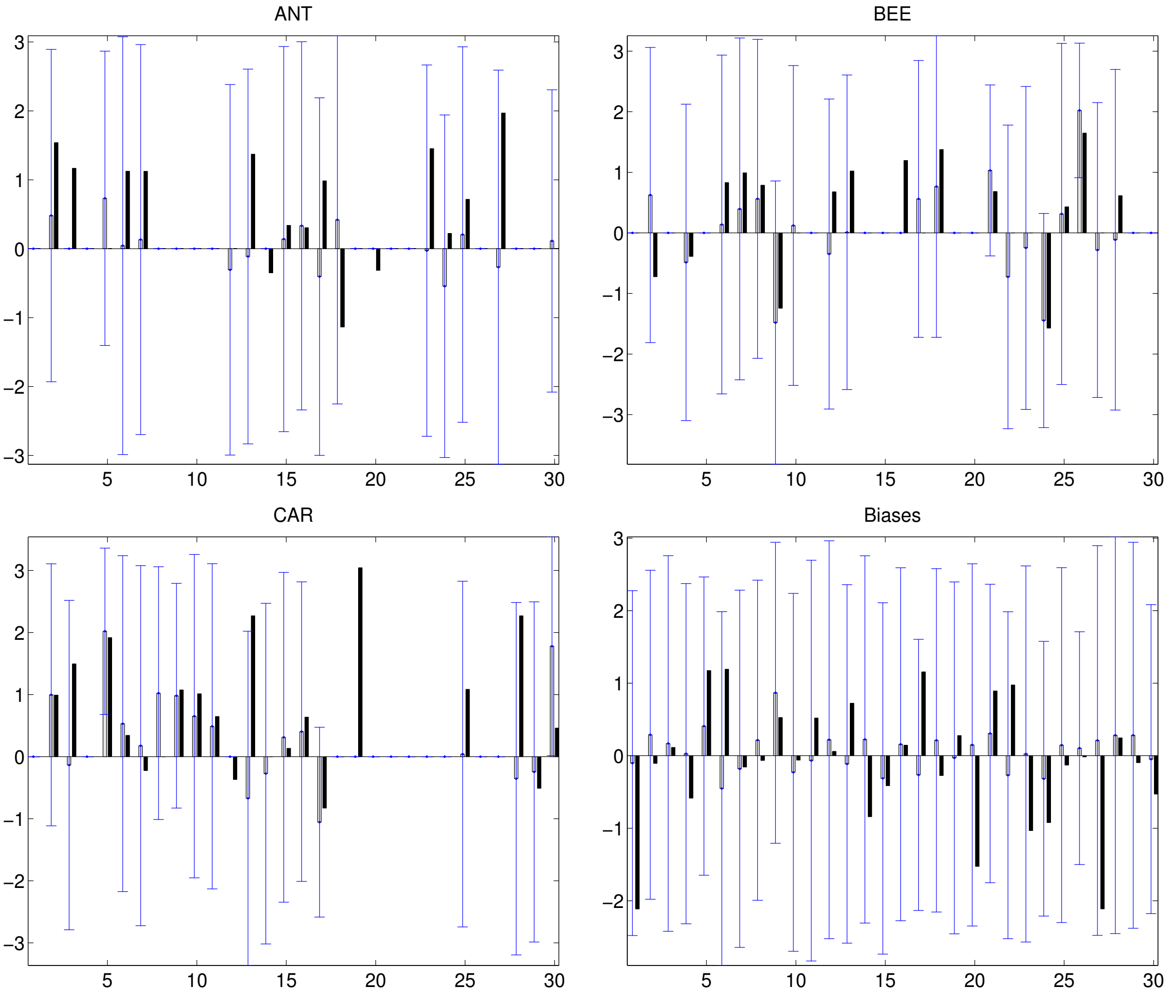

Figure 10: Interaction weights and biases in the artificial data using only the first experimental condition. The bright colour bars show the estimated values with 95% credible regions (estimated using percentiles), while black bars show the ground-truth values. In each plot the horizontal axis has 30 different pairs of bars where each pair corresponds to each training gene.

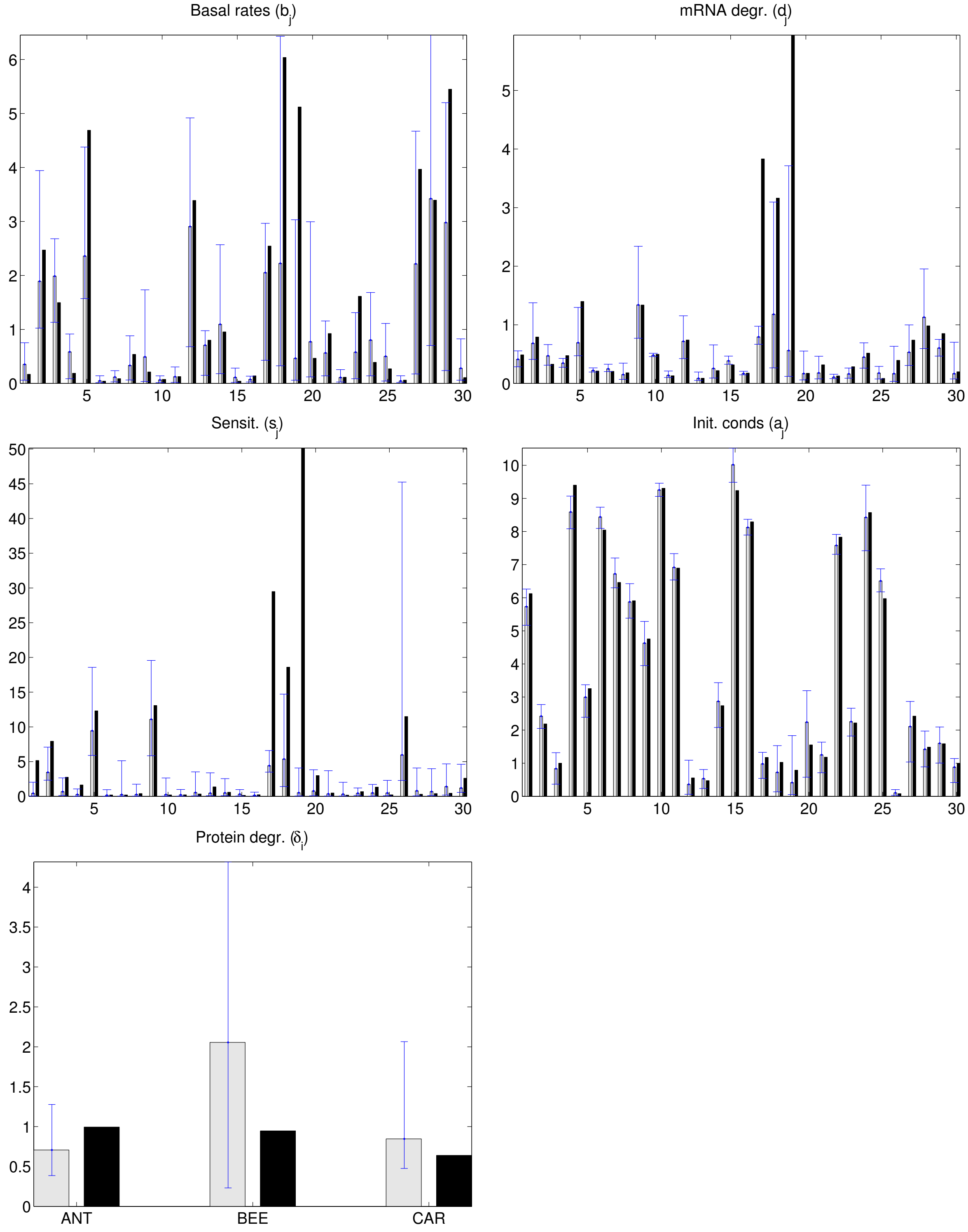

Figure 11: Transcription ODE kinetic parameters (first two rows) and translation ODE degradation rates (last row) in the artificial data using only the second experimental condition. The bright colour bars show the estimated values with 95% credible regions (estimated using percentiles), while black bars show the ground-truth values. In the first four plots (from top) the horizontal axis has 30 different pairs of bars where each pair corresponds to each training gene. The last plot has three pairs of bars that show the estimated degradation rates for the TFs (ANT, BEE and CAR).

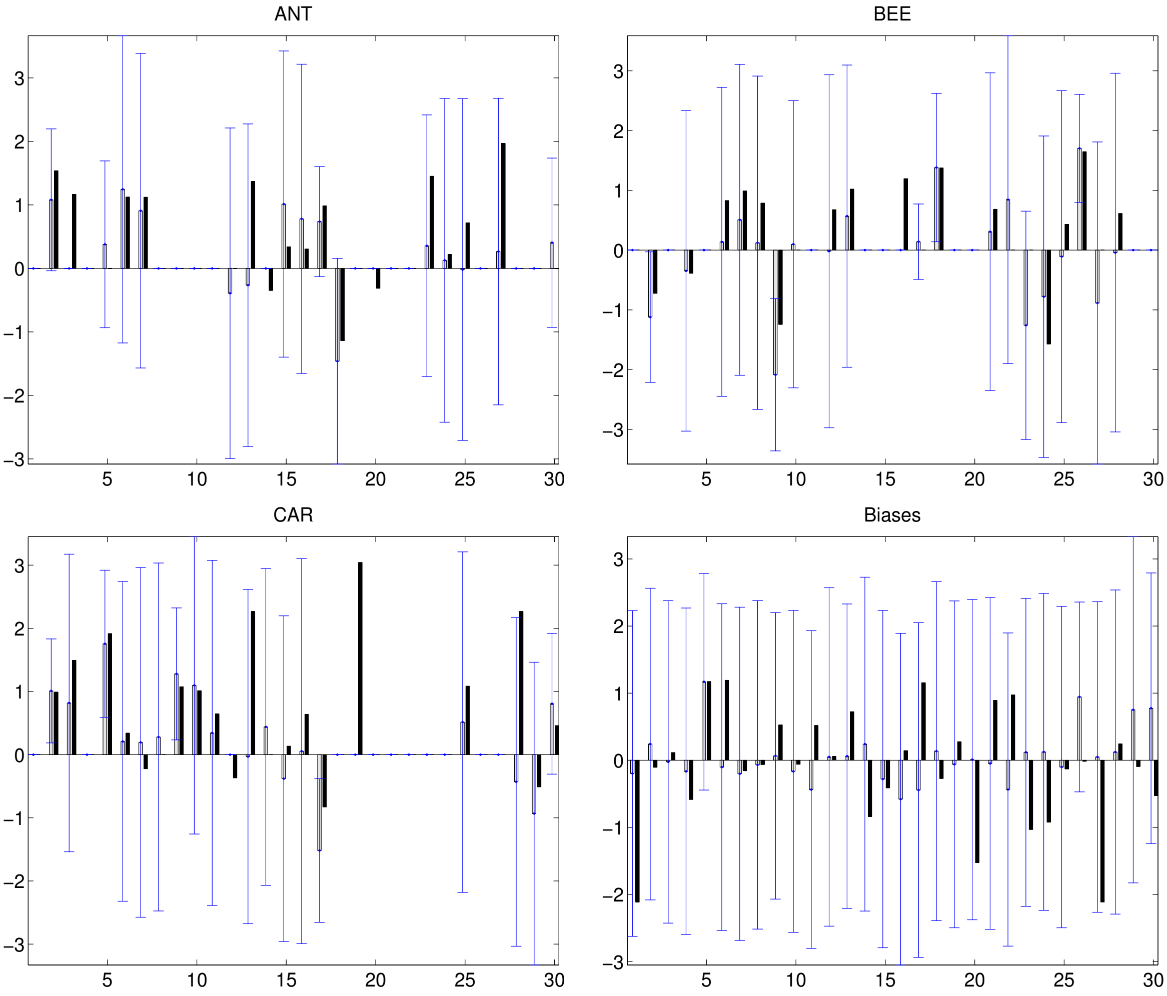

Figure 12: Interaction weights and biases in the artificial data using only the second experimental condition. The bright colour bars show the estimated values with 95% credible regions (estimated using percentiles), while black bars show the ground-truth values. In each plot the horizontal axis has 30 different pairs of bars where each pair corresponds to each training gene.

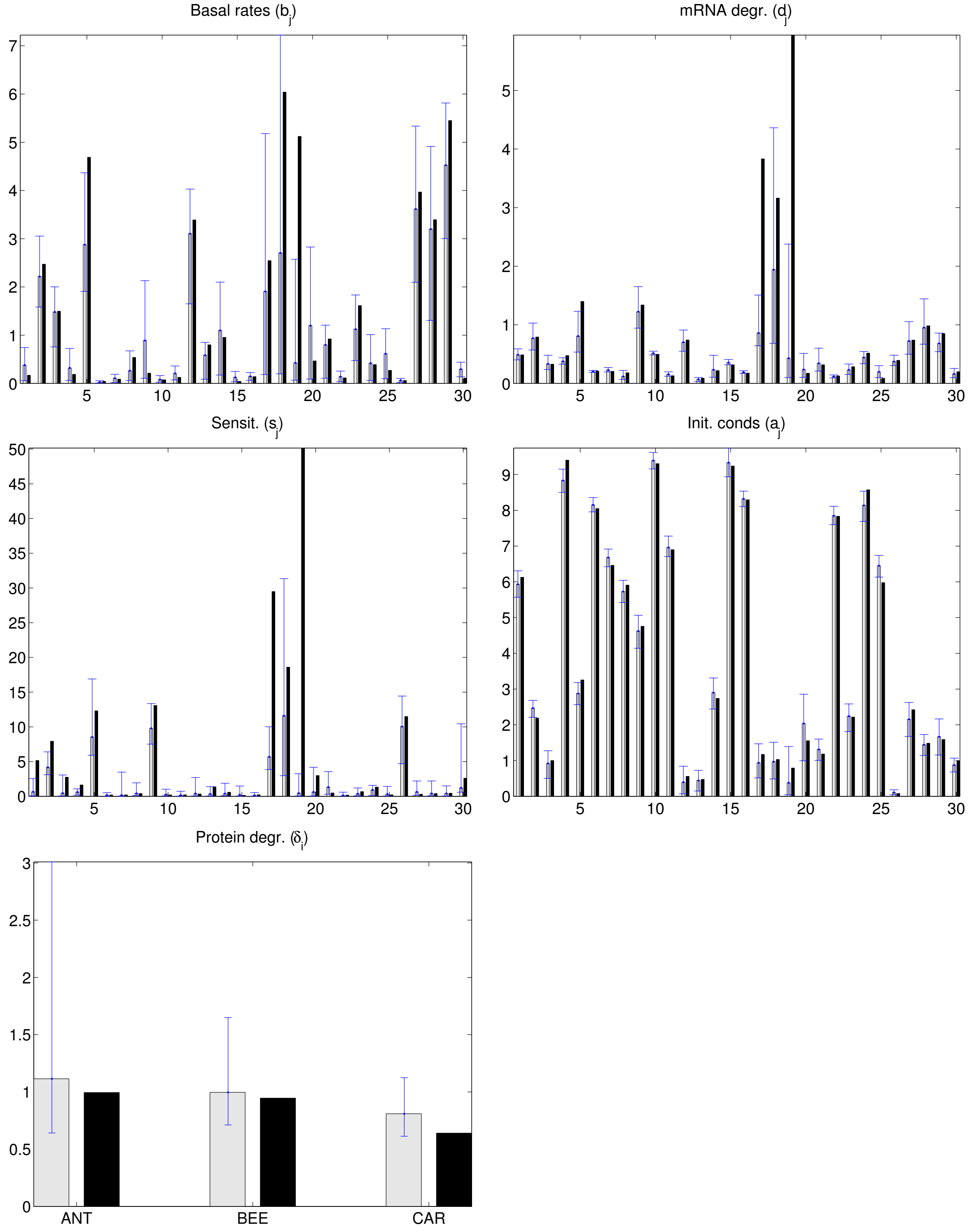

Figure 13: Transcription ODE kinetics parameters (first two rows) and translation ODE degradation rates (last row) in the artificial data using both experimental conditions. The bright colour bars show the estimated values with 95% credible regions (estimated using percentiles), while black bars show the ground-truth values. In the first four plots (from top) the horizontal axis has 30 different pairs of bars where each pair corresponds to each training gene. The last plot has three pairs of bars that show the estimated degradation rates for the TFs (ANT, BEE and CAR).

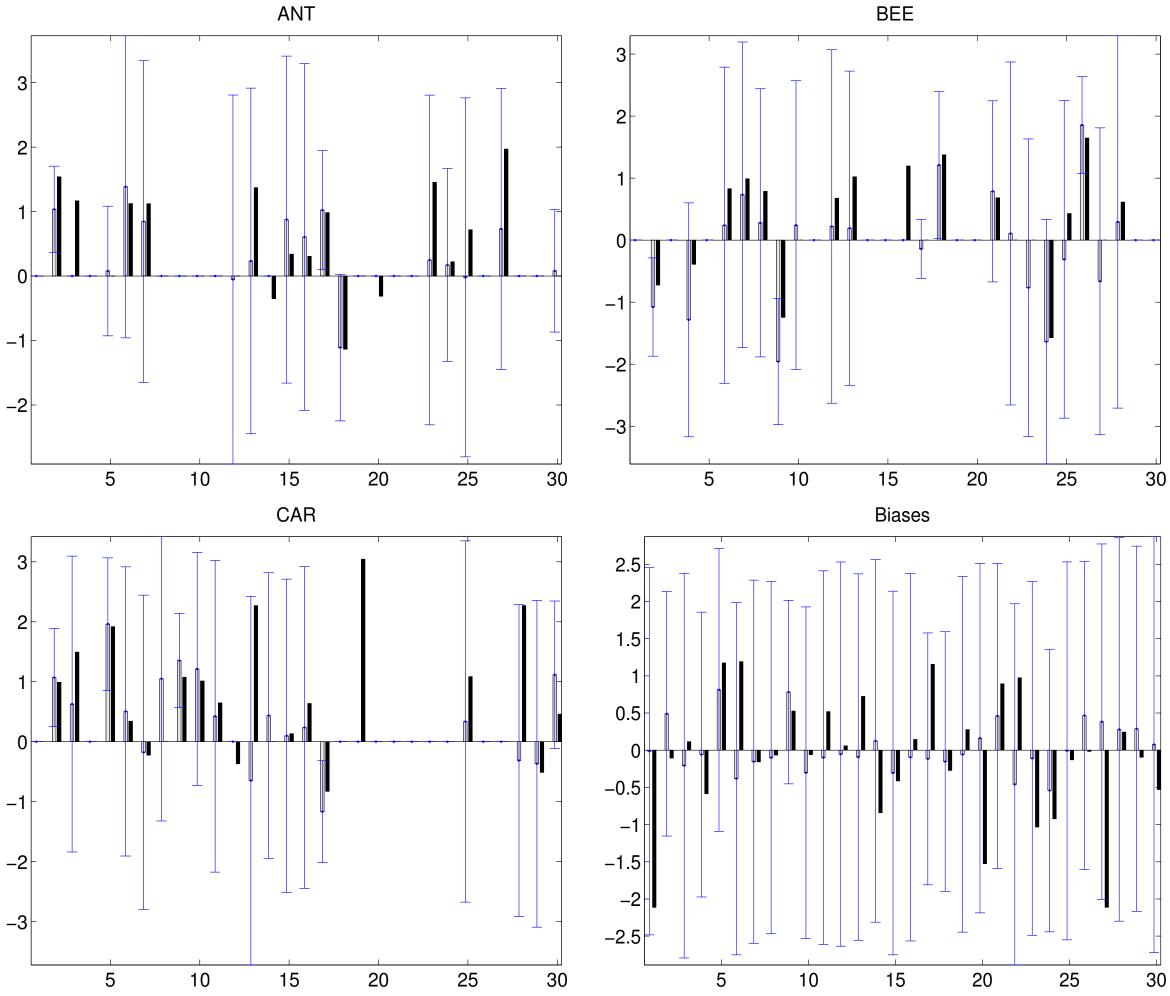

Figure 14: Interaction weights and biases in the artificial data using both experimental conditions. The bright colour bars show the estimated values with 95% credible regions (estimated using percentiles), while black bars show the ground-truth values. In each plot the horizontal axis has 30 different pairs of bars where each pair corresponds to each training gene.

inference of transcription networks directly from data. This section is meant to provide supplemental information with regard to the section *Results* of the main paper.

5.1 Synthetic data

We consider an artificial gene network involving *four* transcription factors: ANT, BEE, CAR and UNK. As explained in the main paper, when modelling the data we only consider *three* of these transcription factors: ANT, BEE and CAR. This reflects a realistic scenario where there is an unacknowledged confounding transcription factor (UNK) affecting our system. We simulated two experimental conditions. In our first experimental condition there is considerable overlap between the mRNA profiles of the TFs. In both experimental conditions there is considerable overlap between UNK and the three known TFs. In the first experimental condition ANT and BEE also exhibit considerable overlap making them extremely difficult to disambiguate. In the second experimental condition the overlap of BEE with ANT is far less.

Plots of the TF profiles that generated the data are given in the main paper. Plots of the corresponding ground truth TF mRNA functions that deterministically define these TF profiles through the translation ODE model are shown in Figure 1. When using only the first experimental condition and the 30 training genes, the fitted TF mRNA data for ANT, BEE, CAR are shown in Figure 2, while when using only the second experimental condition the fitted TF mRNA data for ANT, BEE, CAR are shown in Figure 3. The corresponding TF mRNA fits associated with using simultaneously both experimental conditions are shown in Figure 4.

Similarly to the single-TF predictions shown in Figure 4 of the main paper, Figure 5 displays the results for predicting a simultaneous or joint regulation of the target gene by a pair of two TFs. In other words, a pair-link associated with two TFs in the transcription gene network is defined to be active/present if both TFs jointly regulate the target gene. We used the model to predict such pair-links for all three possible pairs of the TFs. Red lines show ROC curves corresponding to the first experimental condition, blue lines show ROC curves corresponding to the second experimental condition, while green lines are ROC curves corresponding to the use of both experimental conditions. Clearly, when using data from both experimental conditions the performance improves significantly. On the other hand when using only the first experimental condition the performance is poor, while for only the second experimental condition the performance is slightly better compared to using only the first one.

Examples of fitting the training genes when using only the first, only the second and both experimental conditions are shown in Figure 6, 7 and 8 respectively. Further plots on the estimated model parameters in the training phase are shown in Figures 9–14.

5.2 *Drosophila* data

In this section we provide additional information and plots regarding the application of our method to the *Drosophila* embryogenesis in wild-type embryos (Tomancak et al., 2002). In these data three replicates of the time series data are available. Figure 15 shows the inferred profiles for all five TFs and all three replicas together with the corresponding predicted TF mRNA profiles.

Figure 16 shows the single-TF regulator rankings computed separately for each TF. Similarly, Figure 17 shows separate TF-pair regulator rankings for all possible pairs of TFs. The figures show considerable diversity in prediction performance for different TFs. In the single-TF rankings, the restricted models are far superior for TIN and BIN while for others the situation is more even. In TF-pair rankings there is significant variation in which method is the best.

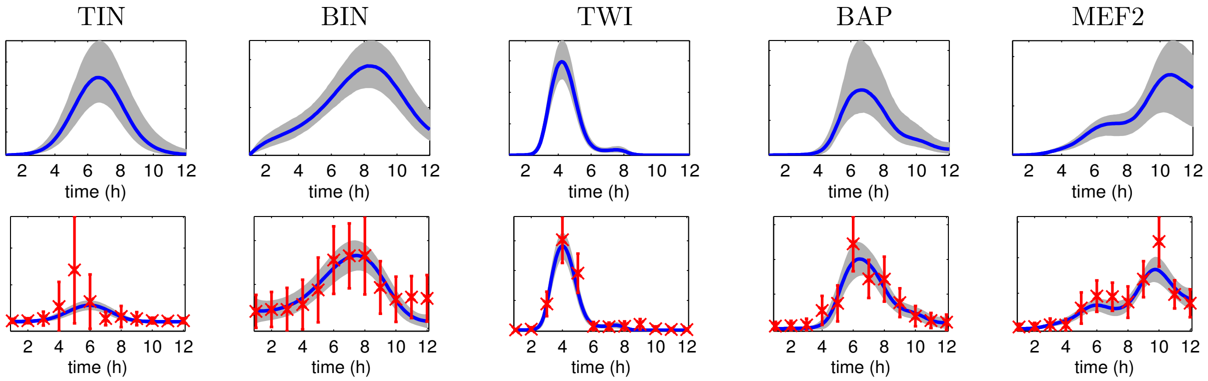

(a) First replica

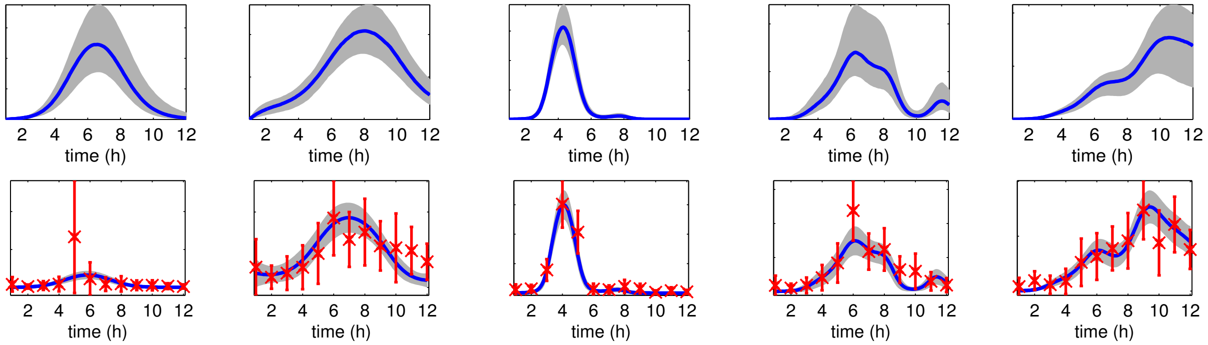

(b) Second replica

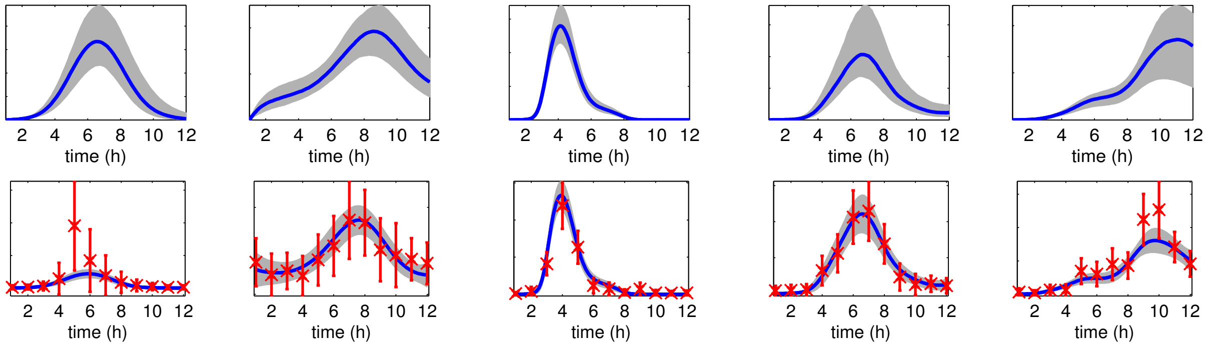

(c) Third replica

Figure 15: The estimated TF activities from the training modelling phase in *Drosophila* data together with the corresponding predicted TF mRNAs. The figure has three sub-figures (a,b,c) each associated with a single replica of the time series. For a certain sub-figure, the five plots is the first row display the estimated TFs of the corresponding replica so that each blue solid line represents an estimated mean TF activity and the shaded area represents 95% credible regions around the mean. The five plots in the second row of a certain sub-figure display the predicted TF mRNAs (blue solid lines and shaded areas) together with the observed data represened by red crosses (means) and vertical lines (two-standard deviations around the means provided by the microarray preprocessing stage).

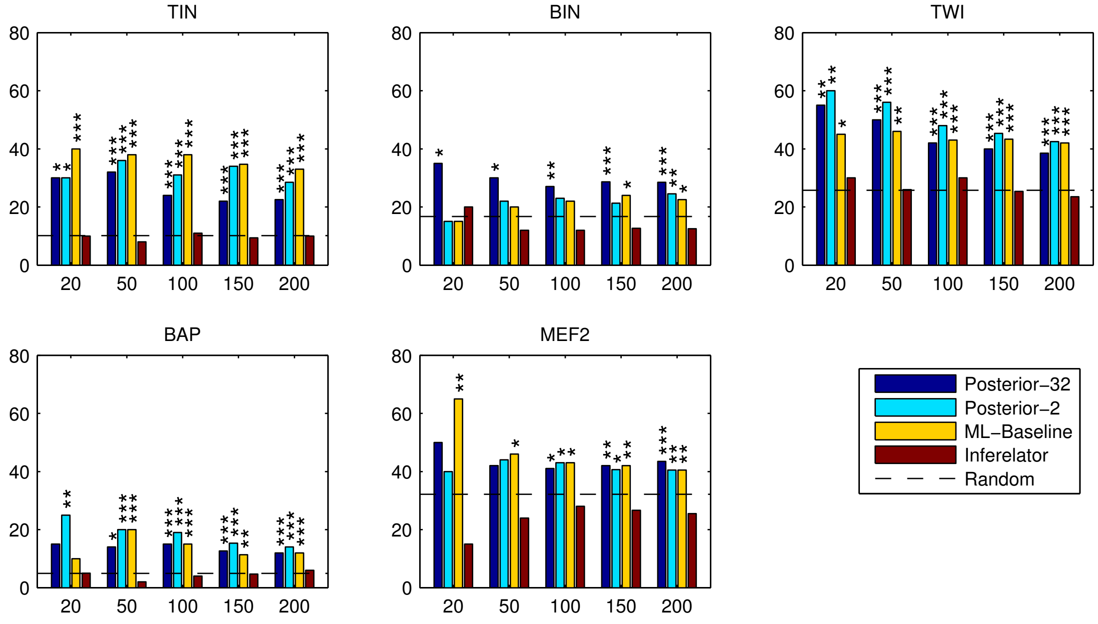

Figure 16: ChIP enrichment for the ranked putative targets. Similar to Figure 7(a) (main paper) but broken down to individual TFs.

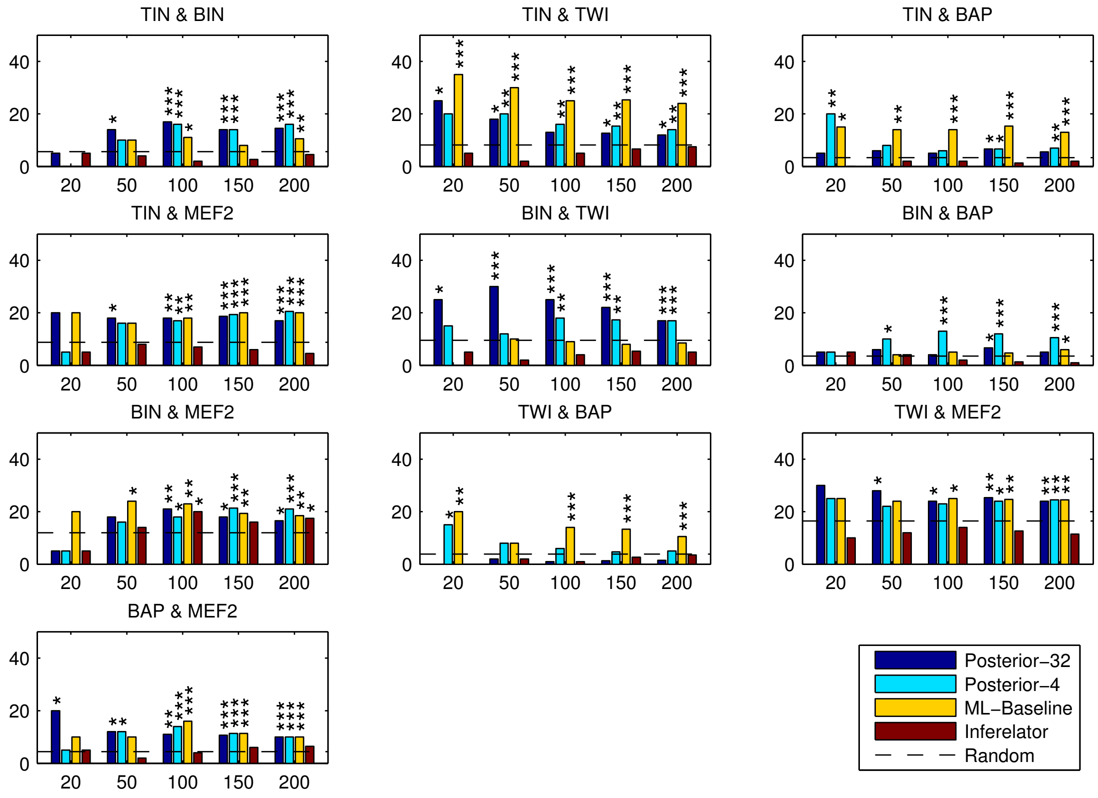

Figure 17: Enrichment of binding of predicted regulator TFs similar to Figure 7(b) (main paper) broken down to all ten possible combinations of TF pairs.

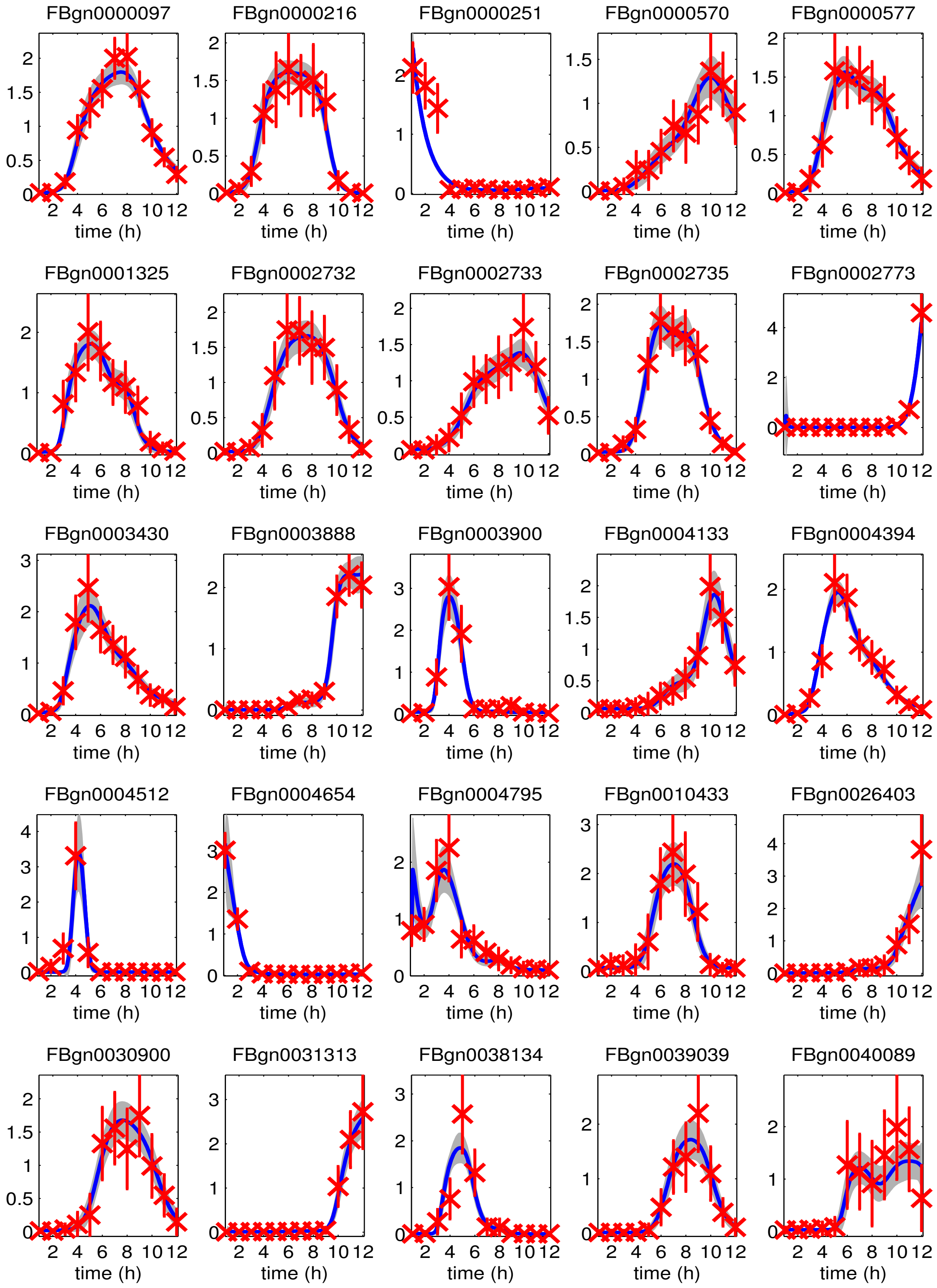

Figure 18: Examples of fitting the 25 training genes in the *Drosophila* data. For all plots, blue solid lines correspond to mean predictions of the mRNA functions and the shaded area shows 95% credible regions. Observed data are represented by red crosses (means) and vertical lines (two-standard deviations around the means provided by the microarray preprocessing stage). All the displayed plots correspond to the first replica of the time series data.

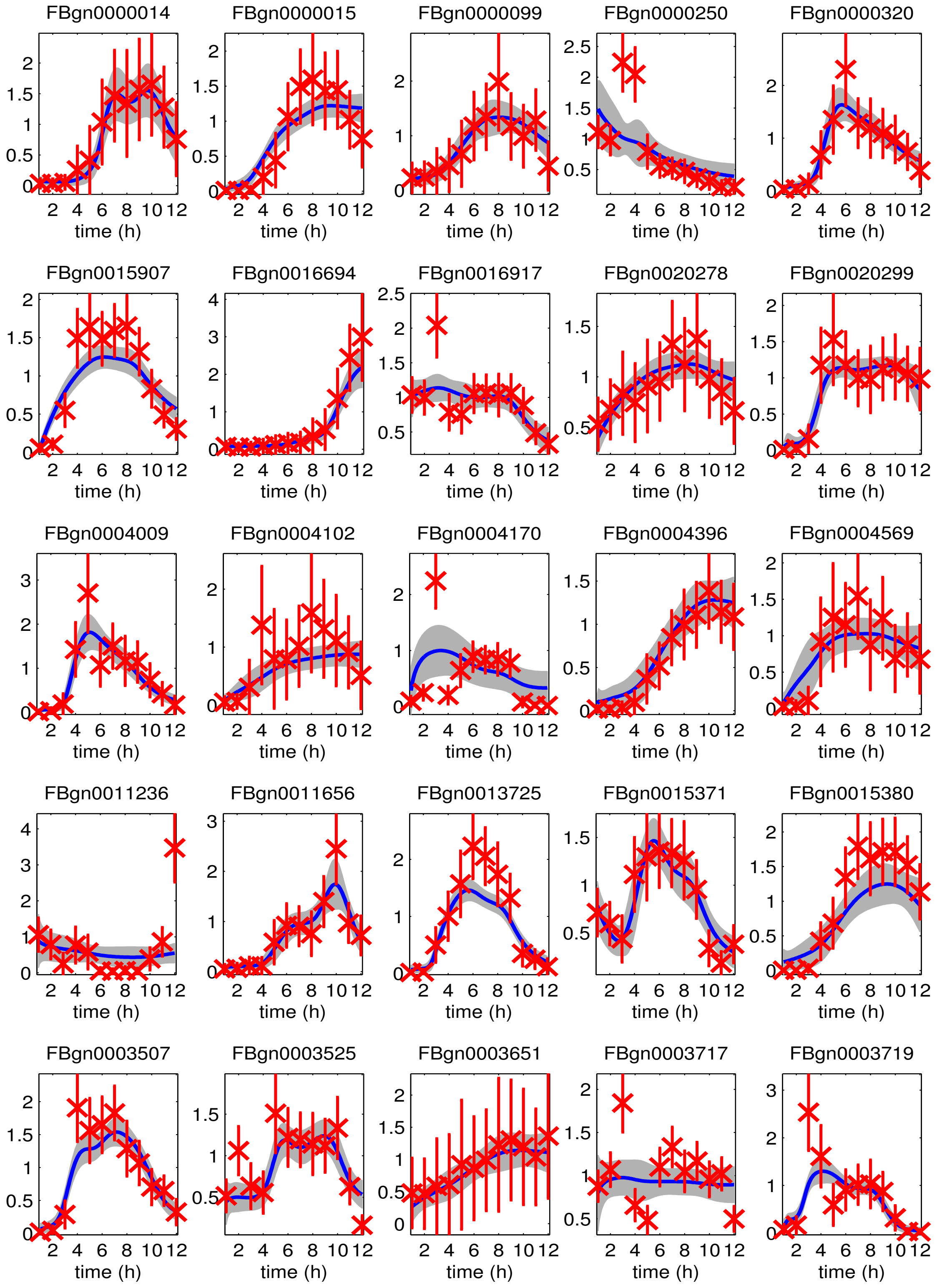

Figure 19: Models fits for a selection of genes from the set of 67 genes which were excluded from the original 92 training genes (see Figure 18) using the robustified model. The fits are displayed in exactly the same format used in Figure 18 and all displayed plots correspond to the first replica of the time series data.

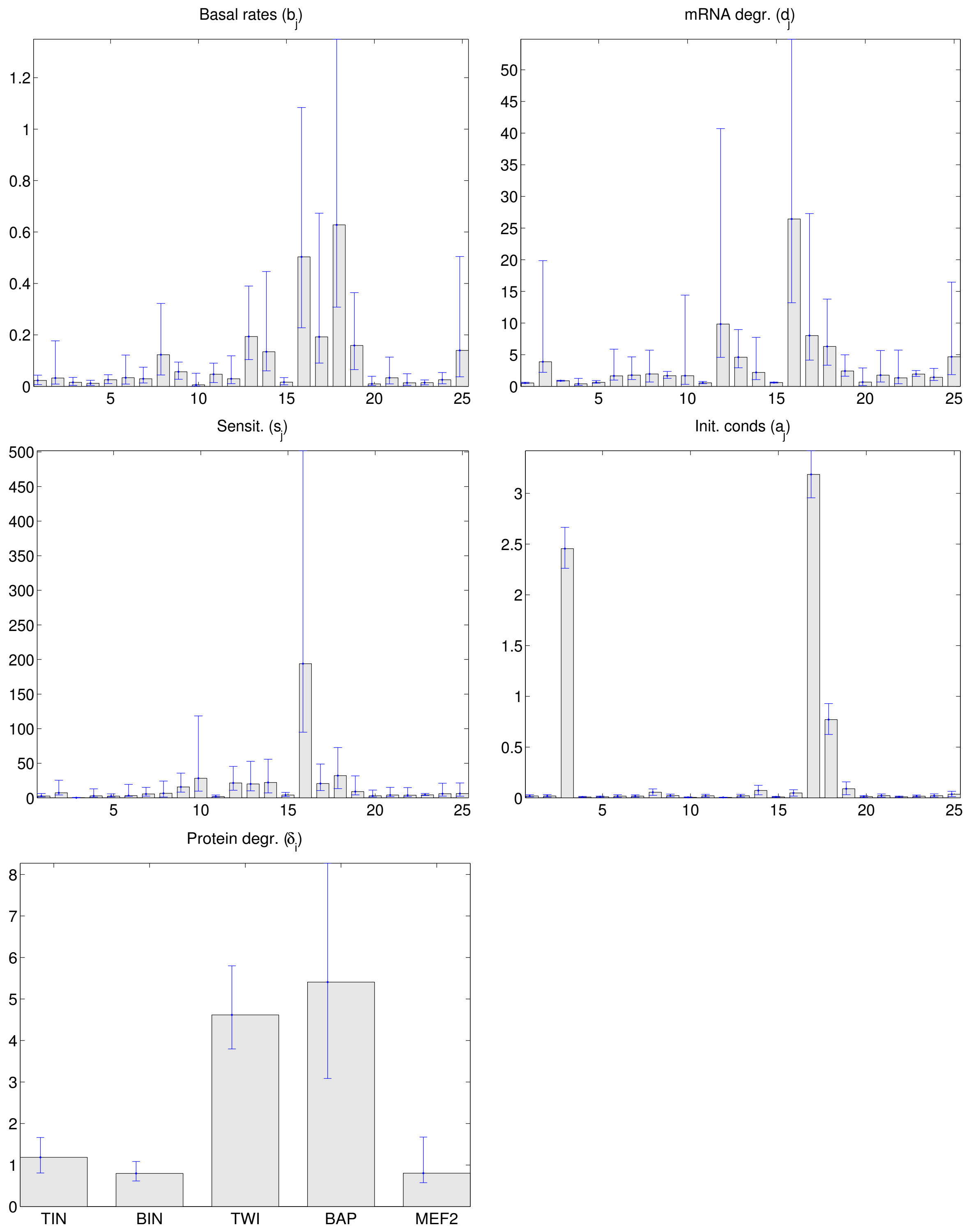

Figure 20: Transcription ODE kinetics parameters (first two rows) and translation ODE degradation rates (last row) in *Drosophila* training genes. The bright colour bars show the estimated values with 95% credible regions estimated using percentiles. In the first four plots the horizontal axis has 25 different bars where each corresponds to each training gene. The last plot has five bars that show the estimated protein degradation rates for the five TFs.

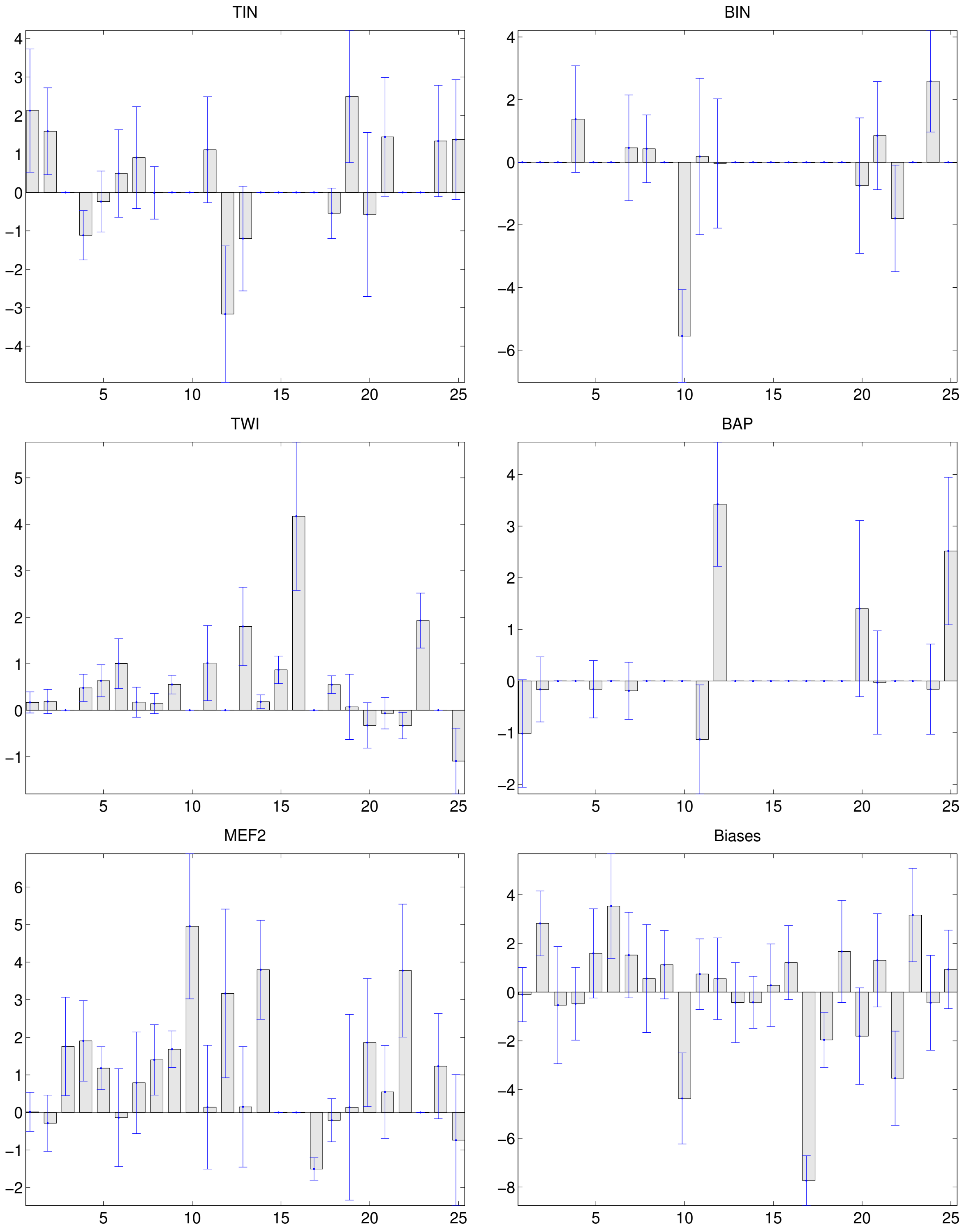

Figure 21: Interaction weights and biases in the *Drosophila* data. The bright colour bars show the estimated values with 95% credible regions estimated using percentiles. In each plot the horizontal axis has 25 different bars where each corresponds to each training gene.

Plots for fitting the mRNA data for all 25 genes used in the training phase are shown in Figure 18. Recall that these genes were obtained by a larger set of 92 genes and after filtering out the genes that were not predicted well by the model. This filtering was carried out by applying firstly the training phase with likelihood functions having both preprocessing noise variances and the adaptive variances, i.e. exactly as explained in Section 3. Then, genes with inferred adaptive variances roughly larger than 0.01 were excluded. Since the estimation involved the collection of posterior samples, the threshold 0.01 was compared to the value $\bar{\sigma}^2_{m,j}$ for which $P(\sigma^2_{m,j} \in [0, \bar{\sigma}^2_{m,j}]) = 0.95$. $\bar{\sigma}^2_{m,j}$ was computed empirically as the 95% percentile of the posterior samples. After this filtering procedure the training phase was repeated for only the 25 selected genes and using likelihood functions having only the preprocessing variances. The estimated training phase model parameters are displayed in Figures 20–21.

A Sampling the GP latent functions

In Section 3.5, the step for sampling the GP vector $\bar{\mathbf{f}}_i$ requires a MH update where the relevant part of the joint probability density is

$$p(\widetilde{M}|\bar{\mathbf{f}}_i, \{\bar{\mathbf{f}}_j\}_{j \neq i}, \bar{K}, W, \mathbf{w}_0, \bar{\boldsymbol{\delta}}, \Sigma_m) p(\widetilde{\mathbf{f}}_i|\bar{\mathbf{f}}_i, \sigma^2_{f,i}) p(\bar{\mathbf{f}}_i|v_i, \ell^2_i). \tag{46}$$

The proposal distribution takes the form

$$Q(\bar{\mathbf{f}}'_i|\bar{\mathbf{f}}_i) = H(\bar{\mathbf{f}}', \bar{\mathbf{f}}) p(\bar{\mathbf{f}}'_i|v_i, \ell^2_i),$$

where $p(\bar{\mathbf{f}}'_i|v_i, \ell^2_i)$ is the GP prior that appears also in the part of the joint density and $H(\bar{\mathbf{f}}', \bar{\mathbf{f}})$ is a symmetric and log-quadratic function with respect to its arguments. $H(\bar{\mathbf{f}}', \bar{\mathbf{f}})$ is set to incorporate some properties of the non-Gaussian likelihood, that is the term $\mathcal{L}(\bar{\mathbf{f}}_i) = p(\widetilde{M}|\bar{\mathbf{f}}_i, \{\bar{\mathbf{f}}_j\}_{j \neq i}, \bar{K}, W, \mathbf{w}_0, \bar{\boldsymbol{\delta}}, \Sigma_m) p(\widetilde{\mathbf{f}}_i|\bar{\mathbf{f}}_i, \sigma^2_{f,i})$ viewed for simplicity as a function of $\bar{\mathbf{f}}_i$ which is the variable we wish to sample. Auxiliary variables can be employed for the construction of $H(\bar{\mathbf{f}}', \bar{\mathbf{f}})$. The idea is to approximate the non-Gaussian likelihood $\mathcal{L}(\bar{\mathbf{f}}_i)$ by an auxiliary Gaussian likelihood $p(\mathbf{z}_i|\bar{\mathbf{f}}_i)$ where $\mathbf{z}_i$ are auxiliary variables that can be regarded as auxiliary or pseudo data. This auxiliary likelihood is chosen to be $p(\mathbf{z}_i|\bar{\mathbf{f}}_i) = \mathcal{N}(\mathbf{z}_i|\bar{\mathbf{f}}_i, h_{f_i} I)$ which says that $\mathbf{z}_i$ is a noisy version of $\bar{\mathbf{f}}_i$. The sampling scheme iterates between updating $\mathbf{z}_i$ and $\bar{\mathbf{f}}_i$ according to

1. $\mathbf{z}_i \sim p(\mathbf{z}_i|\bar{\mathbf{f}}_i) = \mathcal{N}(\mathbf{z}_i|\bar{\mathbf{f}}_i, h_{f_i} I)$ (Gibbs step),
2. $\bar{\mathbf{f}}'_i \sim Q(\bar{\mathbf{f}}'_i|\mathbf{z}_i) = \frac{1}{\mathcal{Z}(\mathbf{z}_i)} \mathcal{N}(\mathbf{z}_i|\bar{\mathbf{f}}'_i, h_{f_i} I) p(\bar{\mathbf{f}}'_i|v_i, \ell^2_i)$ (accept/reject using MH)

One way to understand this iteration is to view it as sampling from an augmented posterior distribution over $\mathbf{z}_i$ and $\bar{\mathbf{f}}_i$ associated with the following augmented probability density function

$$\mathcal{L}(\bar{\mathbf{f}}_i) p(\mathbf{z}_i|\bar{\mathbf{f}}_i) p(\bar{\mathbf{f}}'_i|v_i, \ell^2_i). \tag{47}$$

A full sweep over both steps 1 and 2 leaves the conditional posterior distribution over $\bar{\mathbf{f}}_i$ (in the original unaugmented model) invariant and constructs a Markov transition kernel having the following proposal distribution

$$\begin{aligned} Q(\bar{\mathbf{f}}'_i|\bar{\mathbf{f}}_i) &= \int Q(\bar{\mathbf{f}}'_i|\mathbf{z}_i) \mathcal{N}(\mathbf{z}_i|\bar{\mathbf{f}}_i, h_{f_i} I) d\mathbf{z}_i = \left(\int \frac{1}{\mathcal{Z}(\mathbf{z}_i)} \mathcal{N}(\mathbf{z}_i|\bar{\mathbf{f}}'_i, h_{f_i} I) \mathcal{N}(\mathbf{z}_i|\bar{\mathbf{f}}_i, h_{f_i} I) \, d\mathbf{z}_i \right) p(\bar{\mathbf{f}}'_i|v_i, \ell^2_i) \\ &= H(\bar{\mathbf{f}}', \bar{\mathbf{f}}) p(\bar{\mathbf{f}}'_i|v_i, \ell^2_i). \end{aligned} \tag{48}$$

Notice that by construction $H(\bar{\mathbf{f}}_i', \bar{\mathbf{f}}_i)$ is symmetric as promised. When the variance of the GP prior $p(\bar{\mathbf{f}}_i'|v_i, \ell_i^2)$ tends to infinity, step 1 reduces to $\bar{\mathbf{f}}_i' \sim \mathcal{N}(\bar{\mathbf{f}}_i'|\mathbf{z}_i, h_{f_i} I)$ and both steps combined yield $\bar{\mathbf{f}}_i' \sim \mathcal{N}(\bar{\mathbf{f}}_i|\bar{\mathbf{f}}_i, 2h_{f_i} I)$, which is the standard *random walk Metropolis* (RWM) algorithm. Therefore, the above algorithm can be thought of as a *Gaussian scaled RWM*, which generalises RWM so that a high dimensional and correlated Gaussian distribution is incorporated into RWM's proposal mechanism. The scale parameter h_{f_i} is tuned using preliminary runs in order to achieve an acceptance rate around 25%.

There exist may other algorithms that could be used for sampling the GP latent function vectors. In particular, in a previous version of our implementation a method based on control variables was used (Titsias et al., 2009). However, the current method is simpler and more efficient than control variables. Another scheme often considered is based on whitening the GP priors through a transformation (Kuss and Rasmussen, 2005). A more advanced method following the above idea is to whiten not with respect to the GP prior but with respect to a Gaussian approximation to the posterior distribution (Christensen et al., 2006) obtained, for instance, using the Laplace approximation. These ideas can be combined with the Metropolis adjusted Langevin algorithm (Christensen et al., 2006) or Hamiltonian Monte Carlo (Kuss and Rasmussen, 2005; Schmidt, 2009). Riemann Manifold extensions of these algorithm could also be considered (Girolami and Calderhead, 2011). However, we should point out that computing derivatives, and especially second derivatives, may be very computationally expensive since unlike many simple GP models, in our case the likelihood function has a complex non-factorising form. A simple algorithm to sample the latent function in GP models is elliptical slice sampling (Murray et al., 2010). However, this method is quite expensive in our case since it requires several likelihood evaluations when sampling each GP latent vector, and a likelihood evaluation in our model is the most costly operation during MCMC.

Finally sampling the GP latent vectors from the conditional posterior distribution while fixing the values of the kernel hyperparameters (especially the length scales) can be less efficient as discussed recently by Murray and Adams (2010). Whitening can be important for efficient sampling of the kernel hyperparameters in GP models and Murray and Adams (2010) combine this idea with slice sampling, while previously whitening based on the GP prior and joint sampling hyperparameters and latent variables with Hamiltonian Monte Carlo was also used by Schmidt (2009). Murray and Adams (2010) also consider surrogate data to improve sampling the kernel hyperparameters which is similar to our method with the difference that we use surrogate or pseudo data to sample the GP latent vectors.

B Sampling from factorised truncated Gaussians

Several MCMC steps in Section 3.5 make use of truncated Gaussian proposal distributions. These distributions are suitable for sampling non-negative parameters and obtain the following form

$$Q(\mathbf{w}'|\mathbf{w}) = \frac{\mathcal{N}(\mathbf{w}'|\mathbf{w}, \sigma^2 I) I(\mathbf{w}' \geq 0)}{\int \mathcal{N}(\mathbf{z}|\mathbf{w}, \sigma^2 I) I(\mathbf{z} \geq 0)\, \mathrm{d}\mathbf{z}}, \tag{49}$$

where $\mathbf{w}$ is a parameter vector. Sampling is efficiently performed using rejection sampling. Firstly notice that the above distribution factorises so as

$$Q(\mathbf{w}'|\mathbf{w}) = \prod_i \frac{\mathcal{N}(w_i'|w_i, \sigma^2) I(w_i' \geq 0)}{\int \mathcal{N}(z|w_i, \sigma^2) I(z \geq 0)\, \mathrm{d}z} = \prod_i Q(w_i'|w_i), \tag{50}$$

and thus we can sample independently from each truncated univariate Gaussian $Q(w'_i|w_i)$ using rejection sampling. More precisely, to sample from $Q(w'_i|w_i)$ we draw samples from the Gaussian $\mathcal{N}(w'_i|w_i, \sigma^2)$ until we obtain a $w'_i \geq 0$. This is very efficient since the probability of $w'_i \geq 0$ under the law $\mathcal{N}(w'_i|w_i, \sigma^2)$ is larger or equal to 0.5 because $w_i \geq 0$. Therefore, the number of trials needed until we get a non-negative w'_i follows a geometric distribution with a mean value less or equal to two. Notice that if we were applying rejection sampling directly in the multivariate distribution in Eq. (49), without taking into account the independence, that would be very inefficient as the probability of $\mathbf{w}' \geq \mathbf{0}$ goes to zero exponentially with the dimension of $\mathbf{w}$.

Other MCMC implementations sample non-negative model parameters in the log space using a Gaussian distribution which also requires the Jacobian of the transformation to be used. However, given that factorised Gaussian distributions are used in such implementations, we have observed in practice that the truncated Gaussians are equally fast and somehow more efficient as they sample the non-negative parameters in their natural space without causing numerical or MCMC convergence problems[2].

C Additional details about the algorithms and MCMC convergence

Here, we provide some additional details regarding running the MCMC algorithms and convergence assessment. As explained in the main part of this report there are two separate MCMC algorithms: the first applied in the training phase (see Section 3) and the second applied in the prediction phase (Section 4). We start with discussing the training phase case.

C.1 Training phase

The discretisation of the GP latent functions (together with the corresponding ODE integrals; see Eq. (12) and (13)), which need to be sampled in the training phase, is done as follows. The size of each GP vector $\bar{\mathbf{f}}_i$ is chosen to be around one order of magnitude larger that the number of time points of the mRNA data. To describe this, let us recall some notation introduced in Section 3.3 where $N_m = |\mathcal{T}_m|$ is the size of the mRNA data time points and $N_p = |\mathcal{T}_p|$ the size of the discretisation. In the *Drosophila* data, the 12 observed mRNA time points were equally spaced between 0 and 11, i.e. $\mathcal{T}_m = [0, 1, \ldots, 11]$ and $\mathcal{T}_p = [0, 0.1, 0.2, \ldots, 10.9, 11]$, thus each GP vector $\bar{\mathbf{f}}_i$ is 111-dimensional. In the artificial data, there are 10 observed mRNA time points, which are $\mathcal{T}_m = [0, 1, 2, 3, 5, 7, 9, 11, 14, 18]$. The corresponding set of the discretised GP functions was 181-dimensional so that $\mathcal{T}_p = [0, 0.1, 0.2, \ldots, 17.9, 18]$.

Let us now turn our attention to the number of parameters that we need to sample in the training phase. There are N_{tr} training genes used is this phase and the number of the parameters per gene is decomposed as follows. Four kinetic ODE transcription parameters (b_j, d_j, s_j, a_j), one bias w_{j0} in the sigmoid, one noise variance $\sigma^2_{m,j}$ and at most I interaction weights $\mathbf{w}_{\mathbf{x}_j}$. Additionally we have three sets of parameters per TF, i.e. the degradation rate δ_i and the kernel hyperparameters (ℓ_i^2, v_i). Therefore, in total the number of parameters is $6N_{tr} + CNI + 3I$, where $C \leq 1$. There are 814 such parameters in the *Drosophila* data and 237 in the artificial data. Together with those parameters, in the training phase we sample GP functions and this consists of 15 (5 TFs and 3 replicas) 111-dimensional vectors in *Drosophila* data and 6 (3 TFs and 2 conditions) 181-dimensional vectors in the artificial data.

[2]For instance, to sample a value close to zero of a non-negative parameter using a Gaussian that proposes values in the log space, we need to sample values close to minus infinity. If the posterior of this parameter is multimodal and a second mode exist away from zero, then the proposal distribution could be slow in driving the transition between the two modes.

Sampling requires the adaption of several parameters of the proposal distribution as explained in Section 3.5. We apply a simple procedure that learns based on the acceptance rate. The guideline we follow is that the acceptance rate of the algorithm should be around 25% and all scale parameters are tuned according to that rule. This is close to the value 0.234 which has been shown to be optimal for certain simple Metropolis-Hastings algorithms (Gelman et al., 1996; Roberts et al., 1996; Robert and Casella, 2004). This adaption process requires preliminary runs. More precisely, the adaptive process consists of a set of iterations. In each iteration we run MCMC algorithm (see Section 3.5) for a window of J ($J = 100$ in our implementation) and compute the empirical acceptance rates associated with each MCMC step. Then the scale parameters are updated using a standard stochastic approximation rule (Andrieu and Thoms, 2008). In the problems where we applied the algorithm, the adaption phase takes roughly 10^4 to 2×10^4 iterations and serves also as a burn-in (transient) phase of the MCMC. Then 10^5 iterations are used to collect 1000 posterior samples (we used thinned samples by keeping one sample every 100 iterations). A full run of the MCMC algorithm in *Drosophila* data takes about 30 hours real time in a Intel(R) Core(TM)2 CPU 6600 @2.40GHz.

Convergence of the algorithm in the training phase was monitored by running multiple independent chains and checking whether the Monte Carlo estimates of the different chains agree with one another. This was done mainly by computing 5% and 95% percentiles of the parameters separately for each chain, and then computing the overlap between the intervals across different chains. A large overlap implies that the chains have converged to the same Monte Carlo estimates. This is similar in fashion with certain statistics commonly used in the literature (Gelman and Rubin, 1992; Gelman et al., 2003). Visual inspection of the estimated parameters especially with regard to the latent GP functions was also considered. We should emphasise that obtaining empirical[3] convergence of MCMC algorithms in complex models having posterior distributions with multiple modes is very challenging. Our model is certainly a very complex one with potentially multiple modes in the posterior distribution. In such cases tempering and population MCMC strategies (Geyer, 1992; Earl and Deem, 2005; Calderhead and Girolami, 2009) can help speeding up convergence. However, such techniques are not currently exploited by our implementation.

C.2 Prediction phase

A single MCMC run used in the prediction phase is much simpler and faster. Specifically, the number of parameters needed to be sampled for a specific test gene and network configuration is $6 + CI$, where $C \leq 1$. For instance, in the *Drosophila* system we need to sample at most eleven parameters in a single MCMC run. Recall that we also sample the GP functions through "cache samples" as described in Section 4.1.1. The adaption phase (that includes the transient phase) tends to be fast, while in the stationary phase 3×10^4 samples are used to collect 3×10^3 samples for further use such as the computation of the predictive densities (see Section 4.1.2). The real running time of a single MCMC run was roughly 2 minutes in both the *Drosophila* and the artificial data.

References

Alon U, 2006. *An Introduction to Systems Biology: Design Principles of Biological Circuits.* Chapman and Hall/CRC, London.

[3]An empirically obtained convergence does not imply that an actual convergence has been obtained. This is because is rather impossible to know in general whether there exist or not additional modes of the posterior distribution that are not discovered yet by any of the chains. Only in simple models, e.g. where the posterior distribution is known to be unimodal, is reasonable to believe with high certainty that an empirically detected convergence means actual convergence.

Andrieu C and Thoms J, 2008. A tutorial on adaptive MCMC. *Stat Comput*, **18**:343–373.

Calderhead B and Girolami MA, 2009. Estimating Bayes factors via thermodynamic integration and population MCMC. *Comput Stat Data An*, **53**(12):4028–4045.

Chib S, 1995. Marginal likelihood from the Gibbs output. *J Roy Stat Soc B*, **90**(432):1313–1321.

Christensen OF, Roberts GO, and Sköld, 2006. Robust Markov chain Monte carlo methods for spatial generalized linear mixed models. *J Comput Graph Stat*, **15**:1–17.

Earl DJ and Deem MW, 2005. Parallel tempering: Theory, applications, and new perspectives. *Phys Chem Chem Phys*, **7**(23):3910–3916.

Friel N and Pettitt AN, 2008. Marginal likelihood estimation via power posteriors. *J Roy Stat Soc B*, **70**(3):589–607.

Gao P, Honkela A, Rattray M, and Lawrence ND, 2008. Gaussian process modelling of latent chemical species: Applications to inferring transcription factor activities. *Bioinformatics*, **24**(16):i70–i75.

Gelman A, Carlin J, Stern H, and Rubin D, 2003. *Bayesian Data Analysis.* Chapman and Hall/CRC, second edition.

Gelman A and Meng XL, 1998. Simulating normalizing constants: from importance sampling to bridge sampling to path sampling. *Stat Sci*, **13**(2):163–185.

Gelman A, Roberts GO, and Gilks WR, 1996. Efficient Metropolis jumping rules. *In Bayesian statistics*, **5**.

Gelman A and Rubin DB, 1992. Inference from Iterative Simulation Using Multiple Sequences. *Stat Sci*, **7**(4):457–472.

Geyer CJ, 1992. Practical Markov Chain Monte Carlo. *Stat Sci*, **7**(4):473–483.

Girolami M and Calderhead B, 2011. Riemann manifold Langevin and Hamiltonian Monte Carlo methods. *J Roy Stat Soc B*, **73**(2):123–214.

Honkela A, Girardot C, Gustafson EH, Liu YH, Furlong EEM, Lawrence ND, and Rattray M, 2010. Model-based method for transcription factor target identification with limited data. *Proc Natl Acad Sci USA*, **107**(17):7793–7798.

Kuss M and Rasmussen CE, 2005. Assessing Approximate Inference for Binary Gaussian Process Classification. *J Mach Learn Res*, **6**:1679–1704.

Marbach D, Prill RJ, Schaffter T, Mattiussi C, Floreano D, and Stolovitzky G, 2010. Revealing strengths and weaknesses of methods for gene network inference. *Proc Natl Acad Sci USA*, **107**(14):6286–6291.

Murray I and Adams RP, 2010. Slice sampling covariance hyperparameters of latent Gaussian models. In Lafferty J, Williams CKI, Shawe-Taylor J, Zemel RS, and Culotta A, editors, *Advances in Neural Information Processing Systems 23*, pages 1732–1740.

Murray I, Adams RP, and MacKay DJ, 2010. Elliptical slice sampling. *JMLR: W&CP*, **9**:541–548.

Neal RM, 1998. Annealed importance sampling. *Statistics and Computing*, **11**:125–139.

Newton MA and Raftery AE, 1994. Weighted likelihood bootstrap. *J Roy Stat Soc B*, **56**:3–48.

Pearson RD, Liu X, Sanguinetti G, Milo M, Lawrence ND, and Rattray M, 2009. puma: a Bioconductor package for propagating uncertainty in microarray analysis. *BMC Bioinformatics*, **10**:211.

Robert CP and Casella G, 2004. *Monte Carlo Statistical Methods.* Springer-Verlag, 2nd edition.

Roberts GO, Gelman A, and Gilks WR, 1996. Weak convergence and optimal scaling of random walk Metropolis algorithms. *Ann Appl Probab*, **7**:110–120.

Schmidt MN, 2009. Function factorization using warped Gaussian processes. In *International Conference on Machine Learning 26*, pages 921–928.

Titsias MK, Lawrence ND, and Rattray M, 2009. Efficient sampling for Gaussian process inference using control variables. In Koller D, Schuurmans D, Bengio Y, and Bottou L, editors, *Advances in Neural Information Processing Systems 21*, pages 1681–1688.

Tomancak P, Beaton A, Weiszmann R, Kwan E, Shu S, Lewis SE, Richards S, Ashburner M, Hartenstein V, Celniker SE, *et al.*, 2002. Systematic determination of patterns of gene expression during Drosophila embryogenesis. *Genome Biol*, **3**(12):RESEARCH0088.

Veitia RA, 2003. A sigmoidal transcriptional response: cooperativity, synergy and dosage effects. *Biol Rev Camb Philos Soc*, **78**(1):149–170.
